# Effects of transcutaneous electrical acupoint stimulation on early postoperative pain and recovery: a comprehensive systematic review and meta-analysis of randomized controlled trials

**DOI:** 10.3389/fmed.2024.1302057

**Published:** 2024-04-29

**Authors:** Shi-Yan Tan, Hua Jiang, Qiong Ma, Xin Ye, Xi Fu, Yi-Feng Ren, Feng-Ming You

**Affiliations:** ^1^Hospital of Chengdu University of Traditional Chinese Medicine, Chengdu, Sichuan Province, China; ^2^TCM Regulating Metabolic Diseases Key Laboratory of Sichuan Province, Hospital of Chengdu University of Traditional Chinese Medicine, Chengdu, Sichuan Province, China

**Keywords:** transcutaneous electrical acupoint stimulation, surgery, pain, recovery, meta-analysis

## Abstract

**Background:**

Previous studies have indicated beneficial outcomes of transcutaneous electrical acupoint stimulation (TEAS), but high-quality and comprehensive meta-analyses are lacking. The aim was to quantitatively analyze the efficacy and safety of perioperative TEAS on postoperative pain and recovery.

**Methods:**

PubMed, Web of Science, EMBASE, and the Cochrane Library were searched through July 2022. Randomized controlled trials (RCTs) that examined the perioperative application of TEAS in adults compared with sham-TEAS and/or non-TEAS were eligible. Cumulative analgesic consumption within 24 h and rest pain scores at 2, 6, 12, and 24 h postoperatively were the two co-primary outcomes.

**Results:**

Seventy-six RCTs (*n* = 9,665 patients) were included. Patients treated with TEAS experienced a reduction in clinical importance in cumulative analgesic (morphine equivalent) consumption (WMD: −14.60 mg, 97.5% CI: −23.60 to −5.60; *p* < 0.001) and a reduction in statistical importance in rest pain scores at multiple time points within the first 24 postoperative hours. The secondary outcome analysis also identified clinically significant recovery benefits to TEAS during the first 24 h after surgery. Furthermore, TEAS could effectively reduce opioid-related side effects and did not increase serious side effects.

**Conclusion:**

This article describes current evidence about TEAS intervention on early postoperative pain and recovery. The results support the effectiveness of TEAS, but more high-quality evidence of clinical applicability is also needed.

**Systematic review registration:**

PROSPERO (CRD42021249814).

## 1 Introduction

Procedure-related pain is the most common postoperative complication. It has been reported that approximately 86% of patients experience postoperative pain (75% of them have moderate-to-severe pain) (1), which severely influences postoperative rehabilitation and decreases quality of life and increases healthcare costs, creating a considerable economic burden for patients (2, 3). Currently, although opioids represent the most common treatment against acute postoperative pain (4), the analgesic benefits can be compromised by side effects, such as nausea, vomiting, and dizziness. Inspiringly, transcutaneous electrical acupoint stimulation (TEAS), as a representative non-pharmacologic technology, has gained much attention in recent years due to its unique advantages in perioperative analgesia (5–8). Specifically, TEAS is a more acceptable and attractive adjunctive intervention that combines the dual benefits of traditional Chinese acupoint and transcutaneous electrical nerve stimulation. More importantly, compared with electroacupuncture and traditional acupuncture, TEAS not only has a similar analgesic effect but also is characterized as simple, safe, and non-invasive (9–11).

Whereas research increasingly shows that TEAS has advantages with regard to perioperative pain, high-quality evidence is still lacking. It should be pointed out that one meta-analysis in 2016 used TEAS in a subgroup analysis to first quantitatively evaluate its treatment effects on postoperative pain, but the deficiencies that need to be acknowledged are that no additional individual time points were included in a comprehensive assessment, and the safety of TEAS was also not adequately studied (12). In addition, Meng et al. affirmed the short-term efficacy of TEAS for pain after laparoscopy (13). Although more rigorous and rational than previous meta-analyses in methods, the disadvantages of the study are also obvious. First, the quality of the included trials was low, and the review was obscured by methodological shortcomings or the small pool sample. Second, multimodal and single modalities of analgesia were mixed and hence exaggerated the actual outcomes of TEAS alone. Ultimately, their findings were limited by a lack of clinical interpretation based on the minimal clinically important difference (MCID) and a lack of adjustments in the statistical thresholds; that is, there was an increased risk of type I errors and multiple testing bias. Therefore, we sought a high-quality and comprehensive meta-analysis to examine the potential clinical benefits of TEAS in postoperative analgesia and recovery.

## 2 Methods

### 2.1 Search strategy

This review was performed in accordance with the Preferred Reporting Items for Systematic Reviews and Meta-Analyses (PRISMA) statement and Assessing the Methodological Quality of Systematic Reviews (AMSTAR) (14). Four English databases [PubMed, Web of Science (all databases), EMBASE, and the Cochrane Library] were searched from the inception of the databases to 10 July 2021 and updated the search on 9 July 2022. The search was not restricted by language. This meta-analysis was registered in the PROSPERO (CRD42021249814).

The search strategy was developed by an experienced medical information specialist. Our search algorithms combined Medical Subject Heading (MeSH) terms and corresponding free-text words, such as “transcutaneous electrical acupoint stimulation (TEAS),” “perioperative period (MeSH),” and “general surgery (MeSH).” Full search term strategies are shown in Supplementary Methods 1–5. Additionally, we searched the gray literature using Google Scholar and reviewed ongoing or recently completed trials at clinical trial registries (e.g., ClinicalTrials.gov).

### 2.2 Eligibility criteria and study selection

Our review only considered RCTs. Article type of comments, case reports, conference abstracts, letters, and other non-RCT studies related to TEAS therapy were excluded. Studies eligible for inclusion must have the following characteristics:

Participants: We included adults (≥18) scheduled for elective surgery with no restriction regarding the type of surgery and anesthesia. Patients undergoing emergency surgery were excluded.

Interventions: Patients must have received TEAS therapy as the sole intervention. TEAS imposes an electrical stimulation pulse to target acupoints by using electrodes placed on the acupoint surface. Regardless of the use of acupoints, treatment duration, or stimulus intensity in a trial, data from multiple intervention groups were pooled. Trials with intervention groups that were transcutaneous electrical nerve stimulation not involving acupoints or TEAS combined with auricular acupressure and another Traditional Chinese Medicine (TCM) therapy were excluded from this study.

Comparator: Two types of controls were considered as study comparators. For sham-TEAS, stimulation was performed at the non-acupoint with the same electricity parameters. Non-TEAS included the following: (i) only usual anesthesia; (ii) electrodes placed without electricity; (iii) imperceptible stimulus intensity around the sensory threshold.

Outcomes: Pain intensity (static) and opioid consumption were co-primary outcomes. As TEAS has been purported to provide extended analgesia for approximately 24 h, and this time interval was frequently reported in the literature (8, 15, 16), we primarily focused on day 1 (the interval from 0 to 24 h) analgesic outcomes to best capture the potential incremental benefits of TEAS and preserve statistical power. The secondary outcomes were other pain-related indicators, postoperative rehabilitation, and complications.

Only articles with available full-text and complete data were included. Two authors independently screened the titles and abstracts and reviewed full-texts. Any uncertainties or discrepancies were resolved through consultation with two separate reviewers.

### 2.3 Data extraction

Key information included the first author, countries where studies were run, year of publication, participants, intervention, and outcomes. Two reviewers independently extracted data using a uniform excel spreadsheet. Measures of postoperative pain intensity were all transformed to an equivalent 0–10 cm visual analog scale (VAS) score, with 0 corresponding to no pain and 10 corresponding to excruciating pain (17, 18). All opioid consumption was converted into cumulative intravenous morphine equivalent consumption (mg) (10 mg intravenous morphine = 10 mg intravenous dezocine = 0.01 mg intravenous sufentanil) (11, 19). If there were missing or unclear data from the included studies, we tried to contact the authors to clarify the methods or provide additional data as needed.

### 2.4 Interpretation of outcome results

Regarding postoperative pain scores, the magnitude of a 1-cm decrease in the rest pain assessment at a single time point was considered clinically significant (20). Regarding cumulative opioid consumption, we considered a difference of 10 mg of intravenous morphine to be clinically important (17). For the recovery-40 (QoR-40) scores ranged from 40 (extremely poor recovery) to 200 (excellent recovery) (21), the change of 6.3 points explains the clinical effect of treatment (22).

### 2.5 Assessment of methodological quality and risk of bias

The RCTs were assessed for potential bias by employing the Risk of Bias 2 (RoB 2) tool (Supplementary Table 1) (23, 24). An overall high bias risk was determined if one or more domains were high. Moreover, if the majority of the five categories (that is three or more out of five) were considered at unclear risk of bias, the trial was also considered to be at risk of bias. We used Grading of Recommendations Assessment, Development, and Evaluation (GRADE) Profiler 3.6 software to summarize the quality of evidence and formulate recommendations (Supplementary Table 2) (25). Quality assessments were performed by two independent investigators, with disagreements resolved by a third reviewer.

### 2.6 Statistical analysis

Data analysis was carried out using Review Manager V5.4 (Cochrane Collaboration, Nordic Cochrane Centre, London, United Kingdom).

For continuous variables, data are presented as the mean value ± standard deviation (SD). When these were not available, we contacted the corresponding author for additional data. If no response was received, data in other forms [i.e., the median and interquartile range (IQR), median, and range] were used to approximate the mean and SD. Finally, we used GetData graphics digitizer software (GetData Pty Ltd., Kogarah, Australia) to extract data that were only graphically reported (26). Statistical pooling was only adopted for the data available from at least two studies. Outcomes with less than two studies are presented qualitatively (18).

Statistical heterogeneity among studies was evaluated through the use of *I*^2^ statistics. When *I*^2^ < 50%, the fixed effects model was adopted. Otherwise, a random effects model was utilized. When this threshold was present in the primary outcomes, meta-regression analysis based on mixed effects model was conducted to explore independent sources of heterogeneity among studies.

Meta-regression analysis was only performed when at least 4 studies were included in the pooled effect estimate and each covariate subgroup included at least 2 trials (18, 27). In particular, an R2 value (coefficient of determination) was calculated to help quantify each variation in data between covariates (17). The covariates based on baseline characteristics considered were (i) types of anesthesia; (ii) types of surgery; (iii) intervention modes in the control group; (iv) the implementation of TEAS timing; and (v) protocol registered or not. Except pre-specified indicators, a subgroup analysis would be performed for trials with low bias risk in comparison with trials with unclear/high bias risk.

As there is a concern that evidence from trials with a high risk of bias may introduce bias, we also planned a sensitivity analysis on this variable.

When the primary outcomes were reported in 10 or more studies, we assessed possible publication bias by visual examination of funnel plots, and the influence of significant publication bias on outcome indicators was evaluated by Egger tests, with *p* < 0.05 indicating significant bias (28). Publication biases were performed with Stata software V. 16.0 (StataCorp, College Station, TX).

## 3 Results

### 3.1 Search results and study characteristics

**Figure 1:**
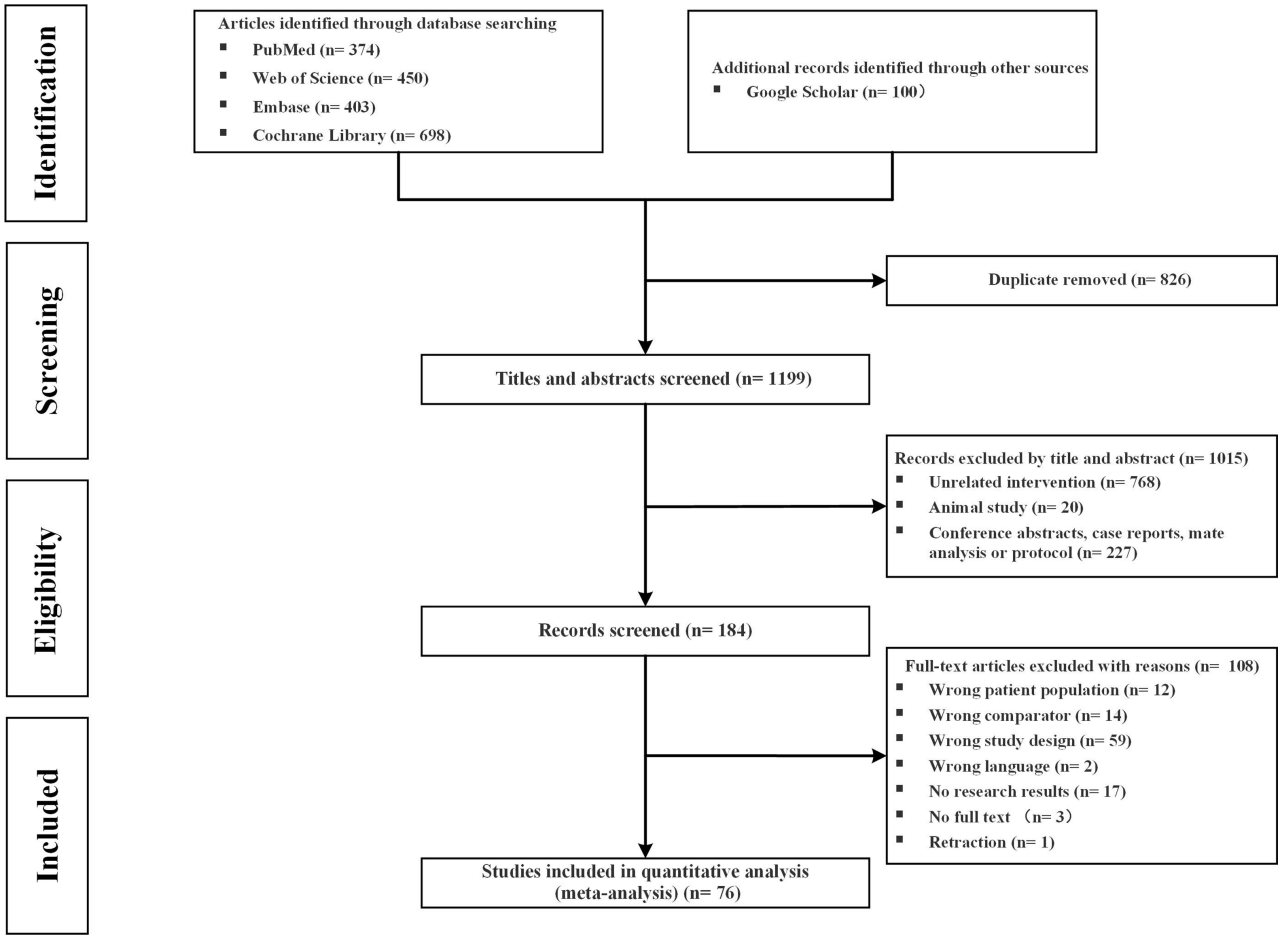
Search strategy and final included and excluded studies.

A total of 2,025 citations were identified by using the proposed search strategy. After removing duplicates, 1,199 studies were reviewed for title and abstract screening. Of those, 184 studies underwent full-text review. With reference to our inclusion and exclusion criteria, 76 studies (9,665 patients) were included for quality evaluation and quantitative analysis (Figure 1).

The basic characteristics of the included studies are presented in Supplementary Table S1. Sixty six included studies (86.8%) were conducted in China, followed by America (5) and Turkey (2). RCTs were diverse, with surgical subspecialties consisting of urology/andrology (3), gynecology (12), breast surgery (6), cardiothoracic surgery (8), Hemorrhoidectomy (1), otolaryngology (2), neurosurgery (7), head and neck surgery (5), abdominal surgery (24), orthopedic surgery (6), and hepatobiliary + gynecology surgeries (2). The different frequencies, acupoints, and times of intervention also presented the clinical diversity of TEAS. Of these, a frequency of 2/100 Hz (29) was the most commonly selected. The top three acupoints were, in turn, Neiguan (PC6) (in combination, 45; alone, 11), Hegu (LI4) (in combination, 40; alone, 2), and Zusanli (ST36) (in combination, 33; alone, 5). Specifically, five species of time to TEAS intervention were summarized in our review.

### 3.2 Risk of bias

**Figure 2:**
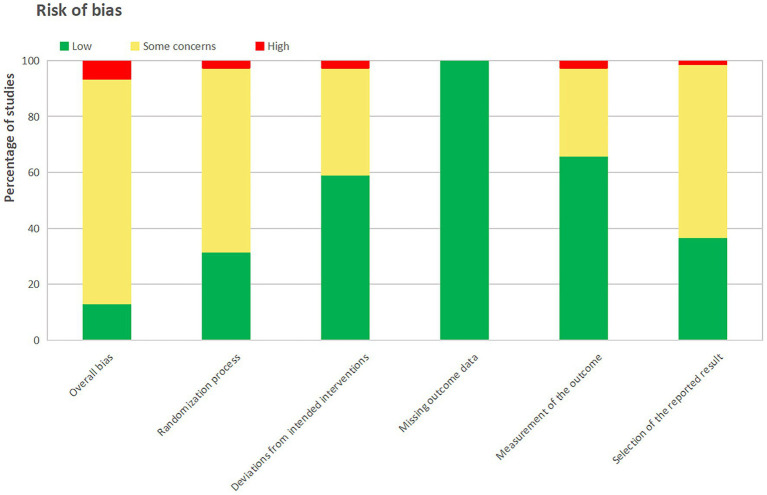
Summary of risk of bias assessment across all included randomized controlled trials.

The risk of bias assessment for all 76 included studies is presented. In total, 10 studies (13.16%) had overall low bias risks, 61 studies (80.26%) had some concern about bias overall, and 5 studies (6.58%) had overall high bias risks (Figure 2; Supplementary Figure 1).

### 3.3 Co-primary outcomes

#### 3.3.1 Cumulative intravenous morphine equivalent consumption (mg) within 24 h after surgery

Six studies (TEAS: 187, control: 268) (30–35) provided data on cumulative intravenous morphine equivalent consumption in the first 24 postoperative hours. The pooled analysis showed that cumulative intravenous morphine equivalent consumption in the TEAS group was lower than that in the control group, with a WMD of −14.60 mg (97.5% CI, −23.60 to −5.60) (*p* < 0. 001, *I*^2^ = 97%), which convincingly exceeded the threshold for clinical significance (10 mg).

With this pooled outcome, meta-regression failed to identify sources of heterogeneity. After removing one trial with a high risk of bias, we still obtained robust results (Supplementary Table 3). For the intervention of the control group, the treatment effect of sham-TEAS lost statistical significance (WMD: −5.33 mg, 97.5% CI: −6.36 to −4.30; *p* = 0.41) compared with TEAS interventions. In addition, further subgroup analyses showed no significant reduction in rescue analgesic morphine consumption in the minimally invasive surgery subgroup and the registered protocol subgroup, according to prespecified covariates (Supplementary Table 4). The quality of the evidence was low.

#### 3.3.2 Rest pain scores at 2, 6, 12, and 24 h postoperatively

**Figure 3:**
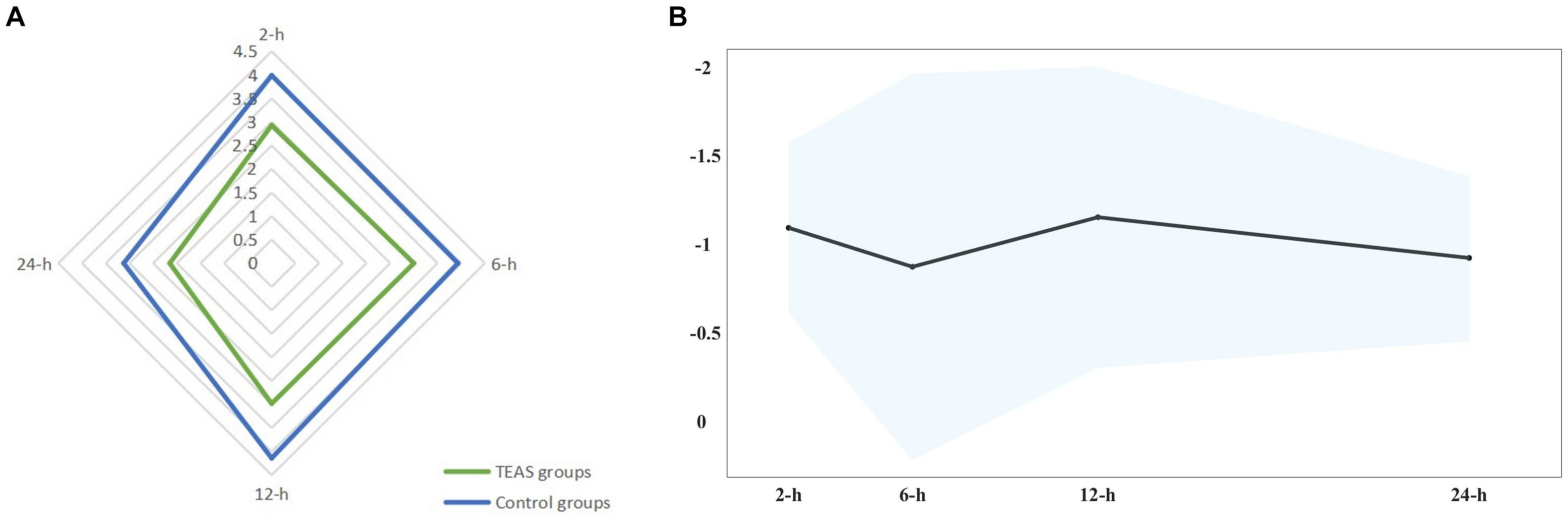
**(A)** Star plot for weighted mean of pain scores within 24 h after surgery at four time points in TEAS and control groups. **(B)** Band plot for WMD of pain scores within 24 h after surgery at four time points between TEAS versus control. Pooled estimates of the WMD for each time point are represented by the dark line, and 99% CIs are represented by surrounding shaded region.

The meta-analysis was finally conducted on 24 studies (6, 8, 15, 16, 29–32, 36–51). Overall, other than 6 h, TEAS reduced the rest pain scores at 2 h (WMD: −0.96 cm, 99% CI: −1.44 to −0.48), 12 h (WMD: −1.02 cm, 99% CI: −1.87 to −0.17), and 24 h (WMD: −0.79 cm, 99% CI: −1.25 to −0.32). Although these results were statistically significant, the differences only at 12 h surpassed the predesignated threshold for the clinical importance of 1.0 cm on the VAS (Figure 3; Supplementary Table S2).

**Figure 4:**
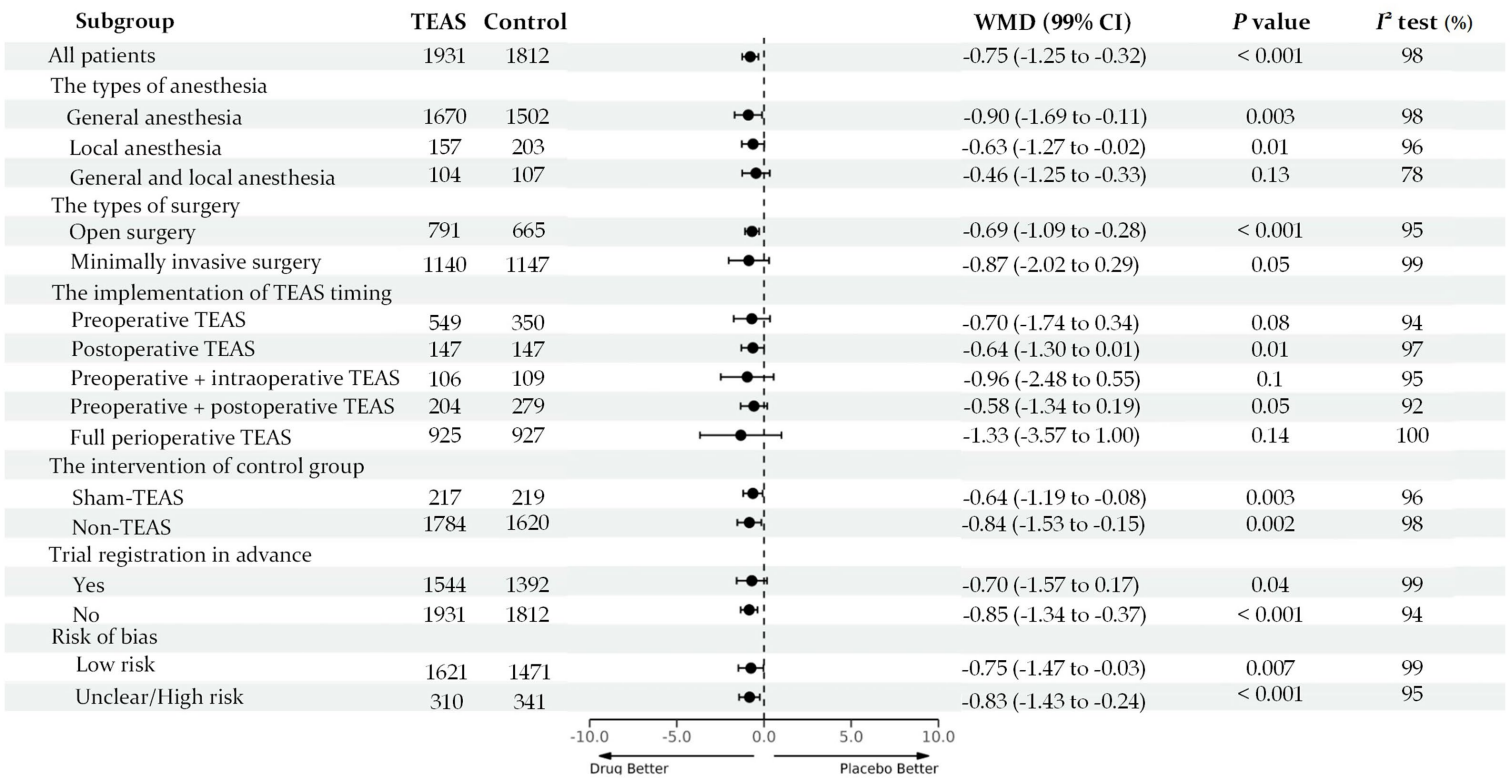
Forest plot for all subgroup analysis of rest pain score 24 h after surgery.

Meta-regression also indicated no confounding by the covariates. When the studies rated as having a high risk of bias were removed from the sensitivity analyses, the overall results remained robust at each time point. Notably, in the local anesthesia subgroup and combined anesthesia subgroup, no substantial differences in pain intensity between the TEAS group and the control group were observed [e.g., rest pain at 24 h: WMD: −0.63 points (99% CI: −1.27 to 0.02); WMD: −0.46 points (99% CI: −1.25 to 0.33)]. In the protocol registered subgroup, no statistical differences in pain intensity were also observed [e.g., rest pain at 2 h: WMD: −0.62 points (99% CI: −1.29 to 0.05); rest pain at 24 h: WMD: −0.70 points (99% CI: −1.57 to 0.17)]. Other subgroups (surgery types, intervention modes in the control group, time of intervention, and risk of bias) on the effect of TEAS at various time points had conflicting results at different time points (Figure 4; Supplementary Table 4). The evidence quality was low to moderate.

### 3.4 Secondary outcomes

#### 3.4.1 Perioperative pain-related indicators

Five studies (400 patients) suggested a significant reduction in cumulative intravenous morphine equivalent consumption within 48 h in the TEAS group versus control group (31, 32, 52–54), with a WMD of −20.20 mg (95% CI: −28.06 to −12.33), which has important clinical implications. Summary estimates were based on evidence of moderate quality.

For rest pain intensity over 24 h, no clinically meaningful difference was defined for adding TEAS interventions to treatment at 48 h (WMD: −0.57 points, 99% CI: −0.97 to −0.18), and no statistical difference was defined for TEAS interventions at 72 h between groups (Supplementary Table S3). The quality of evidence was very low and low, respectively.

After surgery in 6 and 3 studies with data available, the remaining studies revealed that TEAS reduced the rescue analgesic rate within 24 h (RR: 0.53, 95% CI: 0.38 to 0.74) and 48 h (RR: 0.45, 95% CI: 0.29 to 0.70), respectively. The quality of evidence for both was rated as high.

As for the intraoperative consumption of sedatives and opioid consumption, TEAS decreased the intraoperative consumption of remifentanil (WMD: −128.41 μg, 95% CI: −183.28 to −73.55) compared with the control groups. The level of propofol (mg), fentanyl (μg), and sufentanil (μg) did not differ between two groups (Supplementary Table S3). The quality of evidence was low to moderate.

A total of 9 studies (*n* = 1,196) were included in the intraoperative consumption of sedatives [tumor necrosis factor-α (TNF-α), interleukin-6 (IL-6), and noradrenaline (NE)] at 24, 48, and 72 h after surgery (7, 41, 49, 50, 53, 55–58). TNF-α was lower in the TEAS group at 48 h postoperatively (WMD: −15.91 pg./mL, 99% CI: −27.89 to −3.93). For IL-6, TEAS was superior to the control group at 24 h (WMD: −12.05 pg./mL, 99% CI: −15.86 to −8.23) and 72 h (WMD: −7.37 pg./mL, 99% CI: −13.90 to −0.84). In addition, the pooling of the included studies found that two groups did not differ for other end points (Supplementary Table S3). The overall quality of evidence ranged from very low to moderate.

#### 3.4.2 Rehabilitation-related outcomes

A significant difference in quality of QoR-40 scores (WMD: 10.64, 95% CI: 6.14–15.14) met the threshold for clinical significance when TEAS treatment was compared with control treatment at 24 h after surgery; while the difference was not clinically meaningful at 48 h after surgery (Supplementary Table S3). The quality of evidence was moderate and high, respectively.

As for gastrointestinal symptoms, a significant decrease in time (hours) to first flatus (WMD: −11.17, 95% CI: −15.35 to −7.00), defecation (WMD: −15.88, 95% CI: −21.15 to −10.62), feeding (WMD: −10.64, 95% CI: −18.01 to −3.27), and bowel sound (WMD: −4.86, 95% CI: −6.25 to −3.46) occurred when TEAS was compared with the control groups. No difference in time to first ambulation was found between the two groups (Supplementary Table S3). For these outcomes, the quality of evidence was very low to high.

Data from 21 studies (2,151 participants) revealed differences in the hospital length of stay in the TEAS versus control group (WMD: −0.98 days, 95% CI: −1.37 to −0.59).

### 3.5 Safety outcomes

#### 3.5.1 Postoperative opioid-related side effects

Postoperative opioid-related side effects include six major aspects [postoperative nausea (PON), postoperative vomiting (POV), postoperative nausea and vomiting (PONV), pruritus, dizziness, and rescue antiemetic rate] within the following postoperative periods: 0 to 24 h and 0 to 48 h.

Within 24 h, the results showed that TEAS could reduce all the incidence of PON (RR: 0.63, 95% CI, 0.53–0.76), POV (RR: 0.63, 95% CI, 0.54–0.73), PONV (RR: 0.67, 95% CI, 0.61–0.73), dizziness (RR: 0.56, 95% CI, 0.41–0.77), and rescue antiemetic rate (RR: 0.66, 95% CI, 0.53–0.82). Evidence quality was rated as low to moderate. However, in the TEAS and control groups, only PONV was significantly different after 48 h of surgery. As for pruritus, there was no significant benefit of TEAS in any of clinical outcomes between the two time periods (Supplementary Table S3). The quality of evidence was relatively reliable.

#### 3.5.2 TEAS-related potential adverse effects

A total of 10 studies specifically reported no TEAS-related adverse events during the study period. However, 7 studies (*n* = 1,214) reported skin irritation in 15 patients (8, 33, 38, 54, 59–61) and 1 study reported sleep disorders in 17 patients (62). Most symptoms were mild and were spontaneously resolved.

## 4 Discussion

While there is an increasing body of evidence to suggest the benefits of electrical stimulation at acupoints, this is the largest and most comprehensive systematic review and meta-analysis to elucidate the potential role of TEAS in providing perioperative analgesia and rehabilitation based on the context of enhanced recovery after surgery (ERAS). Specifically, TEAS has clinically relevant analgesic and recovery benefits during the first 24 postoperative hours, as demonstrated by similar overall pain scores, opioid consumption, rehabilitation-related outcomes and opioid-related side effects. Furthermore, qualitative evidence demonstrated that the application of TEAS did not increase the risk of skin irritation or sleep disorders. These findings support TEAS as part of a perioperative multimodal analgesia approach to improve early postoperative acute pain and recovery. However, the quality of evidence for the main outcome was rated as low, which might be a major barrier to the use of TEAS in clinical practice.

Compared with existing meta-analyses on the same topic (12, 13), our study included more patients, provided more rigorous statistical methods, and overcame the study design limitations of previous studies. Most studies noted a significant benefit of perioperative TEAS, especially improving postoperative pain on the first day after surgery, while we first proposed that the effect might be concentrated in the early postoperative phase. In this study, low-quality evidence showed that TEAS was associated with a reduction in opioid consumption, and such influence had strong clinical value. A possible explanation of validity was that all the included studies would use TEAS in the postoperative period, which effectively reduced the need to consider prescribing opioids in the first place and avoided prolonged opioid use (63). Remarkably, the management of postoperative opioid consumption within 48 h was significantly clinically less with TEAS in our study, which further supported such accumulated advantage. Although two existing meta-analyses identified the reduction in postoperative analgesic consumption in the TEAS group, such a cumulative effect has not been observed in opioid using. As for the statistical differences of reduction in pain scores at most selected time points, it was highly likely to be the mild (1–3 points) painful sensation of patients included or great variation of surgical details. While pre-specified study characteristics do not account for the observed heterogeneity, we also evaluate the validity of a subgroup effect based on the pre-protocol. Comparable results were obtained across the pre-specified subgroups: TEAS is more applicable to surgery under general anesthesia compared with other anesthetic methods. In addition, the inclusion of studies without pre-existing protocol registration may overstate the true efficacy of TEAS. A possible explanation is that this part of the study lacks a strict methodological approach (e.g., true randomization, concealed allocation, or blinding) which is not detailed in the text. As a whole, the overall certainty of evidence for all primary outcomes was of low quality based on our GRADE assessment, which mainly due to important methodological limitations and unexplained heterogeneity in the included studies, leading to imprecision and inconsistency, and caution is warranted when considering the main results.

It should be pointed out that although TEAS as adjuvant therapy in various operative procedures has been widely used to guide clinical practice (36), the specific analgesic mechanism is not fully understood. We assessed the levels of NE, IL-6, and TNF-α, which could significantly reflect the extent of stress and inflammation after surgery-related trauma (64), but these results were not statistically significant. Owing to the very low to moderate quality of evidence, a meticulous evaluation of neural mechanism underlying TEAS analgesia in future RCTs for confirmation is required.

TEAS regulates gastrointestinal function bidirectionally, and its perioperative application can promote gastrointestinal hormone secretion and accelerate intestinal peristalsis (65, 66). Our results supported this notion, finding significant improvements in first bowel movements, flatulence, bowel movements, and feeding with TEAS. It is obvious that TEAS can help maximize postoperative recovery, but the efficacy end point was not persisting in this review. Our outcomes of both global QoR-40 scores and opioid adverse events within 24 h postoperatively have likely supported the short-term efficacy of TEAS.

All studies included in this review supported the idea that TEAS was a safe method, inducing only mild adverse effects, such as local redness, swelling, and itching, in less than 20 patients. No one required specific treatment, and all abnormalities of the skin begin resolving within 24 h after removing electrodes. In addition, although Wang et al. reported sleep disorders due to cutaneous electrodes and wires (62), existing novel devices have circumvented the issue. Overall, TEAS, precluding unwanted side effects such as infection due to needle manipulation, is convenient and non-invasive for clinical applications.

Our review has several strengths. First, we formulated an exhaustive search strategy and produced the most inclusive knowledge synthesis that finally included 76 trials and more than 9,000 patients. As a result, we were able to provide higher quality evidence for clinically important outcomes (such as pain analysis and analgesic consumption) which have received more attention to the short-term efficacy of TEAS and enhanced the overall accuracy. Second, merged data were analyzed and interpreted based on clinically important differences, enhancing the efficacy assessment of TEAS. Finally, by adjusting statistical thresholds, we were able to minimize the risk of type I errors and multiple testing bias.

The current study also has some potential limitations. First, except for high statistical heterogeneity, high clinical heterogeneity was another important factor that might affect clinical mentoring intervention. As shown in Supplementary Table S1, intervention characteristics differed significantly between studies, including types of surgical subspecialties, the needling sites and the frequency, duration, and timing of TEAS, which might be potential sources of clinical heterogeneity among the studies. However, most often therapy for acupuncture is empirical. Unless the common international criteria are specified, it is difficult for us to use unified acupoints and stimulus frequencies to probe the underlying heterogeneities. Second, more than half of the included trials did not provide registered protocols or describe a clear randomization process, and the discordance of methodology has an unknown potential to impact the effectiveness of our results. Third, 86.8% included studies (66/76) were conducted in China, which meant the results may be more applicable to the Chinese populations. In addition, smaller sample sizes, variable study quality, and most of negative results rarely reported restricted the global generalization of our findings. Fourth, long-term follow-up data were limited, and we could not evaluate whether there was clinically important long-term efficacy of TEAS treatment. Fifth, we did not research Chinese-language databases and might miss some publications. This is in view of the fact that there is no real randomization in most of the relevant clinical studies in the Chinese language database, and the methods of analysis and data quality in these studies have many shortcomings. In the original study design, similar to the previous studies (67–72), we conservatively selected only four commonly used English-language databases to vouch for the completeness of the data, according to the convention. Such a search strategy was in complete agreement with our pre-registered protocol and could give guarantee of research reliability. However, we also believe that, if the quality of articles in Chinese-language databases could be further improved, breakthroughs in TEAS research are expected in the future.

## 5 Conclusion

In conclusion, we find the transient effects of TEAS, which focused on lower pain reported by patients, less opioid consumption, and higher quality of life during first 24 postoperative hours. TEAS, as a safe and pragmatic intervention, deserves broad generalization across various surgical subspecialties and procedures. Especially, in our study, TEAS analgesia may be more beneficial for surgeries under general anesthesia. However, within the limitations of the available evidence, large-scale randomized controlled trials with high methodological quality and prior outcomes are needed.

## Data availability statement

The original contributions presented in the study are included in the article/Supplementary material, further inquiries can be directed to the corresponding author/s.

## Author contributions

S-YT: Conceptualization, Formal analysis, Project administration, Software, Visualization, Writing – original draft, Writing – review & editing. HJ: Data curation, Formal analysis, Methodology, Visualization, Writing – original draft, Writing – review & editing. QM: Data curation, Formal analysis, Investigation, Validation, Visualization, Writing – original draft, Writing – review & editing. XY: Data curation, Formal analysis, Investigation, Validation, Visualization, Writing – review & editing. XF: Investigation, Methodology, Resources, Supervision, Writing – review & editing. Y-FR: Conceptualization, Project administration, Supervision, Validation, Visualization, Writing – review & editing. F-MY: Conceptualization, Project administration, Supervision, Validation, Writing – review & editing.
