# Supplementary material for: Effects of transcutaneous electrical acupoint stimulation on early postoperative pain and recovery: a comprehensive systematic review and meta-analysis of randomized controlled trials
Source: Front Med (Lausanne). 2024 Apr 29;11:1302057. doi: 10.3389/fmed.2024.1302057 (PMC11092893; doi:10.3389/fmed.2024.1302057)
Supplement: Supplementary file 1 [file Data_Sheet_1.ZIP › Supplementary material/Supplementary material 1.docx]

Supplementary Material

Effects of transcutaneous electrical acupoint stimulation on early postoperative pain and recovery: A comprehensive systematic review and meta-analysis of randomized controlled trials

**Shi-Yan Tan ^1†^, Hua Jiang ^1†^, Qiong Ma ^1†^, Xin Ye^1^, Xi Fu^1^, Yi-Feng Ren^1*^, Feng-Ming You^1*^**

*** Correspondence:** Feng-Ming You: [yfmdoc@163.com](mailto:yfmdoc@163.com); Yi-Feng Ren: [ryftcm.dr@yahoo.com](mailto:ryftcm.dr@yahoo.com)

| **Table 1. Study Characteristics** | | | | | | | | | | |
| --- | --- | --- | --- | --- | --- | --- | --- | --- | --- | --- |
| **Reference** | **Country** | **Anesthesia Method** | **Surgical Specialty** | **Age (years)** | **n= TG** | **Frequency of**  **TEAS (Hz)** | **Acupoint of TEAS** | **Time of TEAS** | **n= CG** | **Type of CG** |
| Ao et al. (2021) | China | GA | Breast surgery | 20-65 | 32 | 2/100 | LI4, PC6, ST36 | TST | 33 | Non-TEAS |
| Arnberger et al. (2007) | Austria, Switzerland | GA | Abdominal surgery and gynecology | 18-80 | 110 | 10 | PC6 | TSS | 110 | Sham-TEAS |
| Bai et al. (2018) | China | GA | Neurosurgery | 60-70 | 37 | 2/10 | LI4, PC6, LU7, LU5, LI18, ST9 | TTS | 38 | Non-TEAS |
| Chen et al. (2020) | China | GA | Cardiothoracic surgery | 18-64 | 40 | 2/100 | LI4, PC6, SI3, TE6 | TTT | 40 | Non-TEAS |
| Chen et al. (2018) | China | GA | Abdominal surgery | N/A | 33 | 25, 100 | ST36, PC6 | SST | 30 | Non-TEAS |
| Chen et al. (1998) | America | GA | Gynecology | N/A | 25 | 2/100 | ST36 | SST | 75 | Non-/sham-TEAS |
| Chen et al. (2013) | China | GA | Neurosurgery | 18-60 | 30 | 2/100 | LI4, TE5, BL63, LR3, ST36, GB40 | TTS | 60 | Non-/sham-TEAS |
| Chen et al. (2015) A | China | GA | Head and neck surgery | 18-60 | 41 | 2/10 | LI4, PC6 | TSS | 42 | Non-TEAS |
| Chen et al (2015) B | China | GA | Head and neck surgery | 18-60 | 29 | 2/10 | LI4, PC6 | TSS | 30 | Non-TEAS |
| Chi et al (2019) | China | LA | Orthopedic surgery | 65-85 | 26 | 2/10 | ST36, SP6, PC6 | TST | 26 | Non-TEAS |
| Chiu et al (1999) | China | LA | Hemorrhoidectomy | N/A | 30 | 2-100 | LI4, LU7 | SST | 30 | Sham-TEAS |
| Ertas et al. (2015) | Turkey | GA | Gynecology | 18-50 | 31 | 31 | PC6 | SST | 31 | Non-TEAS |
| Gan et al. (2004) | America | GA | Breast surgery | N/A | 26 | 2/100 | PC6 | TTS | 24 | Non-TEAS |
| Gao et al. (2018) | China | GA | Orthopedic surgery | ≥ 65 | 32 | 2/100 | PC6, LI4 | TTS | 32 | Non-TEAS |
| Gao et al. (2020) | China | GA | Urology/andrology | 18-70 | 29 | 2/15 | ST4736, SP6, RN3, RN4 | TSS | 28 | Non-TEAS |
| Gao et al. (2021) | China | GA | Abdominal surgery | ≥ 18 | 303 | 2/10 | ST36, ST37, SP6 | SST | 307 | Non-TEAS |
| Gao et al. (2022) | China | GA | Abdominal surgery | 18-50 | 827 | 2/10 | PC6, ST36 | TTT | 828 | Non-TEAS |
| Gao et al. (2017) | China | LA | Head and neck surgery | N/A | 34 | 2/100 | LI4, PC6 | TSS | 34 | Non-TEAS |
| Ge et al. (2021) | China | GA+ LA | Orthopedic surgery | 66-77 | 42 | 2/100 | ST36, SP9, SP10, GB34 | SST | 42 | Non-TEAS |
| Gu et al. (2019) | China | GA | Abdominal surgery | N/A | 58 | 2/100 | ST36, PC6 | TTT | 59 | Non-TEAS |
| Guo et al. (2018) | China | GA | Abdominal surgery | 18-70 | 30 | 2 | PC6, ST36, LI4 | TTT | 30 | Non-TEAS |
| Habib et al. (2006) | America | LA | Gynecology | N/A | 47 | N/A | PC6 | TST | 44 | Sham-TEAS |
| He et al. (2008) | China | GA | Breast surgery | 20-60 | 30 | 2/100 | PC6, LI4 | TTS | 30 | Non-TEAS |
| Huang et al. (2017) A | China | GA | Gynecology | 18-65 | 103 | 2/100 | LI4, PC6 | TSS | 224 | Non-TEAS |
| Huang et al. (2017) B | China | GA | Cardiothoracic surgery | N/A | 60 | 2/100, 2, 100 | PC6, LI4, LU7, LI11 | TST | 20 | Non-TEAS |
| Huang et al. (2018) | China | GA+ LA | Abdominal surgery | N/A | 32 | 2/10 | ST36 | TTS | 35 | Non-TEAS |
| Huang et al. (2019) | China | GA | Abdominal surgery | 18-75 | 29 | 2/100 | ST36 | TTS | 28 | Non-TEAS |
| Jin et al. (2022) | China | GA | Gynecology | 18-65 | 46 | 2/100 | LI4, PC6 | TSS | 46 | Non-TEAS |
| Lan et al. (2012) | China | LA | Orthopedic surgery | ≥ 65 | 30 | 2/100 | LI4, PC6, ST36, GB31 | TST | 30 | Non-TEAS |
| Li et al. (2016) | China | GA | Abdominal surgery | 18-80 | 29 | 2/10 | PC6, LI4, SP6, ST36 | TST | 29 | Non-TEAS |
| Li et al. (2020) A | China | GA | Abdominal surgery | 25-60 | 105 | 2/100 | PC6, LI4, ST36, SP6 | TTS | 35 | Non-TEAS |
| Li et al. (2020) B | China | LA | Gynecology | ≥ 18 | 54 | 25 | ST36 | SST | 54 | Sham-TEAS |
| Li et al. (2021) | China | GA | Abdominal surgery | 18-70 | 140 | 2/100 | LI4, PC6, ST36, ST37 | TTT | 140 | Non-TEAS |
| Liang et al. (2021) | China | GA | Urology /andrology | 18-64 | 35 | 2/100 | RN7, RN6, RN5, RN4, RN3 BL32, BL33, BL34 | TSS | 35 | Non-TEAS |
| Liu et al. (2021) | China | GA | Abdominal surgery | ≥ 65 | 50 | 2-100 | LI4, PC6, ST36 | TTS | 50 | Non-TEAS |
| Liu et al. (2015) | China | GA | Neurosurgery | 18-60 | 44 | 2/100 | LI4, TE5, BL63, LR3, ST36, GB40, GB20, BL10, BL2, EX-HN4 | TTS | 44 | Non-TEAS |
| Liu et al. (2008) | China | GA | Abdominal surgery | 18-60 | 48 | 2-100 | PC6 | TTS | 48 | Non-TEAS |
| Lu et al. (2021) | China | GA | Breast surgery | 18-65 | 388 | 2/10 | PC6; PC6, CV17 | TSS | 188 | Non-TEAS |
| Mi et al. (2018) | China | GA | Abdominal surgery | 18-65 | 50 | 2/100 | LI4, PC6, ST36 | TTS | 50 | Non-TEAS |
| Mu et al. (2019) | China | LA | Gynecology | 22-35 | 55 | 30/60 | ST36 | SST | 55 | Non-TEAS |
| Oztas et al. (2019) | Turkey | GA | Abdominal surgery | ≥ 18 | 15 | 2-100 | ST25, PC6, ST36, LI4 | SST | 32 | Non-/sham-TEAS |
| Que et al. (2021) | China | GA | Urology/andrology | N/A | 30 | 2/100 | BL23, SP9, LI4, PC6 | TTS | 30 | Non-TEAS |
| Si et al. (2009) | China | GA | Gynecology | 20-56 | 60 | 2/100 | PC6, LI4, PC8, SJ5 (GB21) | TSS | 30 | Non-TEAS |
| Song et al. (2020) | China | GA | Cardiothoracic surgery | 40-65 | 42 | 2/10 | HT7, PC6, ST36, LI4 | TST | 43 | Sham-TEAS |
| Sun et al. (2017) | China | GA | Abdominal surgery and Gynecology | 18-70 | 271 | 2/10 | LI4, PC6 | TSS/TTS/TST | 90 | Non-TEAS |
| Szmit et al. (2021) | Poland | GA | Abdominal surgery | 18-75 | 24 | 2/10 | LI4 | SST | 47 | Non-TEAS |
| Tu et al. (2018) | China | GA | Cardiothoracic surgery | ≥ 18 | 72 | 2/100 | BL13, LI4, ST36 | TSS | 72 | Non-TEAS |
| Wang et al. (1997) | America | GA | Abdominal surgery | N/A | 50 | 2/100 | LI4 | SST | 51 | Non-TEAS |
| Wang et al. (2017) | China | GA | Head and neck surgery | N/A | 50 | 2/100 | LI4, PC6 | TSS | 50 | Non-TEAS |
| Wang et al. (2014) | China | GA | Otolaryngology | 29-60 | 30 | 29 | LI4, PC6, ST36 | TSS | 30 | Non-TEAS |
| Wang et al. (2008) | China | GA | Neurosurgery | 20-67 | 25 | 2/100 | LI4, LI11, ST36, SP6 | TTS | 25 | Non-TEAS |
| Wang et al. (2010) | China | GA | Neurosurgery | 20-60 | 40 | 2/10 | PC6 | TTT | 40 | Sham-TEAS |
| Wu et al. (2016) | China | GA | Cardiothoracic surgery | 18-65 | 27 | 2/100 | LI4, PC6, SI3, SJ6 | TST | 54 | Non-/sham-TEAS |
| Wu et al. (2013) | China | GA | Neurosurgery | 48 ± 9 | 20 | 2/100 | EX-HN4, EX-HN5, LI4, SI18, GB-20 | TTS | 20 | Non-TEAS |
| Xing et al. (2012) | China | GA | Head and neck surgery | N/A | 30 | 2/100 | PC6, LI4 | TTS | 30 | Non-TEAS |
| Xiong et al. (2021) | China | GA | Abdominal surgery | < 65 | 31 | 2/10 | PC6, ST36 | TTS | 31 | Non-TEAS |
| Xu et al. (2012) | China | GA | Neurosurgery | ≥ 18 | 60 | 2/100 | PC6 | TTT | 59 | Non-TEAS |
| Yang et al. (2015) | China | GA | Abdominal surgery | 18-60 | 50 | 2 | PC6 | TTT | 50 | Non-TEAS |
| Yao et al. (2015) | China | GA | Gynecology | 18-60 | 35 | 2/10 | PC6, LI4, SP6, ST36 | TSS | 36 | Non-TEAS |
| Yeh et al. (2011) | China | GA | Orthopedic surgery | ≥ 18 | 30 | 2/100 | BL40, GB34, HT7, PC6 | SST | 60 | Non-/sham-TEAS |
| Yeh et al. (2010) | China | GA | Orthopedic surgery | N/A | 33 | 2/100 | BL40, GB34, HT7, PC6 | TST | 61 | Non-/sham-TEAS |
| Yeoh et al. (2016) | Malaysia | GA | Abdominal surgery | ≥ 18 | 40 | N/A | PC6 | SST | 40 | Non-TEAS |
| Yin et al. (2013) | China | GA | Gynecology | ≥ 18 | 30 | 2 | ST36, ST34 | TTS | 30 | Non-TEAS |
| Yu et al. (2010) | China | GA | Breast surgery | 20-70 | 30 | 2/100 | LI4, PC8, PC6, SJ5 | TTS | 30 | Non-TEAS |
| Yu et al. (2020) | China | GA | Gynecology | 29-60 | 30 | 2/100 | GV20, EX-HN3, ST36, PC6 | TSS | 30 | Non-TEAS |
| Zarate et al. (2001) | America | GA | Abdominal surgery | ≥ 18 | 110 | 31 | PC6 | SST | 111 | Non-TEAS |
| Zhan et al. (2020) | China | GA+ LA | Abdominal surgery | 18-85 | 30 | 2-100 | ST36, ST35, PC6 | TST | 30 | Non-TEAS |
| Zhang et al. (2018) | China | GA | Abdominal surgery | ≥ 18 | 21 | 25 | ST36, PC6 | SST | 21 | Sham-TEAS |
| Zhang et al. (2019) | China | GA | Abdominal surgery | 18-65 | 30 | 25, 100 | ST36, PC6 | TTT | 30 | Sham-TEAS |
| Zhang et al. (2016) | China | GA | Cardiothoracic surgery | ≥ 18 | 117 | 2/10 | LI4, CVI7; CV17, CV14 | TSS | 61 | Sham-TEAS |
| Zhang et al. (2014) | China | GA | Breast surgery | 20-50 | 33 | 2/10 | PC6, LI4, ST36 | TSS | 32 | Non-TEAS |
| Zhao et al. (2021) | China | GA | Cardiothoracic surgery | N/A | 47 | 4/20 | PC6, PC4 | TSS | 47 | Non-TEAS |
| Zhao et al. (2015) | China | GA | Otolaryngology | 20-70 | 30 | 2/100 | LI4, ST36, SP6, LI11 | TTS | 30 | Non-TEAS |
| Zhao et al. (2020) | China | GA | Cardiothoracic surgery | N/A | 63 | 100 | BL13, LI4, ST36 | TSS | 49 | Non-TEAS |
| Zhou et al. (2018) | China | LA | Gynecology | ≥ 18 | 43 | 2/10 | PC6, ST36 | TST | 89 | Non-/sham-TEAS |
| Zhou et al. (2021) | China | GA | Abdominal surgery | 18-75 | 41 | 2/100 | LI4, PC6, BL21, BL27, ST36, ST37 | SST | 40 | Non-TEAS |

*Note:* Table detailing the main relevant findings of each included study.

*Legend:* GA= general anesthesia; LA= local anesthesia; TG= treatment group, CG= control group, n= number of patients, TEAS= transcutaneous electrical acupoint stimulation, TSS= preoperative TEAS, SST= postoperative TEAS, TTS= preoperative+ intraoperative TEAS, TST= preoperative+ postoperative TEAS, TTT= full perioperative TEAS

| **Table 2. Summary of primary outcomes** | | | | | | | | |
| --- | --- | --- | --- | --- | --- | --- | --- | --- |
| **Outcomes** | **Studies included** | **Patients** | **TEAS groups**  **Mean (SD)** | **Control groups**  **Mean (SD)** | **WMD (97.5%/99% CI)** | **P-value** | ***I*^2^** | **GRADE Score** |
| Cumulative 24 hours intravenous morphine equivalent consumption (mg)^*^ | 6 | 455 | 59.39 (75.73) | 86.61 (109.93) | -14.60 (-23.60 to -5.60) | < 0.001 | 97 | ⊕⊕○○ low^1,2^ |
| Rest pain scores within 24 hours after surgery (cm) | | | | | | | | |
| At 2 hours | 6 | 401 | 2.93 (2.01) | 3.99 (2.20) | -0.96 (-1.44 to -0.48) | < 0.001 | 66 | ⊕⊕○○ low^1,2^ |
| At 6 hours | 7 | 619 | 3.00 (1.27) | 3.93 (1.85) | -0.74 (-1.83 to 0.35) | 0.08 | 98 | ⊕⊕○○ low^2,3^ |
| At 12 hours | 6 | 457 | 2.98 (1.03) | 4.14 (1.62) | -1.02 (-1.87 to -0.17) | 0.002 | 97 | ⊕⊕○○ low^1,2^ |
| At 24 hours | 21 | 3743 | 2.15 (1.33) | 3.12 (2.45) | -0.79 (-1.25 to -0.32) | < 0.001 | 98 | ⊕⊕○○ low^1,2^ |

GRADE Working Group grades of evidence:

High quality: We are very confident that the true effect lies close to that of the estimate of the effect.

Moderate quality: We are moderately confident in the effect estimate: The true effect is likely to be close to the estimate of the effect, but there is a possibility that it is substantially different.

Low quality: Our confidence in the effect estimate is limited: The true effect may be substantially different from the estimate of the effect.

Very low quality: We have very little confidence in the effect estimate: The true effect is likely to be substantially different from the estimate of effect.

CI, indicates confidence interval; WMD, weighted mean difference

^1^ 'Some concerns' in Risk of Bias
^2^ High heterogeneity (*I^2^*> 50%)

^3^ Some evidence of imprecision

^*^ 97.5% CI

| **Table 3. Summary of second outcomes** | | | | | | |
| --- | --- | --- | --- | --- | --- | --- |
| **Outcomes** | **Studies Included** | **Patients** | **WMD or RR (95%/99% CI)** | **P-value** | ***I*^2^** | **GRADE Score** |
| **Perioperative pain-related indicators** | | | | | | |
| Cumulative 48-h intravenous morphine equivalent consumption (mg) | 5 | 400 | -20.20 [-28.06, -12.33] | < 0.001 | 98 | ⊕⊕⊕○ moderate^2^ |
| 48 hours rest pain scores (cm) ^*^ | 12 | 925 | -0.57 [-0.97, -0.18] | < 0.001 | 96 | ⊕⊕○○ low^1,2^ |
| 72 hours rest pain scores (cm) ^*^ | 6 | 442 | -0.78 [-1.70, 0.14] | 0.03 | 99 | ⊕○○○ very low ^2,3,4^ |
| 24-h rescue analgesia rate | 6 | 991 | 0.53 [0.38, 0.74] | < 0.001 | 0 | ⊕⊕⊕⊕ high |
| 48-h rescue analgesia rate | 3 | 521 | 0.45 [0.29, 0.70] | < 0.001 | 0 | ⊕⊕⊕⊕ high |
| Intraoperative consumption of sedatives and anesthesia | | | | | | |
| propofol (mg) | 15 | 4069 | -28.87 [-52.25, -5.50] | 0.02 | 89 | ⊕⊕⊕○ moderate^2^ |
| fentanyl (μg) | 11 | 1023 | -20.36 [-40.01, -0.72] | 0.04 | 68 | ⊕⊕⊕○ moderate^2^ |
| remifentanil (μg) | 16 | 4085 | -128.41 [-183.28, -73.55] | < 0.001 | 98 | ⊕⊕○○ low^2,4^ |
| sufentanil (μg) | 8 | 2823 | 0.03 [-1.01, 1.07] | 0.95 | 64 | ⊕⊕○○ low^2,3^ |
| 24 hours IL-6 (pg/mL) ^*^ | 8 | 586 | -12.05 [-15.86, -8.23] | < 0.001 | 99 | ⊕⊕⊕○ moderate^2^ |
| 24 hours TNF-α (pg/ mL) ^*^ | 5 | 382 | -19.31 [-44.72, 6.11] | 0.05 | 99 | ⊕⊕○○ low^2,3^ |
| 24 hours NE (pg/mL) ^*^ | 2 | 102 | -68.37 [-200.49, 63.74] | 0.18 | 88 | ⊕○○○ very low ^2,3,4^ |
| 48 hours IL-6 (pg/mL) ^*^ | 3 | 170 | -19.48 [-45.34, 6.38] | 0.05 | 78 | ⊕⊕○○ low^2,3^ |
| 48 hours TNF-α (pg/ mL) ^*^ | 2 | 120 | -15.91 [-27.89, -3.93] | 0.009 | 93 | ⊕⊕⊕○ moderate^2^ |
| 72 hours IL-6 (pg/mL) ^*^ | 4 | 838 | -7.37 [-13.90, -0.84] | 0.004 | 98 | ⊕⊕⊕○ moderate^2^ |
| 72 hours TNF-α (pg/ mL) ^*^ | 2 | 168 | -0.35 [-1.90, 1.20] | 0.66 | 97 | ⊕⊕○○ low^2,3^ |
| **Postoperative rehabilitation-related outcomes** | | | | | | |
| 24 hours QoR-40 | 7 | 511 | 10.64 [6.14, 15.14] | < 0.001 | 89 | ⊕⊕⊕○ moderate^2^ |
| 48 hours QoR-40 | 3 | 230 | 1.91 [0.97, 2.84] | < 0.001 | 22 | ⊕⊕⊕⊕ high |
| Time to first flatus (hours) | 16 | 2223 | -11.17 [-15.35, -7.00] | < 0.001 | 95 | ⊕⊕○○ low^1,2^ |
| Time to first defecation (hours) | 12 | 1974 | -15.88 [-21.15, -10.62] | < 0.001 | 94 | ⊕⊕○○ low^1,2^ |
| Time to first feeding (hours) | 9 | 596 | -10.64 [-18.01, -3.27] | 0.005 | 97 | ⊕⊕○○ low^2,4^ |
| Time to first ambulation (hours) | 7 | 662 | -11.29 [-27.86, 5.28] | 0.18 | 98 | ⊕○○○ very low^2,3,4^ |
| Time to first bowel sounds (hours) | 4 | 895 | -4.86 [-6.25, -3.46] | < 0.001 | 26 | ⊕⊕⊕⊕ high |
| Length of hospital stay (days) | 21 | 2151 | -0.98 [-1.37, -0.59] | < 0.001 | 93 | ⊕⊕○○ low^1,2^ |
| **Postoperative opioid-related side effect** | | | | | | |
| 24 hours after surgery | | | | | | |
| PON | 15 | 1879 | 0.63 [0.53, 0.76] | < 0.001 | 55 | ⊕⊕○○ low^1,2^ |
| POV | 14 | 3383 | 0.63 [0.54, 0.73] | < 0.001 | 0 | ⊕⊕⊕○ moderate^1^ |
| PONV | 19 | 3767 | 0.67 [0.61, 0.73] | < 0.001 | 59 | ⊕⊕⊕○ moderate^2^ |
| Dizziness | 8 | 2178 | 0.56 [0.41, 0.77] | < 0.001 | 69 | ⊕⊕○○ low^2,4^ |
| Pruritus | 3 | 225 | 0.49 [0.21, 1.16] | 0.11 | 59 | ⊕⊕○○ low^2,4^ |
| 48 hours after surgery | | | | | | |
| PON | 2 | 421 | 0.71 [0.21, 2.40] | 0.58 | 84 | ⊕⊕○○ low^2,4^ |
| POV | 3 | 483 | 0.65 [0.30, 1.40] | 0.27 | 77 | ⊕⊕⊕○ moderate^2^ |
| PONV | 4 | 267 | 0.47 [0.33, 0.66] | < 0.001 | 0 | ⊕⊕⊕⊕ high |
| Dizziness | 3 | 185 | 0.64 [0.39, 1.04] | 0.07 | 7 | ⊕⊕⊕○ moderate^4^ |
| Pruritus | 3 | 185 | 0.67 [0.32, 1.41] | 0.29 | 0 | ⊕⊕⊕○ moderate^4^ |
| 24-h rescue antiemetic rate | 9 | 788 | 0.66 [0.53, 0.82] | < 0.001 | 30 | ⊕⊕⊕○ moderate^1^ |
| 48-h rescue antiemetic rate | 2 | 423 | 0.79 [0.35, 1.78] | 0.56 | 79 | ⊕⊕○○ low^2,4^ |

High quality: We are very confident that the true effect lies close to that of the estimate of the effect.

Moderate quality: We are moderately confident in the effect estimate: The true effect is likely to be close to the estimate of the effect, but there is a possibility that it is substantially different.

Low quality: Our confidence in the effect estimate is limited: The true effect may be substantially different from the estimate of the effect.

Very low quality: We have very little confidence in the effect estimate: The true effect is likely to be substantially different from the estimate of effect.

QoR-40, quality of recovery-40; IL, interleukin; TNF-α, tumor necrosis factor-α; NE, epinephrine; PON, postoperative nausea; POV, postoperative vomiting; PONV, postoperative nausea and vomiting.

CI, indicates confidence interval; WMD, weighted mean difference; RR, risk ratio.

^1^ 'Some concerns' in Risk of Bias
^2^ High heterogeneity (*I^2^* > 50%)

^3^ Some evidence of imprecision

^4^ Having evidence of publication bias

^*^ 99% CI
