# Supplementary material for: Effects of transcutaneous electrical acupoint stimulation on early postoperative pain and recovery: a comprehensive systematic review and meta-analysis of randomized controlled trials
Source: Front Med (Lausanne). 2024 Apr 29;11:1302057. doi: 10.3389/fmed.2024.1302057 (PMC11092893; doi:10.3389/fmed.2024.1302057)
Supplement: Supplementary file 1 [file Data_Sheet_1.ZIP › Supplementary material/Supplementary material 2.docx]

Supplementary Material

**Effects of transcutaneous electrical acupoint stimulation on early postoperative pain and recovery: A comprehensive systematic review and meta-analysis of randomized controlled trials**

**Shi-Yan Tan ^1†^, Hua Jiang ^1†^, Qiong Ma ^1†^, Xin Ye^1^, Xi Fu^1^, Yi-Feng Ren^1*^, Feng-Ming You^1*^**

*** Correspondence:** Feng-Ming You: [yfmdoc@163.com](mailto:yfmdoc@163.com); Yi-Feng Ren: [ryftcm.dr@yahoo.com](mailto:ryftcm.dr@yahoo.com)

| **Table of Contents** | |
| --- | --- |
| **eMethods 1:** PubMed Search Strategy | **Page 2-5** |
| **eMethods 2:** Web of Science Search Strategy | **Page 5** |
| **eMethods 3:** EMBASE Search Strategy | **Page 6** |
| **eMethods 4:** Cochrane Library Search Strategy | **Page 6** |
| **eMethods 5:** Google Scholar Search Strategy | **Page 6** |
| **eReferences:** Included studies | **Page 7-12** |
| **eTable 1:** Risk of Bias Assessment | **Page 13-57** |
| **eFigure 1:** Cross Tabulation Risk of Bias | **Page 58** |
| **eTable 2:** GRADE quality of evidence summary table | **Page 59-66** |
| **eTable 3:** Predefined sensitivity analyses for primary outcomes | **Page 67** |
| **eTable 4:** Subgroup analyses for primary outcomes | **Page 68-72** |
| **eTable 5:** Meta-regression analysis for primary outcomes | **Page 73-74** |

**eMethods 1: PubMed Search Strategy (374)**

| **Subject words:** | **Free words** |
| --- | --- |
| - | transcutaneous acupoint electrical stimulation  transcutaneous electrical acupoint stimulation  transcutaneous point electric stimulation  transcutaneous electrical stimulation of acupuncture points  transcutanclus electrical acupoint stimulation  transcutaneous electric nerve stimulation  Transcutaneous Electrical Acupoint Stimulation  transcutaneous electric stimulation on acupoints  Transcutaneous Electrical Point Stimulation  Transcutaneous Acupoint Electrical Stimulation  Percutaneous Electrical Stimulation of Acupuncture Points  Transcutaneous Electro-acupuncture  transcutaneous electrical stimulation of acupuncture points  Han's Acupiont Nerve Stimulation  Transcutaneous Acupoint Electrioacupuncture  Transcutaneous Electro-Acupuncture  TRANSCUTANEOUS ELECTRICAL ACUPOINT STIMULATION  Acupoint Transcutaneus Electrical Nerve Stimulation  ATENS  Acupoint TENS  TEPS  TAES  TEAS  TENS  Teas |
| Perioperative Period | Period, Perioperative  Periods, Perioperative  Perioperative Periods  Perioperative  Perioperation  Round operation period  Peri-operative  the Perioperative period |
| General Surgery  Surgical Procedures, Operative | Surgery, General  Surgery  Operative Procedures  Operative Procedure  Procedure, Operative  Procedures, Operative  Surgical Procedure, Operative  Operative Surgical Procedures  Procedure, Operative Surgical  Procedures, Operative Surgical  Surgical Procedures  Procedure, Surgical  Procedures, Surgical  Surgical Procedure  Operative Surgical Procedure  Surgery, Ghost  Ghost Surgery |
| Preoperative Period | Period, Preoperative |
| Postoperative Period | Period, Postoperative  Periods, Postoperative  Postoperative Periods  Post-surgery |
| - | Randomized controlled trial  randomized  placebo |

1.((((((((((((((((((((((((transcutaneous acupoint electrical stimulation[Title/Abstract]) OR (transcutaneous electrical acupoint stimulation[Title/Abstract])) OR (transcutaneous point electric stimulation[Title/Abstract])) OR (transcutaneous electrical stimulation of acupuncture points[Title/Abstract])) OR (transcutanclus electrical acupoint stimulation[Title/Abstract])) OR (transcutaneous electric nerve stimulation[Title/Abstract])) OR (Transcutaneous Electrical Acupoint Stimulation[Title/Abstract])) OR (transcutaneous electric stimulation on acupoints[Title/Abstract])) OR (Transcutaneous Electrical Point Stimulation[Title/Abstract])) OR (Transcutaneous Acupoint Electrical Stimulation[Title/Abstract])) OR (Percutaneous Electrical Stimulation of Acupuncture Points[Title/Abstract])) OR (Transcutaneous Electro-acupuncture[Title/Abstract])) OR (transcutaneous electrical stimulation of acupuncture points[Title/Abstract])) OR (Han's Acupiont Nerve Stimulation[Title/Abstract])) OR (Transcutaneous Acupoint Electroacupuncture[Title/Abstract])) OR (Transcutaneous Electro-Acupuncture[Title/Abstract])) OR (TRANSCUTANEOUS ELECTRICAL ACUPOINT STIMULATION[Title/Abstract])) OR (Acupoint Transcutaneus Electrical Nerve Stimulation[Title/Abstract])) OR (ATENS[Title/Abstract])) OR (Acupoint TENS[Title/Abstract])) OR (TEPS[Title/Abstract])) OR (TAES[Title/Abstract])) OR (TEAS[Title/Abstract])) OR (TENS[Title/Abstract])) OR (Teas[Title/Abstract]) **(22,607)**

2."Perioperative Period"[Mesh] **(101,273)**

3.(((((((Period, Perioperative[Title/Abstract]) OR (Periods, Perioperative[Title/Abstract])) OR (Perioperative Periods[Title/Abstract])) OR (Perioperative[Title/Abstract])) OR (Perioperation[Title/Abstract])) OR (Round operation period[Title/Abstract])) OR (Peri-operative[Title/Abstract])) OR (the Perioperative period[Title/Abstract]) **(119,542)**

4."General Surgery"[Mesh] **(40,330)**

5."Surgical Procedures, Operative"[Mesh] **(3,437,485)**

6.(((((((((((((((((operation[Title/Abstract]) OR (Surgery, General[Title/Abstract])) OR (Surgery[Title/Abstract])) OR (Operative Procedures[Title/Abstract])) OR (Operative Procedure[Title/Abstract])) OR (Procedure, Operative[Title/Abstract])) OR (Procedures, Operative[Title/Abstract])) OR (Surgical Procedure, Operative[Title/Abstract])) OR (Operative Surgical Procedures[Title/Abstract])) OR (Procedure, Operative Surgical[Title/Abstract])) OR (Procedures, Operative Surgical[Title/Abstract])) OR (Surgical Procedures[Title/Abstract])) OR (Procedure, Surgical[Title/Abstract])) OR (Procedures, Surgical[Title/Abstract])) OR (Surgical Procedure[Title/Abstract])) OR (Operative Surgical Procedure[Title/Abstract])) OR (Ghost Surgery[Title/Abstract])) OR (Surgery, Ghost[Title/Abstract]) **(1,725,150)**

7."Preoperative Period"[Mesh] **(9,409)**

8.Period, Preoperative [Title/Abstract] **(242)**

9."Postoperative Period"[Mesh] **(61,023)**

10.(((Period, Postoperative [Title/Abstract]) OR (Periods, Postoperative [Title/Abstract])) OR (Postoperative Periods [Title/Abstract])) OR (Post-surgery [Title/Abstract]) **(14,679)**

11.((Randomized controlled trial [Publication Type]) OR (randomized [Title/Abstract])) OR (placebo[Title/Abstract]) **(957,930)**

12.(((((((("Perioperative Period"[Mesh]) OR ((((((((Period, Perioperative[Title/Abstract]) OR (Periods, Perioperative[Title/Abstract])) OR (Perioperative Periods[Title/Abstract])) OR (Perioperative[Title/Abstract])) OR (Perioperation[Title/Abstract])) OR (Round operation period[Title/Abstract])) OR (Peri-operative[Title/Abstract])) OR (the Perioperative period[Title/Abstract]))) OR ("General Surgery"[Mesh])) OR ("Surgical Procedures, Operative"[Mesh])) OR (((((((((((((((((Surgery, General[Title/Abstract]) OR (Surgery[Title/Abstract])) OR (Operative Procedures[Title/Abstract])) OR (Operative Procedure[Title/Abstract])) OR (Procedure, Operative[Title/Abstract])) OR (Procedures, Operative[Title/Abstract])) OR (Surgical Procedure, Operative[Title/Abstract])) OR (Operative Surgical Procedures[Title/Abstract])) OR (Procedure, Operative Surgical[Title/Abstract])) OR (Procedures, Operative Surgical[Title/Abstract])) OR (Surgical Procedures[Title/Abstract])) OR (Procedure, Surgical[Title/Abstract])) OR (Procedures, Surgical[Title/Abstract])) OR (Surgical Procedure[Title/Abstract])) OR (Operative Surgical Procedure[Title/Abstract])) OR (Surgery, Ghost[Title/Abstract])) OR (Ghost Surgery[Title/Abstract]))) OR ("Preoperative Period"[Mesh])) OR (Period, Preoperative[Title/Abstract])) OR ("Postoperative Period"[Mesh])) OR ((((Period, Postoperative[Title/Abstract]) OR (Periods, Postoperative[Title/Abstract])) OR (Postoperative Periods[Title/Abstract])) OR (Post-surgery[Title/Abstract])) **(4,122,799)**

13.((((((((((((((((((((((((((transcutaneous acupoint electrical stimulation[Title/Abstract]) OR (transcutaneous electrical acupoint stimulation[Title/Abstract])) OR (transcutaneous point electric stimulation[Title/Abstract])) OR (transcutaneous electrical stimulation of acupuncture points[Title/Abstract])) OR (transcutanclus electrical acupoint stimulation[Title/Abstract])) OR (transcutaneous electric nerve stimulation[Title/Abstract])) OR (Transcutaneous Electrical Acupoint Stimulation[Title/Abstract])) OR (transcutaneous electric stimulation on acupoints[Title/Abstract])) OR (Transcutaneous Electrical Point Stimulation[Title/Abstract])) OR (Transcutaneous Acupoint Electrical Stimulation[Title/Abstract])) OR (Percutaneous Electrical Stimulation of Acupuncture Points[Title/Abstract])) OR (Transcutaneous Electro-acupuncture[Title/Abstract])) OR (transcutaneous electrical stimulation of acupuncture points[Title/Abstract])) OR (Han's Acupiont Nerve Stimulation[Title/Abstract])) OR (Transcutaneous Acupoint Electrioacupuncture[Title/Abstract])) OR (Transcutaneous Electro-Acupuncture[Title/Abstract])) OR (TRANSCUTANEOUS ELECTRICAL ACUPOINT STIMULATION[Title/Abstract])) OR (Acupoint Transcutaneus Electrical Nerve Stimulation[Title/Abstract])) OR (ATENS[Title/Abstract])) OR (Acupoint TENS[Title/Abstract])) OR (TEPS[Title/Abstract])) OR (TAES[Title/Abstract])) OR (TEAS[Title/Abstract])) OR (TENS[Title/Abstract])) OR (Teas[Title/Abstract])) AND ((((((((("Perioperative Period"[Mesh]) OR ((((((((Period, Perioperative[Title/Abstract]) OR (Periods, Perioperative[Title/Abstract])) OR (Perioperative Periods[Title/Abstract])) OR (Perioperative[Title/Abstract])) OR (Perioperation[Title/Abstract])) OR (Round operation period[Title/Abstract])) OR (Peri-operative[Title/Abstract])) OR (the Perioperative period[Title/Abstract]))) OR ("General Surgery"[Mesh])) OR ("Surgical Procedures, Operative"[Mesh])) OR (((((((((((((((((Surgery, General[Title/Abstract]) OR (Surgery[Title/Abstract])) OR (Operative Procedures[Title/Abstract])) OR (Operative Procedure[Title/Abstract])) OR (Procedure, Operative[Title/Abstract])) OR (Procedures, Operative[Title/Abstract])) OR (Surgical Procedure, Operative[Title/Abstract])) OR (Operative Surgical Procedures[Title/Abstract])) OR (Procedure, Operative Surgical[Title/Abstract])) OR (Procedures, Operative Surgical[Title/Abstract])) OR (Surgical Procedures[Title/Abstract])) OR (Procedure, Surgical[Title/Abstract])) OR (Procedures, Surgical[Title/Abstract])) OR (Surgical Procedure[Title/Abstract])) OR (Operative Surgical Procedure[Title/Abstract])) OR (Surgery, Ghost[Title/Abstract])) OR (Ghost Surgery[Title/Abstract]))) OR ("Preoperative Period"[Mesh])) OR (Period, Preoperative[Title/Abstract])) OR ("Postoperative Period"[Mesh])) OR ((((Period, Postoperative[Title/Abstract]) OR (Periods, Postoperative[Title/Abstract])) OR (Postoperative Periods[Title/Abstract])) OR (Post-surgery[Title/Abstract])))) AND ("randomized controlled trial"[Publication Type] OR "randomized"[Title/Abstract] OR "placebo"[Title/Abstract]) **(374)**

**eMethods 2: Web of Science Search Strategy (450)**


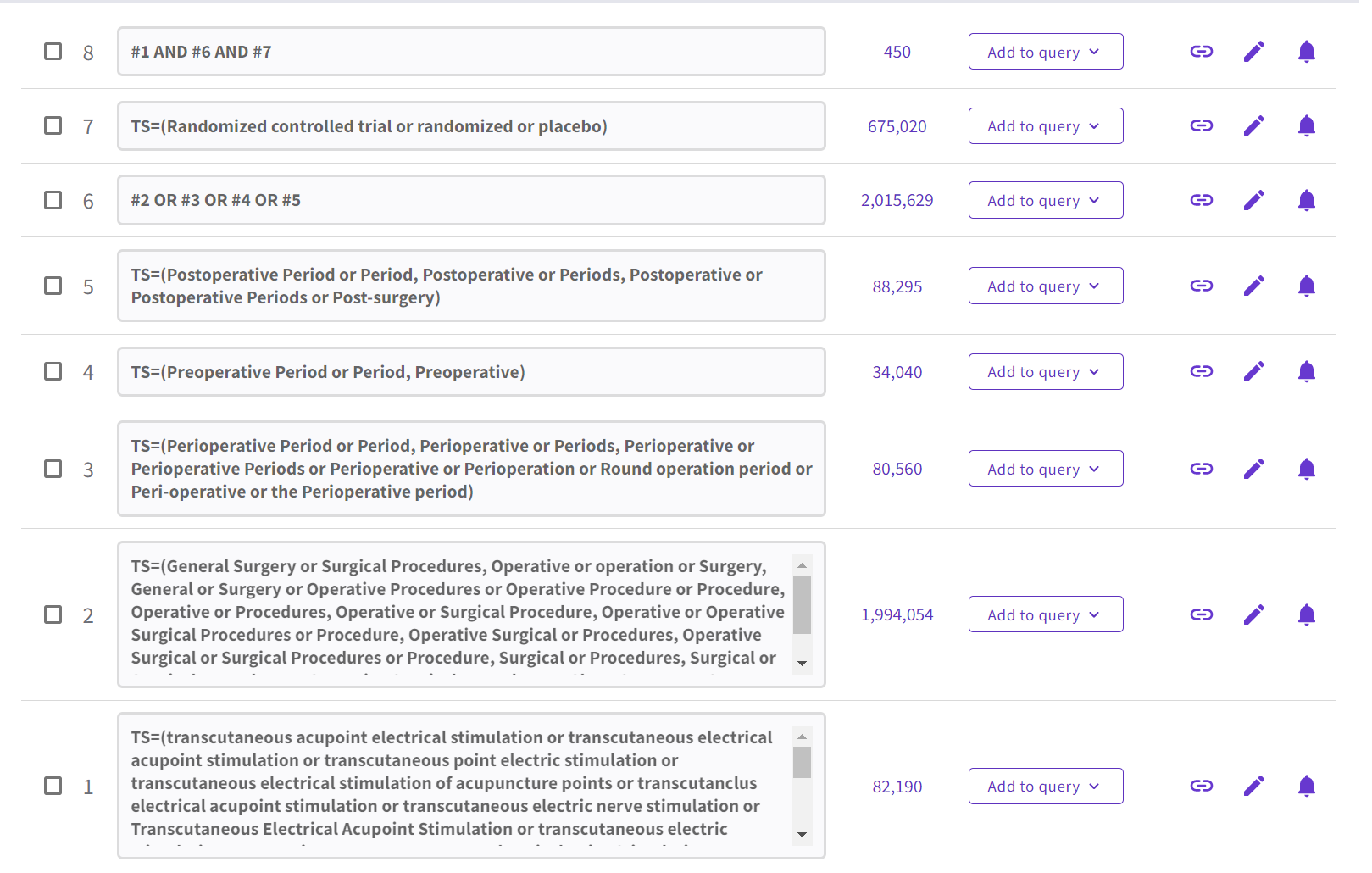


**eMethods 3: EMBASE Search Strategy (403)**


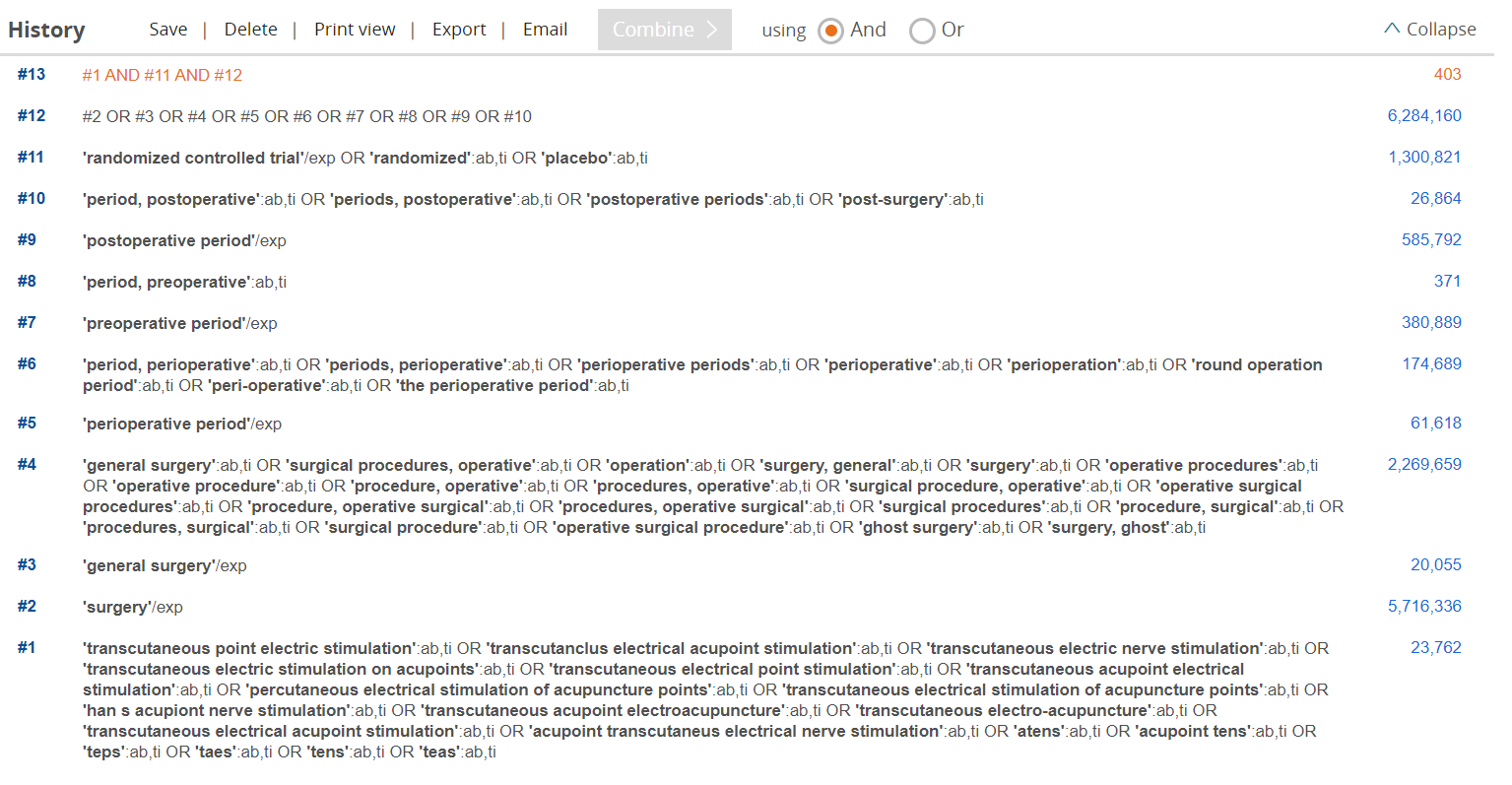


**eMethods 4: Cochrane Library Search Strategy (698)**


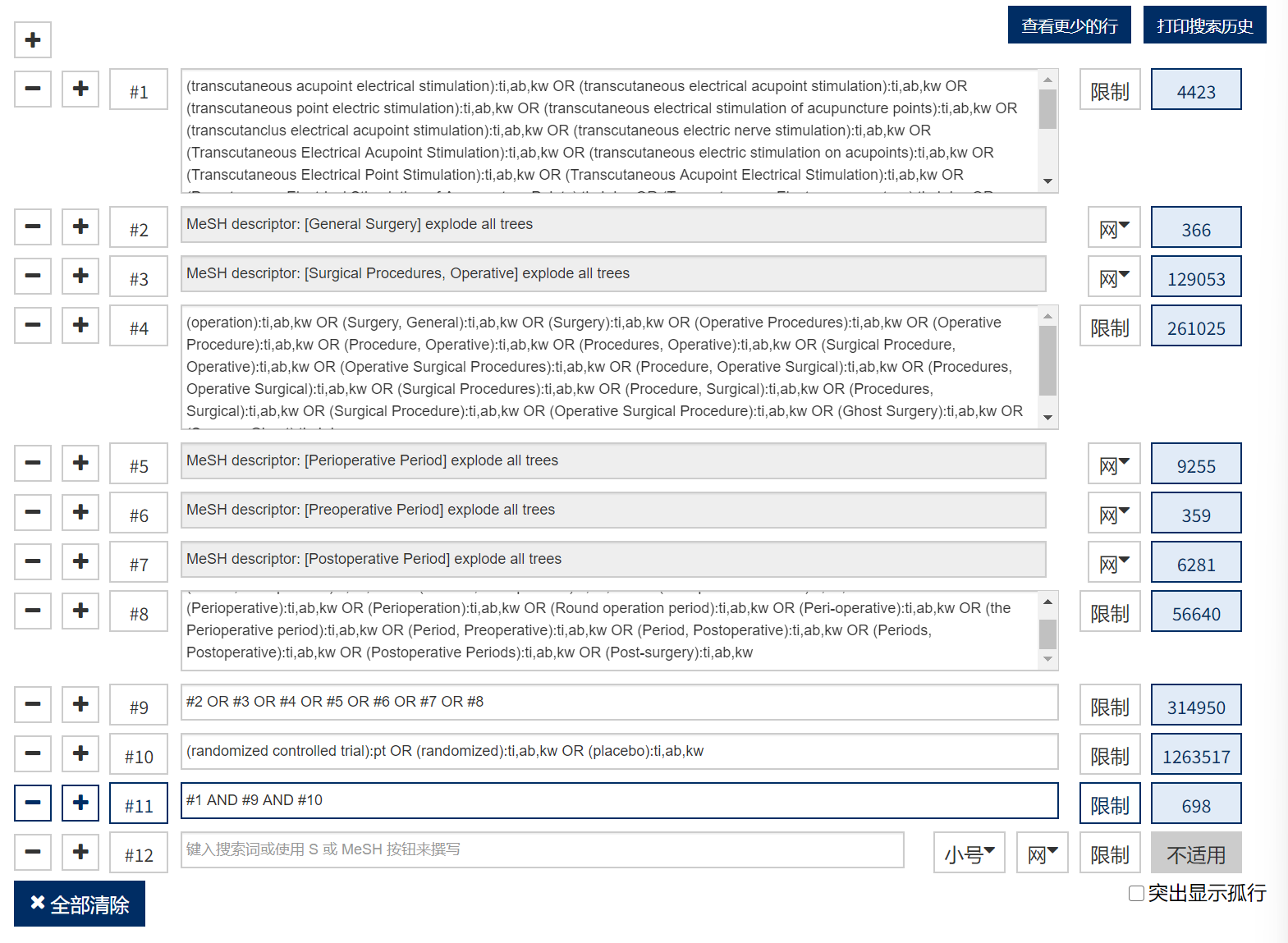


**eMethods 5: Google Scholar Search Strategy (100)**

(transcutaneous acupoint electrical stimulation) AND (operation or surgery) AND clinical trial (First ten pages of results (100 citations) included

**eReferences: Included studies**

1. Ao L, Shi J, Bai Y, et al. Effects of transcutaneous electrical acupoint stimulation on perioperative immune function and postoperative analgesia in patients undergoing radical mastectomy: a randomized controlled trial. Experimental and therapeutic medicine 2021;21(3):184, doi:10.3892/etm.2021.9615

2. Arnberger M, Stadelmann K, Alischer P, et al. Monitoring of neuromuscular blockade at the P6 acupuncture point reduces the incidence of postoperative nausea and vomiting. Anesthesiology 2007;107(6):903-8, doi:10.1097/01.anes.0000290617.98058.d9

3. Bai WY, Yang YC, Teng XF, et al. Effects of Transcutaneous Electrical Acupoint Stimulation on the Stress Response During Extubation After General Anesthesia in Elderly Patients Undergoing Elective Supratentorial Craniotomy: A Prospective Randomized Controlled Trial. Journal of Neurosurgical Anesthesiology 2018;30(4):337-346, doi:10.1097/ana.0000000000000460

4. Chen J, Zhang Y, Li X, et al. Efficacy of transcutaneous electrical acupoint stimulation combined with general anesthesia for sedation and postoperative analgesia in minimally invasive lung cancer surgery: a randomized, double-blind, placebo-controlled trial. Thoracic cancer 2020;11(4):928‐934, doi:10.1111/1759-7714.13343

5. Chen K-B, Lu Y-Q, Chen J-D, et al. Transcutaneous electroacupuncture alleviates postoperative ileus after gastrectomy: A randomized clinical trial. World Journal of Gastrointestinal Surgery 2018;10(2):13-20, doi:10.4240/wjgs.v10.i2.13

6. Chen L, Tang J, White PF, et al. The Effect of Location of Transcutaneous Electrical Nerve Stimulation on Postoperative Opioid Analgesic Requirement: Acupoint Versus Nonacupoint Stimulation. Anesthesia & Analgesia 1998;87(5):1129-34

7. Chen X, Wang BG, Li J, et al. Effect of transcutaneous acupoint electric stimulation on perioperative intravenous anesthesia in patients of transsphenoidal pituitary tumor resection. Zhongguo zhen jiu [Chinese acupuncture & moxibustion] 2013;33(8):732‐736

8. Chen Y, Yang Y, Yao Y, et al. Does transcutaneous electric acupoint stimulation improve the quality of recovery after thyroidectomy? A prospective randomized controlled trial. International journal of clinical and experimental medicine 2015;8(8):13622‐13627

9. Chen Y, Yao Y, Wu Y, et al. Transcutaneous electric acupoint stimulation alleviates remifentanil-induced hyperalgesia in patients undergoing thyroidectomy: a randomized controlled trial. International journal of clinical and experimental medicine 2015;8(4):5781‐5787

10. Chi YL, Zhang WL, Yang F, et al. Transcutaneous Electrical Acupoint Stimulation for Improving Postoperative Recovery, Reducing Stress and Inflammatory Responses in Elderly Patient Undergoing Knee Surgery. American Journal of Chinese Medicine 2019;47(7):1445-1458, doi:10.1142/s0192415x19500745

11. Chiu JH, Chen WS, Chen CH, et al. Effect of transcutaneous electrical nerve stimulation for pain relief on patients undergoing hemorrhoidectomy: prospective, randomized, controlled trial. Diseases of the colon and rectum 1999;42(2):180‐185, doi:10.1007/BF02237124

12. Ertas G, Bengi Sener E, Kaya C, et al. Effects of P6 acustimulation with the ReliefBand on postoperative nausea and vomiting in patients undergoing gynecological laparoscopy. Holistic nursing practice 2015;29(1):6‐12, doi:10.1097/HNP.0000000000000061

13. Gan TJ, Jiao KR, Zenn M, et al. A randomized controlled comparison of electro-acupoint stimulation or ondansetron versus placebo for the prevention of postoperative nausea and vomiting. Anesthesia & Analgesia 2004;99(4):1070-1075, doi:10.1213/01.ANE.0000130355.91214.9E

14. Gao F, Zhang Q, Li Y, et al. Transcutaneous electrical acupoint stimulation for prevention of postoperative delirium in geriatric patients with silent lacunar infarction: a preliminary study. Clinical interventions in aging 2018;13:2127‐2134, doi:10.2147/CIA.S183698

15. Gao P, Shao B, Diao YG, et al. Effect of transcutaneous electrical acupoint stimulation on catheter related bladder discomfort after ureteroscopic lithotripsy. Zhongguo zhen jiu [Chinese acupuncture & moxibustion] 2020;40(8):829‐833, doi:10.13703/j.0255-2930.20190729-k0001

16. Gao W, Li W, Yan Y, et al. Transcutaneous electrical acupoint stimulation applied in lower limbs decreases the incidence of paralytic ileus after colorectal surgery: A multicenter randomized controlled trial. Surgery (United States) 2021;170(6):1618-1626, doi:10.1016/j.surg.2021.08.007

17. Gao W, Zhang L, Han X, et al. Transcutaneous Electrical Acupoint Stimulation Decreases the Incidence of Postoperative Nausea and Vomiting After Laparoscopic Non-gastrointestinal Surgery: A Multi-Center Randomized Controlled Trial. Frontiers in Medicine 2022;9:766244, doi:10.3389/fmed.2022.766244

18. Gao Y-Q, Jia Q, Xie S, et al. Clinical Trials for Thyroidectomy Under Acupuncture-aided Anesthesia by Using Electroacupuncture or Transcutaneous Acupoint Electrical Stimulation of Different Acupoints. Zhen ci yan jiu = Acupuncture research 2017;42(4):332-337

19. Ge M, Zhai XJ, Li Y. Clinical efficacy of adductor canal blockade combined with transcutaneous electrical acupoint stimulation for total knee arthroplasty. Zhongguo gu shang [China journal of orthopaedics and traumatology] 2021;34(8):750‐754, doi:10.12200/j.issn.1003-0034.2021.08.011

20. Gu S, Lang H, Gan J, et al. Effect of transcutaneous electrical acupoint stimulation on gastrointestinal function recovery after laparoscopic radical gastrectomy - A randomized controlled trial. European Journal of Integrative Medicine 2019;26:11-17, doi:10.1016/j.eujim.2019.01.001

21. Guo J, Tang W, Guo F, et al. Transcutaneous electrical acupoint stimulation on inflammatory response and intestinal permeability in perioperative period of laparoscopic intestinal surgery. Zhongguo zhen jiu [Chinese acupuncture & moxibustion] 2018;38(10):1043‐1046, doi:10.13703/j.0255-2930.2018.10.004

22. Habib AS, Itchon-Ramos N, Phillips-Bute BG, et al. Transcutaneous acupoint electrical stimulation with the ReliefBand® for the prevention of nausea and vomiting during and after cesarean delivery under spinal anesthesia. Anesthesia and Analgesia 2006;102(2):581-584, doi:10.1213/01.ane.0000189217.19600.5c

23. He BM, Yang B. Analgesic effect of transcutaneous electrical acupoint stimulation combined with target-controlled infusion in general anesthesia and effects on cardiovascular system. Zhongguo zhen jiu [Chinese acupuncture & moxibustion] 2008;28(3):219‐221

24. Huang L, Pan Y, Chen S, et al. Prevention of propofol injection-related pain using pretreatment transcutaneous electrical acupoint stimulation. Turk J Med Sci 2017;47(4):1267-1276, doi:10.3906/sag-1611-35

25. Huang S, Peng W, Tian X, et al. Effects of transcutaneous electrical acupoint stimulation at different frequencies on perioperative anesthetic dosage, recovery, complications, and prognosis in video-assisted thoracic surgical lobectomy: a randomized, double-blinded, placebo-controlled trial. Journal of anesthesia 2017;31(1):58‐65, doi:10.1007/s00540-015-2057-1

26. Huang W, Long W, Xiao J, et al. Effect of electrically stimulating acupoint, Zusanli (ST 36), on patient's recovery after laparoscopic colorectal cancer resection: a randomized controlled trial. J Tradit Chin Med 2019;39(3):433-439

27. Huang W, Yu TY, Long WF, et al. Application of Transcutaneous Electrical Acupoint Stimulation Combined with Transversus Abdominis Plane Block to Enhanced Recovery After Surgery in Patients Undergoing Laparoscopic Colorectal Cancer Resection: a Randomized Controlled Clinical Trial. Zhen CI yan jiu = acupuncture research 2018;43(10):611‐615, doi:10.13702/j.1000-0607.180005

28. Jin D, Pan Y, Jin W, et al. Clinical Study on the Combination of Transcutaneous Electrical Acupoint Stimulation and Lidocaine for Preventing Propofol Injection Pain. Journal of pain research 2022;15:745‐755, doi:10.2147/JPR.S356150

29. Lan F, Ma YH, Xue JX, et al. Transcutaneous electrical nerve stimulation on acupoints reduces fentanyl requirement for postoperative pain relief after total hip arthroplasty in elderly patients. Minerva anestesiologica 2012;78(8):887‐895

30. Li JJ, Zhao WS, Shao XM, et al. Effect of Transcutaneous Electrical Acupoint Stimulation on Post-surgical Gastrointestinal Function, Autonomic Nerve Activities and Plasma Brain-gut Peptide Levels in Patients Undergoing Gastrointestinal Surgery. Zhen CI yan jiu = acupuncture research 2016;41(3):240‐246

31. Li JL, Wang XJ, Rong JF. Transcutaneous electrical acupoint stimulation relieves post-operative nausea and vomiting possibly by reducing serum motilin secretion in patients undergoing laparoscopic surgery. Zhen CI yan jiu = acupuncture research 2020;45(11):920‐923, doi:10.13702/j.1000-0607.200060

32. Li M, Xu F, Liu M, et al. Effects and Mechanisms of Transcutaneous Electrical Acustimulation on Postoperative Recovery After Elective Cesarean Section. Neuromodulation 2020;23(6):838-846, doi:10.1111/ner.13178

33. Li WJ, Gao C, An LX, et al. Perioperative transcutaneous electrical acupoint stimulation for improving postoperative gastrointestinal function: a randomized controlled trial. Journal of integrative medicine 2021;19(3):211‐218, doi:10.1016/j.joim.2021.01.005

34. Liang D, Jin S, Huang L, et al. The Effect of Transcutaneous Electrical Acupoint Stimulation on Postoperative Catheter-Related Bladder Discomfort in Patients Undergoing Transurethral Resection of the Prostate. Evidence-based complementary and alternative medicine 2021;2021: 6691459, doi:10.1155/2021/6691459

35. Liu T, Yin C, Li Y, et al. Effects of Transcutaneous Electrical Acupoint Stimulation on Postoperative Cognitive Decline in Elderly Patients: a Pilot Study. Clinical interventions in aging 2021;16:757‐765, doi:10.2147/CIA.S309082

36. Liu X, Li S, Wang B, et al. Intraoperative and postoperative anaesthetic and analgesic effect of multipoint transcutaneous electrical acupuncture stimulation combined with sufentanil anaesthesia in patients undergoing supratentorial craniotomy. Acupuncture in medicine 2015;33(4):270‐276, doi:10.1136/acupmed-2014-010749

37. Liu YY, Duan SE, Cai MX, et al. Evaluation of transcutaneous electroacupoint stimulation with the train-of-four mode for preventing nausea and vomiting after laparoscopic cholecystectomy. Chinese journal of integrative medicine 2008;14(2):94‐97, doi:10.1007/s11655-008-0094-4

38. Lu Z, Wang Q, Sun X, et al. Transcutaneous electrical acupoint stimulation before surgery reduces chronic pain after mastectomy: A randomized clinical trial. Journal of Clinical Anesthesia 2021;74(110453, doi:https://doi.org/10.1016/j.jclinane.2021.110453

39. Mi Z, Gao J, Chen X, et al. Effects of transcutaneous electrical acupoint stimulation on quality of recovery during early period after laparoscopic cholecystectomy. Zhongguo zhen jiu [Chinese acupuncture & moxibustion] 2018;38(3):256‐260, doi:10.13703/j.0255-2930.2018.03.007

40. Mu L, Gao H, Zhao ML, et al. Effect of transcutaneous electrical acupoint stimulation on recovery of gastrointestinal function after cesarean section. Zhongguo zhen jiu [Chinese acupuncture & moxibustion] 2019;39(3):259‐262, doi:10.13703/j.0255-2930.2019.03.010

41. Oztas B, Iyigun E. The effects of two different electrical stimulation methods on the pain intensity of the patients who had undergone abdominal surgery with a midline incision: randomized controlled clinical trial. Contemporary nurse 2019;55(2‐3):122‐138, doi:10.1080/10376178.2019.1628650

42. Que B, Tu Q, Shi J, et al. Effects of Transcutaneous Electrical Acupoint Stimulation on Systemic Inflammatory Response Syndrome of Patients after Percutaneous Nephrolithotomy: a Randomized Controlled Trial. Evidence-based complementary and alternative medicine 2021;2021:5909956, doi:10.1155/2021/5909956

43. Si JL, Xu L, Li GC, et al. Adjuvant effect of transcutaneous electrical acupoint stimulation in propofol-fentanyl anesthesia in partial mastectomy. Nan fang yi ke da xue xue bao [Journal of Southern Medical University] 2009;29(10):2064‐2066

44. Song B, Chang Y, Li Y, et al. Effects of Transcutaneous Electrical Acupoint Stimulation on the Postoperative Sleep Quality and Pain of Patients After Video-Assisted Thoracoscopic Surgery: A Prospective, Randomized Controlled Trial. Nat Sci Sleep 2020;12(809-819, doi:10.2147/nss.S270739

45. Sun K, Xing T, Zhang F, et al. Perioperative Transcutaneous Electrical Acupoint Stimulation for Postoperative Pain Relief Following Laparoscopic Surgery: a Randomized Controlled Trial. Clinical journal of pain 2017;33(4):340‐347, doi:10.1097/AJP.0000000000000400

46. Szmit M, Agrawal S, Goździk W, et al. Transcutaneous Electrical Acupoint Stimulation Reduces Postoperative Analgesic Requirement in Patients Undergoing Inguinal Hernia Repair: A Randomized, Placebo-Controlled Study. Journal of Clinical Medicine 2021;10(1):146, doi:10.3390/jcm10010146

47. Tu Q, Yang Z, Gan J, et al. Transcutaneous Electrical Acupoint Stimulation Improves Immunological Function During the Perioperative Period in Patients With Non-Small Cell Lung Cancer Undergoing Video-Assisted Thoracic Surgical Lobectomy. Technology in cancer research & treatment 2018;17:1533033818806477, doi:10.1177/1533033818806477

48. Wang B, Tang J, White PF, et al. Effect of the Intensity of Transcutaneous Acupoint Electrical Stimulation on the Postoperative Analgesic Requirement. Anesthesia & Analgesia 1997;85(2):406-13.

49. Wang D, Ma T, Li G, et al. Effects of transcutaneous electrical acupoint stimulation on treatment and prevention of postoperative sore throat after tracheal intubation under general anesthesia. Zhongguo zhen jiu [Chinese acupuncture & moxibustion] 2017;37(7):701‐704, doi:10.13703/j.0255-2930.2017.07.005

50. Wang H, Xie Y, Zhang Q, et al. Transcutaneous electric acupoint stimulation reduces intra-operative remifentanil consumption and alleviates postoperative side-effects in patients undergoing sinusotomy: a prospective, randomized, placebo-controlled trial. British journal of anaesthesia 2014;112(6):1075‐1082, doi:10.1093/bja/aeu001

51. Wang JL, Ren QS, Shen CC, et al. Effect of transcutaneous acupoint electrical stimulation on blood bioactive compounds involving cerebral injury during craniotomy. Zhen CI yan jiu = acupuncture research 2008;33(1):26‐30

52. Wang XQ, Yu JL, Du ZY, et al. Electroacupoint stimulation for postoperative nausea and vomiting in patients undergoing supratentorial craniotomy. Journal of neurosurgical anesthesiology 2010;22(2):128‐131, doi:10.1097/ANA.0b013e3181c9fbde

53. Wu H, Wang K, Li G, et al. Effects of transcutaneous acupoint electrical stimulation on the imbalance of Th1, Th2, Th17 and Treg cells following thoracotomy of patients with lung cancer. Exp Ther Med 2016;11(2):495-502, doi:10.3892/etm.2015.2913

54. Wu Q, Zhang MX, Wang LL, et al. Effect of transcutaneous acupoint electrical stimulation combined with target controlled infusion of propofol on efficacy of general anesthesia for craniotomy. Zhen CI yan jiu = acupuncture research 2013;38(3):229‐233

55. Xin ZQ, Ren YG, Tao MZ, et al. Study on the value of transcutaneous acupoint electrical stimulation with general intravenous anesthesia in subtotal thyroidectomy surgery. Modern Journal of Integrated Traditional Chinese and Western Medicine 2012;21(19):2065-2067

56. Xiong Q, Min S, Wei K, et al. Transcutaneous Electrical Acupoint Stimulation Combined with Dexamethasone and Tropisetron Prevents Postoperative Nausea and Vomiting in Female Patients Undergoing Laparoscopic Sleeve Gastrectomy: a Prospective, Randomized Controlled Trial. Obes Surg 2021;31(5):1912-1920, doi:10.1007/s11695-020-05205-9

57. Xu M, Zhou S-J, Jiang C-C, et al. The Effects of P6 Electrical Acustimulation on Postoperative Nausea and Vomiting in Patients After Infratentorial Craniotomy. Journal of Neurosurgical Anesthesiology 2012;24(4):312-316, doi:10.1097/ANA.0b013e31825eb5ef

58. Yang XY, Xiao J, Chen YH, et al. Dexamethasone alone vs in combination with transcutaneous electrical acupoint stimulation or tropisetron for prevention of postoperative nausea and vomiting in gynaecological patients undergoing laparoscopic surgery. British journal of anaesthesia 2015;115(6):883‐889, doi:10.1093/bja/aev352

59. Yao Y, Zhao Q, Gong C, et al. Transcutaneous Electrical Acupoint Stimulation Improves the Postoperative Quality of Recovery and Analgesia after Gynecological Laparoscopic Surgery: A Randomized Controlled Trial. Evid Based Complement Alternat Med 2015;2015:324360, doi:10.1155/2015/324360

60. Yeh ML, Chung YC, Chen KM, et al. Pain reduction of acupoint electrical stimulation for patients with spinal surgery: A placebo-controlled study. International Journal of Nursing Studies 2011;48(6):703-709, doi:https://doi.org/10.1016/j.ijnurstu.2010.10.009

61. Yeh ML, Chung YC, Chen KM, et al. Acupoint electrical stimulation reduces acute postoperative pain in surgical patients with patient-controlled analgesia: a randomized controlled study. Altern Ther Health Med 2010;16(6):10-8

62. Yeoh AH, Tang SS, Abdul Manap N, et al. Effectiveness of P6 acupoint electrical stimulation in preventing postoperativenausea and vomiting following laparoscopic surgery. Turkish Journal of Medical Sciences 2016;46(3):620-5, doi:10.3906/sag-1502-56

63. Yin XQ, Zhou YC, Zhou H, et al. Effect of transcutaneous electrical stimulation of Zusanli (ST 36) and Liangqiu (ST 34) combined with general anesthesia on pain and gastrointestinal symptoms in patients undergoing gynecological laparoscopic operation. Zhen CI yan jiu = acupuncture research 2013;38(6):431‐434

64. Yu JM, Qu PS, Fan H, et al. Observation on the analgesic effect of transcutaneous electrical acupoint stimulation for breast radical carcinoma operation. Zhen CI yan jiu = acupuncture research 2010;35(1):43‐46

65. Yu X, Zhang F, Chen B. The effect of TEAS on the quality of early recovery in patients undergoing gynecological laparoscopic surgery: a prospective, randomized, placebo-controlled trial. Trials 2020;21(1):43, doi:10.1186/s13063-019-3892-4

66. Zárate E, Mingus M, White PF, et al. The Use of Transcutaneous Acupoint Electrical Stimulation for Preventing Nausea and Vomiting After Laparoscopic Surgery. Anesthesia & Analgesia 2001;92(3):629-35.

67. Zhan W, Tian W. Addition of transcutaneous electric acupoint stimulation to transverse abdominis plane block for postoperative analgesia in abdominal surgery: a randomized controlled trial. European journal of integrative medicine 2020;35, doi:10.1016/j.eujim.2020.101087

68. Zhang B, Xu F, Hu P, et al. Needleless Transcutaneous Electrical Acustimulation: a Pilot Study Evaluating Improvement in Post-Operative Recovery. American journal of gastroenterology 2018;113(7):1026‐1035, doi:10.1038/s41395-018-0156-y

69. Zhang B, Zhu K, Hu P, et al. Needleless Transcutaneous Neuromodulation Accelerates Postoperative Recovery Mediated via Autonomic and Immuno-Cytokine Mechanisms in Patients With Cholecystolithiasis. Neuromodulation 2019;22(5):546‐554, doi:10.1111/ner.12856

70. Zhang H, Lei C, Zhang T, et al. Randomized controlled trial of TEAS with different acupoints combination on opioids consumption in patients undergoing off-pump coronary artery bypass grafting. International journal of clinical and experimental medicine 2016;9(12):23060‐23071

71. Zhang Q, Gao Z, Wang H, et al. The effect of pre-treatment with transcutaneous electrical acupoint stimulation on the quality of recovery after ambulatory breast surgery: a prospective, randomised controlled trial. Anaesthesia 2014;69(8):832‐839, doi:10.1111/anae.12639

72. Zhao F, Wang Z, Ye C, et al. Effect of Transcutaneous Electrical Acupoint Stimulation on One-Lung Ventilation-Induced Lung Injury in Patients Undergoing Esophageal Cancer Operation. Evidence-based complementary and alternative medicine 2020;2020, doi:10.1155/2020/9018701

73. Zhao FC, Gao YL, Cai HS, et al. Effect of transcutaneous electric acupoints stimulation on vascular endothelial function and inflammatory factors after percutaneous coronary intervention. Zhen CI yan jiu = acupuncture research 2021;46(8):684‐689, doi:10.13702/j.1000-0607.200784

74. Zhao W, Zhao X, Li J, et al. Clinical observation on controlling antihypertension with the general anesthesia of TEAS and anesthetics in endoscopic endonasal surgery. Zhongguo zhen jiu [Chinese acupuncture & moxibustion] 2015;35(12):1281-1284

75. Zhou D, Hu B, He S, et al. Transcutaneous Electrical Acupoint Stimulation Accelerates the Recovery of Gastrointestinal Function after Cesarean Section: A Randomized Controlled Trial. Evidence-Based Complementary and Alternative Medicine 2018;2018:7341920, doi:10.1155/2018/7341920

76. Zhou X, Cao SG, Tan XJ, et al. Effects of transcutaneous electrical acupoint stimulation (Teas) on postoperative recovery in patients with gastric cancer: A randomized controlled trial. Cancer Management and Research 2021;13:1449‐1458, doi:10.2147/CMAR.S292325

**eTable 1: Risk of Bias Assessment**

**Ao et al. “Effects of transcutaneous electrical acupoint stimulation on perioperative immune function and postoperative analgesia in patients undergoing radical mastectomy: A randomized controlled trial”.**

**RoB 2 tool**

| Bias | Author’s judgement | Support for judgement |
| --- | --- | --- |
| Bias arising from the randomization process | Low risk | Patients were randomly allocated to each group using sequentially‑numbered sealed envelopes and a random number generator. The baseline characteristics did not differ significantly between the groups. |
| Bias due to deviations from intended interventions | Low risk | None of the anesthesiologists, surgeons, acupuncturist, or patients were aware of the allocation. |
| Bias due to missing outcome data | Low risk | A total of one patient in the TEAS group and two patients in the sham TEAS group were excluded as they refused to receive TEAS after surgery. Similarly, two patients in the TEAS group had not completed all time point stimulations. |
| Bias in measurement of the outcome | Some concerns | No information. |
| Bias in selection of the reported result | High risk | The study was [completely](C:/Users/APPLE/AppData/Local/youdao/dict/Application/8.9.9.0/resultui/html/index.html#/javascript:;) not conducted in accordance with a pre-specified protocol (found on ClinicalTrials.gov). |

**Arnberger et al. “Monitoring of Neuromuscular Blockade at the P6 Acupuncture Point Reduces the Incidence of Postoperative Nausea and Vomiting”.**

**RoB 2 tool**

| Bias | Author’s judgement | Support for judgement |
| --- | --- | --- |
| Bias arising from the randomization process | Low risk | Patients were assigned to one of two groups using a set of computer-generated random numbers and the envelope numbers with the assignment were recorded. The baseline characteristics did not differ significantly between the groups. |
| Bias due to deviations from intended interventions | Low risk | Patients and PONV evaluators were not informed of the group assignments. The attending anesthesiologist could not be blinded to the group assignment, but he or she was not involved with the PONV assessment. |
| Bias due to missing outcome data | Low risk | Data were reasonably complete. |
| Bias in measurement of the outcome | Low risk | Patients were evaluated for nausea and vomiting by a blinded investigator who was not aware of the patients’ group assignments. |
| Bias in selection of the reported result | Some concerns | Insufficient information |

**Bai et al. “Effects of Transcutaneous Electrical Acupoint Stimulation on the Stress Response During Extubation After General Anesthesia in Elderly Patients Undergoing Elective Supratentorial Craniotomy: A Prospective Randomized Controlled Trial”.**

**RoB 2 tool**

| Bias | Author’s judgement | Support for judgement |
| --- | --- | --- |
| Bias arising from the randomization process | Low risk | The patients were randomly assigned either to the TEAS group or the control group in a 1:1 ratio using a computer-generated randomization number sequence. The group assignment was sealed in sequentially numbered opaque envelopes. The baseline characteristics did not differ significantly between the groups. |
| Bias due to deviations from intended interventions | Low risk | The patients, attending anesthesiologists, surgeons, and data collectors were all blinded to the group assignment. If it was found that patients knew or suspected their group allocation in the study, their data were excluded. |
| Bias due to missing outcome data | Low risk | Outcome data were available for all participants. |
| Bias in measurement of the outcome | Low risk | Data collectors (the anesthesiologists who were only responsible for collecting the data and did not attend the anesthesia management during the study) were blinded to the group assignment. |
| Bias in selection of the reported result | Low risk | The study was conducted in accordance with a pre-specified protocol. |

**Chen et al. “Efficacy of transcutaneous electrical acupoint stimulation combined with general anesthesia for sedation and postoperative analgesia in minimally invasive lung cancer surgery: A randomized, double-blind, placebo-controlled trial”.**

**RoB 2 tool judgement**

| Bias | Author’s | Support for judgement |
| --- | --- | --- |
| Bias arising from the randomization process | Some concerns | This study did not report details of allocation concealment. Computer-based sample randomization was simultaneously performed at the enrolment of each patient. The allocation code was generated by an independent statistician. The baseline characteristics did not differ significantly between the groups. |
| Bias due to deviations from intended interventions | Low risk | None of the anesthesiologists, surgeons, physicians in the post-  anesthesia care unit, or patients were aware of the allocation. |
| Bias due to missing outcome data | Low risk | Complete datasets were collected for all patients and the data were analyzed. |
| Bias in measurement of the outcome | Low risk | A randomized, double-blind, placebo-controlled study. |
| Bias in selection of the reported result | Low risk | The study was conducted in accordance with a pre-specified protocol. |

**Chen et al. “Transcutaneous electroacupuncture alleviates postoperative ileus after gastrectomy: A randomized clinical trial”.**

**RoB 2 tool**

| Bias | Author’s judgement | Support for judgement |
| --- | --- | --- |
| Bias arising from the randomization process | Some concerns | Eligible patients were randomly allocated by computer algorithm to the TEA or control group. The baseline characteristics did not differ significantly between the groups. However, there was insufficient information about the methods used to conceal the allocation. |
| Bias due to deviations from intended interventions | Some concerns | It was not double-blind research and could not exclude the placebo effect. |
| Bias due to missing outcome data | Low risk | Outcome data were available for all participants. |
| Bias in measurement of the outcome | Some concerns | No information. |
| Bias in selection of the reported result | Some concerns | Unclear if authors selected the reported outcome measures a priori |

**Chen, et al. “The Effect of Location of Transcutaneous Electrical Nerve Stimulation on Postoperative Opioid Analgesic Requirement: Acupoint Versus Nonacupoint Stimulation”.**

**RoB 2 tool**

| Bias | Author’s judgement | Support for judgement |
| --- | --- | --- |
| Bias arising from the randomization process | Some concerns | No specific randomization methods were mentioned. The baseline characteristics did not differ significantly between the groups. Nonetheless, the study did not report details of allocation concealment. |
| Bias due to deviations from intended interventions | Some concerns | Randomized, single-blind, sham-controlled study. |
| Bias due to missing outcome data | Low risk | Complete datasets were collected for all patients and the data were analyzed. |
| Bias in measurement of the outcome | Low risk | The observer was blind that did not aware of the TENS treatment group. |
| Bias in selection of the reported result | Some concerns | There is no information on whether the result being assessed but reported outcome data are unlikely to have been selected, on the basis of the results, from multiple eligible analyses of the da]ta. |

**Chen et al. “Effect of transcutaneous acupoint electric stimulation on perioperative intravenous anesthesia in patients of transsphenoidal pituitary tumor resection”.**

**RoB 2 tool**

| Bias | Author’s judgement | Support for judgement |
| --- | --- | --- |
| Bias arising from the randomization process | Some concerns | Patients were assigned to either the TEAS group or the control group by a table of random numbers. The baseline characteristics did not differ significantly between the groups. However, there was insufficient information about the methods used to conceal the allocation. |
| Bias due to deviations from intended interventions | Low risk | The anesthesia doctors and patients were not clear about the details and grouping of the acupoints of the body. |
| Bias due to missing outcome data | Low risk | Outcome data were available for all participants. |
| Bias in measurement of the outcome | Low risk | Assessment could not have been influenced by knowledge of intervention since the describe endpoint was relatively objective. |
| Bias in selection of the reported result | Low risk | The study is conducted in accordance with a pre-specified protocol (found on ClinicalTrials.gov). |

**Chen et al. “Does transcutaneous electric acupoint stimulation improve the quality of recovery after thyroidectomy? A prospective randomized controlled trial”.**

**RoB 2 tool**

| Bias | Author’s judgement | Support for judgement |
| --- | --- | --- |
| Bias arising from the randomization process | Low risk | Patients were assigned to either the TEAS group or the control group by a table of computer-generated random numbers. The allocation ratio was 1:1 for the two groups. Group assignments were sealed in sequentially numbered opaque envelopes. The baseline characteristics did not differ significantly between the groups. |
| Bias due to deviations from intended interventions | Low risk | The patients, attending anesthesiologist and surgeons were blinded to group assignment. |
| Bias due to missing outcome data | Low risk | Outcome data were available for all participants. |
| Bias in measurement of the outcome | Low risk | The data collector was blinded to group assignment. |
| Bias in selection of the reported result | Low risk | The study was conducted in accordance with a pre-specified protocol (found on ClinicalTrials.gov). |

**Chen et al. “Transcutaneous electric acupoint stimulation alleviates remifentanil-induced hyperalgesia in patients undergoing thyroidectomy: a randomized controlled trial”.**

**RoB 2 tool**

| Bias | Author’s judgement | Support for judgement |
| --- | --- | --- |
| Bias arising from the randomization process | Low risk | The randomization was performed in a 1:1 ratio according to a computer-generated list. Group assignments were concealed in sealed envelopes and assigned to either the TEAS group or the sham group. |
| Bias due to deviations from intended interventions | Low risk | All study personnel including the patients, investigator, attending anesthetist, surgeons, recovery ward nurses, and the person who performed the statistical analysis were blinded to group assignments. |
| Bias due to missing outcome data | Low risk | Outcome data were available for all participants. |
| Bias in measurement of the outcome | Low risk | The observer was blinded to group assignments. |
| Bias in selection of the reported result | Some concerns | Unclear if authors selected the reported outcome measures a priori |

**Chi et al. “Transcutaneous Electrical Acupoint Stimulation for Improving Postoperative Recovery, Reducing Stress and Inflammatory Responses in Elderly Patient Undergoing Knee Surgery”.**

**RoB 2 tool judgement**

| Bias | Author’s | Support for judgement |
| --- | --- | --- |
| Bias arising from the randomization process | Low risk | 52 subjects are randomly assigned to either Group E or Group C at a ratio of 1:1 on the basis of digital random numbers generated by SPSS.17.0 software. On the day of surgery, all subjects received the assigned intervention based on the group assignment in a concealed envelope after the subject was transported to the operating room with standard physiological monitors in place. |
| Bias due to deviations from intended interventions | Low risk | The acupuncturist/TEAS operator, statistician, data collector, trail administrator works independently and only perform the assigned task. There are not any private communications between all investigators regarding the study once protocol is initiate. And the patients had also no idea whether the electrodes were an authentic or a sham electrical stimulus. |
| Bias due to missing outcome data | Low risk | Complete datasets were collected for all patients and the data were analyzed. |
| Bias in measurement of the outcome | Some concerns | No information. |
| Bias in selection of the reported result | Some concerns | No information. |

**Chiu et al. “Effect of transcutaneous electrical nerve stimulation for pain relief on patients undergoing hemorrhoidectomy: prospective, randomized, controlled trial”.**

**RoB 2 tool judgement**

| Bias | Author’s | Support for judgement |
| --- | --- | --- |
| Bias arising from the randomization process | Some concerns | The patients were randomly assigned by selection from a random number table into the acupoint group and the nonpoint control group. This study did not report details of allocation concealment. |
| Bias due to deviations from intended interventions | Some concerns | Unclear if patients were blinded to group assignment. |
| Bias due to missing outcome data | Low risk | Complete datasets were collected for all patients and the data were analyzed. |
| Bias in measurement of the outcome | Some concerns | No information. |
| Bias in selection of the reported result | Some concerns | No information. |

**Ertas et al. “Effects of P6 Acustimulation with the ReliefBand on Postoperative Nausea and Vomiting in Patients Undergoing Gynecological Laparoscopy”**

**RoB 2 tool**

| Bias | Author’s judgement | Support for judgement |
| --- | --- | --- |
| Bias arising from the randomization process | Low risk | Randomization was performed using random numbers displayed on a list of codes prepared by a computerized system. These codes were written on paper slips, which were placed in numbered opaque sealed envelopes. The baseline characteristics did not differ significantly between the groups. |
| Bias due to deviations from intended interventions | Low risk | Research workers blinded to the study opened the envelopes and selected the appropriate ReliefBand. Thus, the patient and the research worker who held the records of the patient had no idea whether the ReliefBand was an authentic or a sham device. |
| Bias due to missing outcome data | Low risk | Outcome data were available for all participants. |
| Bias in measurement of the outcome | Some concerns | Assessment of clinical improvement may be minimally influenced by knowledge of intervention received. |
| Bias in selection of the reported result | Some concerns | No information. |

**Gan et al. “A Randomized Controlled Comparison of Electro-Acupoint Stimulation or Ondansetron Versus Placebo for the Prevention of Postoperative Nausea and Vomiting”.**

**RoB 2 tool**

| Bias | Author’s judgement | Support for judgement |
| --- | --- | --- |
| Bias arising from the randomization process | Low risk | Randomization was achieved using a random number generator in a sealed envelope technique. The baseline characteristics did not differ significantly between the groups. |
| Bias due to deviations from intended interventions | Low risk | According to the study masking was quadrupled (participants, care provider, investigator, outcome assessor) |
| Bias due to missing outcome data | Low risk | Outcome data were available for all eligible participants. |
| Bias in measurement of the outcome | Low risk | Postoperative data were collected by a separate research nurse not involved in the preoperative or intraoperative management of patients. |
| Bias in selection of the reported result | Some concerns | No information. |

**Gao et al. “Transcutaneous electrical acupoint stimulation for prevention of postoperative delirium in geriatric patients with silent lacunar infarction: a preliminary study”.**

**RoB 2 tool**

| Bias | Author’s judgement | Support for judgement |
| --- | --- | --- |
| Bias arising from the randomization process | Some concerns | The patients were divided into two groups by using a random number table. There were no significant differences in clinical characteristics between the two groups. But the allocation concealment was unclear. |
| Bias due to deviations from intended interventions | Some concerns | Unclear if patients were blinded to group assignment. |
| Bias due to missing outcome data | Low risk | Outcome data were available for all eligible participants. |
| Bias in measurement of the outcome | Low risk | The trained research personnel carrying out the assays was blinded to the group assignment. |
| Bias in selection of the reported result | Some concerns | No information as to whether authors selected outcomes measure a priori. |

**Gao et al. “Effect of transcutaneous electrical acupoint stimulation on catheter related bladder discomfort after ureteroscopic lithotripsy”.**

**RoB 2 tool**

| Bias | Author’s judgement | Support for judgement |
| --- | --- | --- |
| Bias arising from the randomization process | Some concerns | Patients were randomly enrolled to TEAS group and fake stimulation group by using Excel 2010 software. There were no significant differences in clinical characteristics between the two groups. But the allocation concealment was unclear. |
| Bias due to deviations from intended interventions | Some concerns | Unclear if patients were blinded to group assignment. |
| Bias due to missing outcome data | Low risk | 1 case in TEAS group and 2 cases in false stimulation group were excluded because of the change of surgical methods. There was no bias due to missing data. |
| Bias in measurement of the outcome | Low risk | Assessment of objective results may be minimally influenced by knowledge of intervention received. |
| Bias in selection of the reported result | Low risk | The study is conducted in accordance with a pre-specified protocol. |

**Gao et al. “Transcutaneous electrical acupoint stimulation applied in lower limbs decreases the incidence of paralytic ileus after colorectal surgery: A multicenter randomized controlled trial”.**

**RoB 2 tool**

| Bias | Author’s judgement | Support for judgement |
| --- | --- | --- |
| Bias arising from the randomization process | Low risk | SPSS software was used for block randomization in a 1:1 ratio with block size of 4. This random method will ensure that the number of patients in the 2 groups will be essentially equal with highly efficient enrollment. After rescreening for eligibility at the completion of surgery, the central randomization administrator opened the sealed envelope and made the group allocation to the intervention executor in the corresponding center. |
| Bias due to deviations from intended interventions | Low risk | All participants and study personnel, including researchers who collected data and anesthesiologists and surgeons who cared for patients, were blinded to the patient’s group allocation. |
| Bias due to missing outcome data | Low risk | Outcome data were available for all participants. |
| Bias in measurement of the outcome | Low risk | According to the protocol, outcome assessors was not able to be aware of intervention received. |
| Bias in selection of the reported result | Low risk | The study is conducted in accordance with a pre-specified protocol (found on ClinicalTrials.gov). |

**Gao et al. “Transcutaneous Electrical Acupoint Stimulation Decreases the Incidence of Postoperative Nausea and Vomiting After Laparoscopic Non-gastrointestinal Surgery: A Multi-Center Randomized Controlled Trial”.**

**RoB 2 tool**

| Bias | Author’s judgement | Support for judgement |
| --- | --- | --- |
| Bias arising from the randomization process | Some concerns | The eligible patients were randomly divided into the Sham and TEAS groups in a 1:1 ratio (with a block size of 4) by Crabyter scientific  research system (Xinyu Information Technology Co., Ltd.). There were no significant differences in clinical characteristics between the two groups. But the allocation concealment was unclear. |
| Bias due to deviations from intended interventions | Low risk | All patients, anesthesiologists, surgeons, and evaluators were blinded for group allocation, screening, intervention treatment, and statistical analysis. |
| Bias due to missing outcome data | Low risk | Outcome data were available for all participants. |
| Bias in measurement of the outcome | Low risk | Designated evaluators who were totally blinded took charge for follow-up data collections. |
| Bias in selection of the reported result | Low risk | The study is conducted in accordance with a pre-specified protocol (found on ClinicalTrials.gov). |

**Gao et al. “Clinical Trials for Thyroidectomy Under Acupuncture-aided Anesthesia by Using Electroacupuncture or Transcutaneous Acupoint Electrical Stimulation of Different Acupoints”.**

**RoB 2 tool**

| Bias | Author’s judgement | Support for judgement |
| --- | --- | --- |
| Bias arising from the randomization process | Some concerns | SAS 8.2 statistical software was used to generate the randomness according to the central area group randomization method scheme. The randomized protocol was developed and administered by specialists not involved in the trial. The acupuncture operator received the prescription for needle-assisted anesthesia 30min before surgery by random letter case. The baseline characteristics did not differ significantly between the groups. |
| Bias due to deviations from intended interventions | Some concerns | The experimenter might not be blinded to group allocation. |
| Bias due to missing outcome data | Low risk | Two cases were detached from each TEAS group and Local anesthesia group. There was no bias due to missing data. |
| Bias in measurement of the outcome | Low risk | Assessment of objective results may be minimally influenced by knowledge of intervention received. |
| Bias in selection of the reported result | Some concerns | No information as to whether authors selected outcomes measure a priori. |

**Ge et al. “Clinical efficacy of adductor canal blockade combined with transcutaneous electrical acupoint stimulation for total knee arthroplasty”.**

**RoB 2 tool**

| Bias | Author’s judgement | Support for judgement |
| --- | --- | --- |
| Bias arising from the randomization process | Some concerns | Each enrolled patient was randomized according to a computer-generated random numbers table. The baseline characteristics did not differ significantly between the groups. But there were no details of allocation concealment. |
| Bias due to deviations from intended interventions | Some concerns | The experimenters might not be blinded to group allocation. |
| Bias due to missing outcome data | Low risk | No missing data. |
| Bias in measurement of the outcome | Some concerns | No information. |
| Bias in selection of the reported result | Some concerns | No information as to whether authors selected outcomes measure a priori. |

**Gu a et al. “Effect of transcutaneous electrical acupoint stimulation on gastrointestinal function recovery after laparoscopic radical gastrectomy - A randomized controlled trial”.**

**RoB 2 tool**

| Bias | Author’s judgement | Support for judgement |
| --- | --- | --- |
| Bias arising from the randomization process | Some concerns | Each enrolled patient was randomized according to a computer-generated random numbers table. The baseline characteristics did not differ significantly between the groups. But there were no details of allocation concealment. |
| Bias due to deviations from intended interventions | Low risk | No patient had knowledge of TEAS prior to this study and were aware of the group allocations. |
| Bias due to missing outcome data | Low risk | No missing data. |
| Bias in measurement of the outcome | Low risk | Another investigator collected the data and was blind to the group allocation. Those who managed the data and statistical analysis were also blinded to the grouping of patients |
| Bias in selection of the reported result | Some concerns | Not enough information to determine selective reporting bias. |

**Guo et al. “****Transcutaneous electrical acupoint stimulation on inflammatory response and intestinal permeability in perioperative period of laparoscopic intestinal surgery”.**

**RoB 2 tool**

| Bias | Author’s judgement | Support for judgement |
| --- | --- | --- |
| Bias arising from the randomization process | Some concerns | Patients were assigned to either the TEAS group or the control group by a table of random numbers. The baseline characteristics did not differ significantly between the groups. Nonetheless, the study did not report details of allocation concealment. |
| Bias due to deviations from intended interventions | Low risk | Both patients and experimenters were unaware of the group allocations. |
| Bias due to missing outcome data | Low risk | Outcome data were available for all participants. |
| Bias in measurement of the outcome | Low risk | Those who managed the data and statistical analysis were also blinded to the grouping of patients. |
| Bias in selection of the reported result | Some concerns | Unclear if authors selected outcome measures a priori. |

**Habib et al. “****Transcutaneous acupoint electrical stimulation with the ReliefBand® for the prevention of nausea and vomiting during and after cesarean delivery under spinal anesthesia”.**

**RoB 2 tool**

| Bias | Author’s judgement | Support for judgement |
| --- | --- | --- |
| Bias arising from the randomization process | High risk | Although randomly grouped, it is not clear if hiding is assigned. Also, baseline was uneven between the two groups. |
| Bias due to deviations from intended interventions | Low risk | For blinding, the ReliefBand® was covered with opaque gauze that was taped to the wrist. |
| Bias due to missing outcome data | Low risk | Outcome data were available for all participants. |
| Bias in measurement of the outcome | Low risk | A separate researcher who was unaware of the patient’s randomization collected the data. |
| Bias in selection of the reported result | Some concerns | Unclear if authors selected outcome measures a priori. |

**He et al “analgesic effect of transcutaneous electrical acupoint stimulation combined with target-controlled infusion in general anesthesia and effects on cardiovascular system”**

**RoB 2 tool**

| Bias | Author’s judgement | Support for judgement |
| --- | --- | --- |
| Bias arising from the randomization process | Some concerns | Patients were assigned to either the TEAS group or the control group by a table of random numbers. The baseline characteristics did not differ significantly between the groups. But the details of concealed allocation were not reported. |
| Bias due to deviations from intended interventions | Some concerns | This is a single-blind trial. |
| Bias due to missing outcome data | Low risk | Outcome data were available for all participants. |
| Bias in measurement of the outcome | Some concerns | No information. |
| Bias in selection of the reported result | Low risk | While unclear if authors selected the reported outcome measures a prior, the outcome measures are objective; and all outcome measures agreed with each other. |

**Huang et al. “Prevention of propofol injection-related pain using pretreatment transcutaneous electrical acupoint stimulation.”**

| Bias | Author’s judgement | Support for judgement |
| --- | --- | --- |
| Bias arising from the randomization process | Some concerns | No specific randomization methods were mentioned. The baseline characteristics did not differ significantly between the groups. |
| Bias due to deviations from intended interventions | Some concerns | This was a randomized, double-blind study. Seven patients who were excluded had refused to participate in subsequent trials after pretreatment. (unclear) |
| Bias due to missing outcome data | Low risk | Outcome data were available for most of participants. |
| Bias in measurement of the outcome | Some concerns | Not mentioned |
| Bias in selection of the reported result | Some concerns | Not enough information. |

**Huang et al. “Effects of transcutaneous electrical acupoint stimulation at different frequencies on perioperative anesthetic dosage, recovery, complications, and prognosis in video‑assisted thoracic surgical lobectomy: a randomized, double‑blinded, placebo‑controlled trial”.**

**RoB 2 tool**

| Bias | Author’s judgement | Support for judgement |
| --- | --- | --- |
| Bias arising from the randomization process | Some concerns | Eligible participants were randomly assigned depending on enrollment sequence according to a series of random numbers generated by Excel. Randomization resulted in equal distribution of socio demographic and clinical characteristics, and surgery time was not statistically difference among groups. But the details of concealed allocation were not reported. |
| Bias due to deviations from intended interventions | Low risk | A randomized, double‑blinded, placebo‑controlled trial |
| Bias due to missing outcome data | Low risk | Outcome data were available for all participants. |
| Bias in measurement of the outcome | Low risk | The data were collected by another investigator unaware of the group allocation. This study had a strict double-blinded design. |
| Bias in selection of the reported result | Some concerns | The study was not done as the same as the pre-specified protocol designed, but the observed metrics were the same. |

**Huang et al. “****Effect of electrically stimulating acupoint, Zusanli (ST 36), on patient's recovery after laparoscopic colorectal cancer resection: a randomized controlled trial”.**

**RoB 2 tool**

| Bias | Author’s judgement | Support for judgement |
| --- | --- | --- |
| Bias arising from the randomization process | Low risk | The random method of the envelope method was used to randomly divide the subjects into two groups. Specific method: pre-establish random code, and seal each code into the envelope. The baseline characteristics did not differ significantly between the groups. |
| Bias due to deviations from intended interventions | Some concerns | A prospective randomized, single-blind trial. (unclear) |
| Bias due to missing outcome data | Low risk | Outcome data were available for all participants. |
| Bias in measurement of the outcome | Low risk | The data record of the experimental observation indicators is the responsibility of another investigator who is not familiar with all the trial protocols. |
| Bias in selection of the reported result | Low risk | The study provides outcome measures based on the study protocol published beforehand. |

**Huang et al. “Application of Transcutaneous Electrical Acupoint Stimulation Combined with Transversus Abdominis Plane Block to Enhanced Recovery After Surgery in Patients Undergoing Laparoscopic Colorectal Cancer Resection: a Randomized Controlled Clinical Trial”.**

**RoB 2 tool**

| Bias | Author’s judgement | Support for judgement |
| --- | --- | --- |
| Bias arising from the randomization process | Some concerns | Patients were randomized to TEAS group and TEAS+TAP group by a table of random numbers. The baseline characteristics did not differ significantly between the groups. But there were no details of allocation concealment. |
| Bias due to deviations from intended interventions | Some concerns | The experimenters might not be blinded to group allocation. |
| Bias due to missing outcome data | Low risk | No missing data. |
| Bias in measurement of the outcome | Some concerns | No information. |
| Bias in selection of the reported result | Some concerns | No information as to whether authors selected outcomes measure a priori. |

**Jin et al. “Clinical Study on the Combination of Transcutaneous Electrical Acupoint Stimulation and Lidocaine for Preventing Propofol Injection Pain”.**

**RoB 2 tool**

| Bias | Author’s judgement | Support for judgement |
| --- | --- | --- |
| Bias arising from the randomization process | Some concerns | Randomization was performed using the random number method. There were no statistically significant differences in the general information, hemodynamics, and basic conditions of anesthesia operation among each group. This study did not report details of allocation concealment. |
| Bias due to deviations from intended interventions | Some concerns | This a double-blind randomized control-group clinical trial. The details are unclear. |
| Bias due to missing outcome data | Low risk | Outcome data were available for all participants. |
| Bias in measurement of the outcome | Some concerns | Not mentioned |
| Bias in selection of the reported result | Low risk | In the study, outcome measures provide are in general accord in the study protocol published beforehand. |

**Lan et al. “****Transcutaneous electrical nerve stimulation on acupoints reduces fentanyl requirement for postoperative pain relief after total hip arthroplasty in elderly patients”.**

**RoB 2 tool**

| Bias | Author’s judgement | Support for judgement |
| --- | --- | --- |
| Bias arising from the randomization process | Some concerns | All patients were divided into two groups by use of a computer-generated random number table. The baseline characteristics did not differ significantly between the groups. This study did not report details of allocation concealment. |
| Bias due to deviations from intended interventions | Low risk | The patients, the orthopedic surgeon, nurses and physicians were blinded to group assignment. Only acupunctists who performed TENS were aware of patients’ allocation, however, interaction between the patient and acupuncturist was limited to the time required for attaching electrode pads on acupoints. |
| Bias due to missing outcome data | Low risk | Six patients lost to follow-up due to POCD; two patients discontinued intervention due to intraoperative severe bleeding. These woould not affect the final result. |
| Bias in measurement of the outcome | Low risk | All data were collected by the investigator who has no previous knowledge concerning TENS and was blinded to the patients’ group allocation. |
| Bias in selection of the reported result | Some concerns | No information. |

**Li et al. “Effect of Transcutaneous Electrical Acupoint Stimulation on Post-surgical Gastrointestinal Function, Autonomic Nerve Activities and Plasma Brain-gut Peptide Levels in Patient-s Undergoing Gastrointestinal Surgery”.**

**RoB 2 tool**

| Bias | Author’s judgement | Support for judgement |
| --- | --- | --- |
| Bias arising from the randomization process | Low risk | The patients were numbered and allocated into groups using a simple randomization of SPSS16.0 random numbers. The randomization schedule was kept in opaque sealed envelope. The baseline characteristics did not differ significantly between the groups. |
| Bias due to deviations from intended interventions | Low risk | The patients, anesthesiologist and surgeons were blinded to group assignment. |
| Bias due to missing outcome data | Low risk | Outcome data were available for all participants. |
| Bias in measurement of the outcome | Low risk | Outcome assessors were blinded to the intervention received by participants. |
| Bias in selection of the reported result | Some concerns | No information. |

**Li et al. “Transcutaneous electrical acupoint stimulation relieves post-operative nausea and vomiting possibly by reducing serum motilin secretion in patients undergoing laparoscopic surgery”.**

**RoB 2 tool**

| Bias | Author’s judgement | Support for judgement |
| --- | --- | --- |
| Bias arising from the randomization process | Low risk | The patients were numbered and allocated into groups using a simple randomization of SPSS22.0 random numbers. TEAS group or the sham TEAS group using sequentially‑numbered sealed envelopes and a random number generator and opened by the study coordinator in consecutive order. The baseline characteristics did not differ significantly between the groups. |
| Bias due to deviations from intended interventions | Some concerns | The experimenter might not be blinded to group allocation. |
| Bias due to missing outcome data | Low risk | Outcome data were available for all participants. |
| Bias in measurement of the outcome | Low risk | Assessment could not have been influenced by knowledge of intervention since the describe endpoints were relatively objective such as the serum MTL concentrations and PONV grade etc. |
| Bias in selection of the reported result | Some concern | No information provided |

**Li et al. “****Effects and Mechanisms of Transcutaneous Electrical Acustimulation on Postoperative Recovery After Elective Cesarean Section”.**

**RoB 2 tool**

| Bias | Author’s judgement | Support for judgement |
| --- | --- | --- |
| Bias arising from the randomization process | Some concerns | Participants were recruited immediately after CS and randomized (according to a random number table generated by SPSS 23.0) to receive TEA or sham-TEA treatment in a 1:1 ratio. The baseline characteristics did not differ significantly between the groups. This study did not report details of allocation concealment. |
| Bias due to deviations from intended interventions | Low risk | This a randomized, controlled, double-blind study. Both TEAS group and sham group received the same stimulation except the different points. |
| Bias due to missing outcome data | Low risk | Outcome data were available for all participants. |
| Bias in measurement of the outcome | Low risk | To ensure the double-blindness of the research, the treatment was done by the major investigator, while the data were collected and analyzed by a research team member who was blinded to the group information. |
| Bias in selection of the reported result | Some concern | No information provided |

**Li et al. “Perioperative transcutaneous electrical acupoint stimulation for improving postoperative gastrointestinal function: A randomized controlled trial”.**

**RoB 2 tool**

| Bias | Author’s judgement | Support for judgement |
| --- | --- | --- |
| Bias arising from the randomization process | Low risk | This procedure was conducted using the blocked randomization approach with a block length of four. Computer-generated random numbers were used to determine the allocation of blocks. The randomization schedule was kept in opaque sealed envelopes. The baseline characteristics of the 280 patients and there was no difference between the subgroups. |
| Bias due to deviations from intended interventions | Some concerns | A single-blinded randomized controlled trial. Observers responsible for postoperative follow-up and participants were blinded to the grouping information. |
| Bias due to missing outcome data | Low risk | No missing outcome data. |
| Bias in measurement of the outcome | Low risk | Outcome assessors were blinded to the intervention received by participants. The technicians did not participate in outcome measurements. |
| Bias in selection of the reported result | Low risk | The study was conducted in accordance with a pre-specified protocol. |

**Liang et al. “The Effect of Transcutaneous Electrical Acupoint Stimulation on Postoperative Catheter-Related Bladder Discomfort in Patients Undergoing Transurethral Resection of the Prostate”.**

**RoB 2 tool**

| Bias | Author’s judgement | Support for judgement |
| --- | --- | --- |
| Bias arising from the randomization process | Low risk | Eligible patients were randomly distributed into two groups, with the help of a computer-generated table of random numbers by an independent statistician. The independent statistician created identical sealed envelopes before surgery. The baseline characteristics did not differ significantly between the groups. |
| Bias due to deviations from intended interventions | Low risk | The prospective, randomized, controlled, double-blind study. |
| Bias due to missing outcome data | Low risk | Outcome data were available for all participants. |
| Bias in measurement of the outcome | Low risk | An anesthesiologist (HLD) performed general anesthesia and all intraoperative data recording, and another investigator (WL), in charge of all postoperative assessments, was also blinded to the group identity. Two investigators (YKN and JSH) performed data recording and analysis. |
| Bias in selection of the reported result | Low risk | The study is conducted in accordance with a pre-specified protocol. |

**Liu et al. “Effects of Transcutaneous Electrical Acupoint Stimulation on Postoperative Cognitive Decline in Elderly Patients: a Pilot Study”.**

**RoB 2 tool**

| Bias | Author’s judgement | Support for judgement |
| --- | --- | --- |
| Bias arising from the randomization process | Some concerns | According to a random number table, patients were randomly divided into two groups: control group (Group C) and TEAS group (Group T). The baseline characteristics did not differ significantly between the groups. This study did not report details of allocation concealment. |
| Bias due to deviations from intended interventions | Some concerns | The experimenter might not be blinded to group allocation. |
| Bias due to missing outcome data | Low risk | Data were reasonably complete. |
| Bias in measurement of the outcome | Low risk | The trained researchers who performed the MMSE score were blinded  to the grouping of patients. |
| Bias in selection of the reported result | Low risk | The outcomes were prespecified (ChiCTR2000040397). |

**Liu et al. “Intraoperative and postoperative anesthetic and analgesic effect of multipoint transcutaneous electrical acupuncture stimulation combined with sufentanil anesthesia in patients undergoing supratentorial craniotomy”.**

**RoB 2 tool**

| Bias | Author’s judgement | Support for judgement |
| --- | --- | --- |
| Bias arising from the randomization process | Low risk | Consecutive patients were enrolled and randomly allocated into the TEAS group or sham group according to a random number table generated by a computer. Only the acupuncturist was informed of the randomization allocation using opaque, sealed envelopes, just before the onset of TEAS. The baseline characteristics did not differ significantly between the groups. |
| Bias due to deviations from intended interventions | Low risk | The anesthesiologists, surgeons, recovery staff, assessors and participants, were blinded to the group allocation. |
| Bias due to missing outcome data | Low risk | Data were reasonably complete. |
| Bias in measurement of the outcome | Low risk | Postoperative data were collected by researchers who were also blinded to the study design. |
| Bias in selection of the reported result | Low risk | The primary outcome was prespecified (ChiCTR-TRC-10001078). |

**Liu et al. “Evaluation of Transcutaneous Electroacupoint Stimulation with the Train-of-four Mode for Preventing Nausea and Vomiting after Laparoscopic Cholecystectomy”.**

**RoB 2 tool**

| Bias | Author’s judgement | Support for judgement |
| --- | --- | --- |
| Bias arising from the randomization process | Some concerns | Patients were randomized into two groups of 48 into two groups using a table of random numbers. The baseline characteristics did not differ significantly between the groups. This study did not report details of allocation concealment. |
| Bias due to deviations from intended interventions | Low risk | This a randomized, controlled, double-blind study. |
| Bias due to missing outcome data | Low risk | Outcome data were available for all participants. |
| Bias in measurement of the outcome | Low risk | Postoperative data were collected by a separate research nurse who was not aware of the preoperative or perioperative management of patients. The anesthesiologists and care providers were blinded to the study group. |
| Bias in selection of the reported result | Some concerns | No information. |

**Lu et al. “Transcutaneous electrical acupoint stimulation before surgery reduces chronic pain after mastectomy: A randomized clinical trial”.**

**RoB 2 tool**

| Bias | Author’s judgement | Support for judgement |
| --- | --- | --- |
| Bias arising from the randomization process | Some concerns | Study participants were randomized 1:1:1 into three groups using a secured web-based system that was stratified according to permuted blocks (random block sizes; maximum of 12) by the treatment center. Patient baseline characteristics, anesthetic and surgical parameters, and postoperative treatments did not differ among the groups. However, the study was unclear on allocation concealment. |
| Bias due to deviations from intended interventions | Low risk | According to the protocol, masking was quadrupled (participants, care provider, investigator, outcome assessor) |
| Bias due to missing outcome data | Low risk | Outcome data were available for all participants. |
| Bias in measurement of the outcome | Low risk | Outcomes were assessed by trained investigators who were blinded to the treatment allocation |
| Bias in selection of the reported result | Low risk | The study is conducted in accordance with a pre-specified protocol (found on ClinicalTrials.gov). |

**Mi et al. “Effects of transcutaneous electrical acupoint stimulation on quality of recovery during early period after laparoscopic cholecystectomy”.**

**RoB 2 tool**

| Bias | Author’s judgement | Support for judgement |
| --- | --- | --- |
| Bias arising from the randomization process | Some concerns | The patients were randomly assigned according to the table of random digits. The baseline characteristics did not differ significantly between the groups. But the allocation concealment was unclear. |
| Bias due to deviations from intended interventions | Some concerns | No information. |
| Bias due to missing outcome data | Low risk | Data were complete. |
| Bias in measurement of the outcome | Some concerns | Clinical improvement on QoR-40 is potentially affected by physicians’ knowledge of intervention. |
| Bias in selection of the reported result | Some concerns | Not enough information. |

**Mu et al. “Effect of transcutaneous electrical acupoint stimulation on recovery of gastrointestinal function after cesarean section”.**

**RoB 2 tool**

| Bias | Author’s judgement | Support for judgement |
| --- | --- | --- |
| Bias arising from the randomization process | Some concerns | The patients were randomly assigned to TEAS group or control group according to simple random sample. The baseline characteristics did not differ significantly between the groups. But the allocation concealment was unclear. |
| Bias due to deviations from intended interventions | Some concerns | No information. |
| Bias due to missing outcome data | Low risk | Data were complete. |
| Bias in measurement of the outcome | Some concerns | The outcomes were not potentially affected by physicians’ knowledge of intervention. |
| Bias in selection of the reported result | Some concerns | Not enough information. |

**Oztas et al. “****The effects of two different electrical stimulation methods on the pain intensity of the patients who had undergone abdominal surgery with a midline incision: Randomized controlled clinical trial”.**

**RoB 2 tool**

| Bias | Author’s judgement | Support for judgement |
| --- | --- | --- |
| Bias arising from the randomization process | Some concerns | To determine the groups, randomization was performed with a web-based randomization system with the help of a computer by researchers. A block randomization list was obtained for 3 groups. The baseline characteristics did not differ significantly between the groups. However, this study did not report details of allocation concealment. |
| Bias due to deviations from intended interventions | High risk | The patients and data collector were aware they were implementation electrical stimulation or not, there was no blinding to the study. |
| Bias due to missing outcome data | Low risk | Outcome data were available for all participants. |
| Bias in measurement of the outcome | High risk | Data collector was aware they were implementation electrical stimulation or not, there was no blinding to the study. |
| Bias in selection of the reported result | Some concerns | No information. |

**Que et al. “Effects of Transcutaneous Electrical Acupoint Stimulation on Systemic Inflammatory Response Syndrome of Patients after Percutaneous Nephrolithotomy: a Randomized Controlled Trial”.**

**RoB 2 tool**

| Bias | Author’s judgement | Support for judgement |
| --- | --- | --- |
| Bias arising from the randomization process | Low risk | The perioperative management sequences of TEAS (group TEAS) and sham TEAS (group sham TEAS) were determined, and randomization was stratified according to the time of admission in a block size of 10. Eligible participants were randomly assigned to receive either TEAS or sham TEAS via a central randomization system for clinical research using a 1 :1 ratio. The random number list was generated by an independent statistician, and block size was disclosed to other researchers. An independent, blinded statistician concealed the file of the generated random number table using a password and provided information regarding which group the participant assigned. The baseline characteristics did not differ significantly between the groups. |
| Bias due to deviations from intended interventions | Low risk | For participants in group sham TEAS, the electrodes were also pasted on the same target acupoints, but the TEAS was not operated. All patients were informed that TEAS worked throughout the surgery. In addition, the patients involved in the study were treated separately to avoid mutual communication. |
| Bias due to missing outcome data | Low risk | Throughout the study, there were 2 surgeries cancelled in group TEAS, as well as 1 in group sham TEAS. One patient in group TEAS has potential difficult airway. One patient refused to receive TEAS for intolerance of electrical stimulation in group TEAS and two patients had severe complications during surgery. None of these would affect the final results. |
| Bias in measurement of the outcome | Low risk | Researchers who measured outcomes and researchers who performed data management and statistical analysis were blinded to each participant’s allocation status. The reviewer responsible for the statistical results was not allowed to engage in any dialogue with the participants. Practitioners were not involved in measuring treatment outcomes or data analysis. |
| Bias in selection of the reported result | Low risk | The study was conducted in accordance with a pre-specified protocol. (ChiCTR1800018254)) |

**Si et al. “Adjuvant effect of transcutaneous electrical acupoint stimulation in propofol-fentanyl anesthesia in partial mastectomy”.**

**RoB 2 tool**

| Bias | Author’s judgement | Support for judgement |
| --- | --- | --- |
| Bias arising from the randomization process | Some concerns | The patients were randomly assigned according to the table of random digits. The baseline characteristics did not differ significantly between the groups. But the allocation concealment was unclear. |
| Bias due to deviations from intended interventions | Some concerns | No information. |
| Bias due to missing outcome data | Low risk | Data were complete. |
| Bias in measurement of the outcome | Some concerns | The outcomes were not potentially affected by physicians’ knowledge of intervention. |
| Bias in selection of the reported result | Some concerns | Not enough information. |

**Song et al. “Effects of Transcutaneous Electrical Acupoint Stimulation on the Postoperative Sleep Quality and Pain of Patients After Video-Assisted Thoracoscopic Surgery: A Prospective, Randomized Controlled Trial”.**

**RoB 2 tool**

| Bias | Author’s judgement | Support for judgement |
| --- | --- | --- |
| Bias arising from the randomization process | Low risk | Eighty-five patients were divided to the TEAS group or control group randomly in a 1:1 ratio using a computer-generated randomization number sequence. Seal the group assignments in sequentially numbered opaque envelopes. The baseline characteristics did not differ significantly between the groups. |
| Bias due to deviations from intended interventions | Low risk | Patients, attending anesthesiologists and surgeons were all blinded to the group assignment. |
| Bias due to missing outcome data | Low risk | Outcome data were available for all participants. |
| Bias in measurement of the outcome | Low risk | Data collectors were blinded to the group assignment. |
| Bias in selection of the reported result | Low risk | The study was conducted in accordance with a pre-specified protocol (found on ClinicalTrials.gov). |

**Sun et al. “Perioperative Transcutaneous Electrical Acupoint Stimulation for Postoperative Pain Relief Following Laparoscopic Surgery A Randomized Controlled Trial”.**

**RoB 2 tool**

| Bias | Author’s judgement | Support for judgement |
| --- | --- | --- |
| Bias arising from the randomization process | Low risk | A SPSS-generated random number table was used to allocate the patients into 1 of the 4 groups in a 1:1:1:1 ratio. The TEAS-operators were informed the group allocation of the patient by a sealed opaque envelope and they were the only individuals aware of the treatment allocation. The baseline characteristics did not differ significantly between the groups. |
| Bias due to deviations from intended interventions | Low risk | Patients, anesthesiologists and evaluators were blinded to the treatment allocation. |
| Bias due to missing outcome data | Low risk | Outcome data were available for all participants. |
| Bias in measurement of the outcome | Low risk | Evaluators were blinded to the treatment allocation. |
| Bias in selection of the reported result | Low risk | The study was conducted in accordance with a pre-specified protocol (found on ClinicalTrials.gov). |

**Szmit et al. “Transcutaneous Electrical Acupoint Stimulation Reduces Postoperative Analgesic Requirement in Patients Undergoing Inguinal Hernia Repair: A Randomized, Placebo-Controlled Study”.**

**RoB 2 tool**

| Bias | Author’s judgement | Support for judgement |
| --- | --- | --- |
| Bias arising from the randomization process | Some concerns | Enrolled participants were randomly assigned to each group. An independent, blinded statistician generated the block randomization scheme. The baseline characteristics did not differ significantly between the groups. But the details of concealed allocation were not reported. |
| Bias due to deviations from intended interventions | Low risk | The participants were blinded to the type of treatment. Mock TEAS was provided with the same lamplight as real TEAS, so the participants were not able to predict the allocated group based on the appearance of the treatment. |
| Bias due to missing outcome data | Low risk | Outcome data were available for all participants. |
| Bias in measurement of the outcome | Low risk | The table was managed by an independent researcher who was not involved in the recruitment, treatment, or assessment. |
| Bias in selection of the reported result | Low risk | The study was conducted in accordance with a pre-specified protocol. |

**Tu et al. “Transcutaneous Electrical Acupoint Stimulation Improves Immunological Function During the Perioperative Period in Patients with Non-Small Cell Lung Cancer Undergoing Video-Assisted Thoracic Surgical Lobectomy”.**

**RoB 2 tool**

| Bias | Author’s judgement | Support for judgement |
| --- | --- | --- |
| Bias arising from the randomization process | Some concerns | Eligible participants were randomly assigned to receive either TEAS or sham TEAS via a central randomization system for clinical research using a 1:1 ratio. The baseline characteristics did not differ significantly between the groups. But the study did not provide sufficient detail on the methods used to conceal allocation sequences. |
| Bias due to deviations from intended interventions | Low risk | The chief surgeon, patients and laboratory physicians were blinded to the study group assignments. |
| Bias due to missing outcome data | Low risk | Outcome data were available for all participants. |
| Bias in measurement of the outcome | Low risk | Researchers who assessed outcome measures and those who performed data management and statistical analysis were blinded to each participant’s allocation status. |
| Bias in selection of the reported result | Some concerns | Not enough information to determine selective reporting bias. |

**Wang et al. “Effect of the Intensity of Transcutaneous Acupoint Electrical Stimulation on the Postoperative Analgesic Requirement”.**

**RoB 2 tool**

| Bias | Author’s judgement | Support for judgement |
| --- | --- | --- |
| Bias arising from the randomization process | Some concerns | Patients were assigned to each group according to a computer-generated randomization sequence. The analgesic treatment groups were comparable with respect to both demographic and clinical variables. But we could not know if the trial have adequately concealed allocation. |
| Bias due to deviations from intended interventions | Low risk | The was a present prospective, randomized, sham-controlled, and single-blind study. Although a double-blind study design would have been preferable, the investigator bias was minimized by using only objective data in the statistical analyses. |
| Bias due to missing outcome data | Low risk | Outcome data were available for all participants. |
| Bias in measurement of the outcome | Some concerns | No information. |
| Bias in selection of the reported result | Some concerns | Unclear if authors selected the reported outcome measures a priori. |

**Wang et al. “Effects of transcutaneous electrical acupoint stimulation on treatment and prevention of postoperative sore throat after tracheal intubation under general anesthesia”.**

**RoB 2 tool**

| Bias | Author’s judgement | Support for judgement |
| --- | --- | --- |
| Bias arising from the randomization process | Some concerns | The patients were randomly assigned according to the table of random digits. The baseline characteristics did not differ significantly between the groups. But the allocation concealment was unclear. |
| Bias due to deviations from intended interventions | Some concerns | No information. |
| Bias due to missing outcome data | Low risk | Outcome data were available for all participants. |
| Bias in measurement of the outcome | Some concerns | No information. |
| Bias in selection of the reported result | Some concerns | Unclear if authors selected the reported outcome measures a priori. |

**Wang et al. “Transcutaneous electric acupoint stimulation reduces intra-operative remifentanil consumption and alleviates postoperative side-effects in patients undergoing sinusotomy: a prospective, randomized, placebo-controlled trial”.**

**RoB 2 tool**

| Bias | Author’s judgement | Support for judgement |
| --- | --- | --- |
| Bias arising from the randomization process | Some concerns | Patients were assigned to either TEAS group or control group on the basis of random numbers generated by a computer. The baseline characteristics did not differ significantly between the groups. The allocation concealment was unclear. |
| Bias due to deviations from intended interventions | Low risk | Blinding of the patients was ensured by using gel electrodes in the same therapeutic setting. None of the anesthesiologists, surgeons, physicians in the post-anesthesia care unit (PACU), or participants were aware of the allocation. |
| Bias due to missing outcome data | Low risk | Complete datasets were collected for all the 60 participants and all the data were analyzed. |
| Bias in measurement of the outcome | Low risk | Outcome assessors were blinded to the intervention received by participants. |
| Bias in selection of the reported result | Low risk | The study was conducted in accordance with a pre-specified protocol (found on ClinicalTrials.gov). |

**Wang et al. “Effect of transcutaneous acupoint electrical stimulation on blood bioactive compounds involving cerebral injury during craniotomy”.**

**RoB 2 tool**

| Bias | Author’s judgement | Support for judgement |
| --- | --- | --- |
| Bias arising from the randomization process | Some concerns | A randomized block design was performed. The baseline characteristics did not differ significantly between the groups. There was insufficient information about the methods used to conceal the allocation |
| Bias due to deviations from intended interventions | Some concerns | No information. |
| Bias due to missing outcome data | Low risk | Data were available from all patients who underwent treatment in each arm. |
| Bias in measurement of the outcome | Low risk | Assessment could not have been influenced by knowledge of intervention since the describe endpoint was relatively objective such as the contents of IL-6 etc. |
| Bias in selection of the reported result | Some concerns | Not enough information to determine selective reporting bias. |

**Wang et al. “Electroacupoint Stimulation for Postoperative Nausea and Vomiting in Patients Undergoing Supratentorial Craniotomy”.**

**RoB 2 tool**

| Bias | Author’s judgement | Support for judgement |
| --- | --- | --- |
| Bias arising from the randomization process | Some concerns | Patients were randomized into 2 groups using a computer-generated random number table. The baseline characteristics did not differ significantly between the groups. But there were no details of allocation concealment. |
| Bias due to deviations from intended interventions | Low risk | None of the patients had experience with acupuncture electrodes. The patients were unaware whether the sensation was coming from an acupoint or a non-acupoint. |
| Bias due to missing outcome data | Low risk | Outcome data were available for all participants. |
| Bias in measurement of the outcome | Low risk | The screen on the unit was covered with an opaque tape in both groups, so that clinicians and observers were unaware whether the unit was at an acupoint or not. Trained nurse staff did the PONV assessment. They were blind to the position of the electrode. |
| Bias in selection of the reported result | Some concerns | Not enough information to determine selective reporting bias. |

**Wu et al. “Effects of transcutaneous acupoint electrical stimulation on the imbalance of Th1, Th2, Th17 and Treg cells following thoracotomy of patients with lung cancer”.**

**RoB 2 tool**

| Bias | Author’s judgement | Support for judgement |
| --- | --- | --- |
| Bias arising from the randomization process | Low risk | The subjects were randomized equally into three groups, using a computer-generated random list with coded sealed envelopes. The baseline characteristics did not differ significantly between the groups. |
| Bias due to deviations from intended interventions | Some concerns | The acupoints of all of the patients were covered with cutaneous self-adhesive electrode pads and informed that they may or may not feel the current. However, it is unclear whether the implementers knew each patient’s allocation. |
| Bias due to missing outcome data | Low risk | Outcome data were available for all participants. |
| Bias in measurement of the outcome | Some concerns | No information. |
| Bias in selection of the reported result | Some concerns | Unclear if authors selected the reported outcome measures a priori |

**Wu et al. “Effect of Transcutaneous Acupoint Electrical Stimulation Combined with Target Controlled Infusion of Propofol on Efficacy of General Anesthesia for Craniotomy”.**

**RoB 2 tool**

| Bias | Author’s judgement | Support for judgement |
| --- | --- | --- |
| Bias arising from the randomization process | Some concerns | Randomization was stratified by site and nature, using a block randomization procedure. The baseline characteristics did not differ significantly between the groups. The allocation scheme concealment was unclear. |
| Bias due to deviations from intended interventions | Some concerns | No information. |
| Bias due to missing outcome data | Low risk | Data were available from all patients who underwent treatment in each arm. |
| Bias in measurement of the outcome | Low risk | The describe endpoint was relatively objective which could not be influenced by the assessor’s knowledge of intervention. |
| Bias in selection of the reported result | Some concerns | Not enough information to determine selective reporting bias. |

**Xing et al. “Study on the value of transcutaneous acupoint electrical stimulation with general intravenous anesthesia in subtotal thyroidectomy surgery”.**

**RoB 2 tool**

| Bias | Author’s judgement | Support for judgement |
| --- | --- | --- |
| Bias arising from the randomization process | Some concerns | No specific randomization methods were mentioned. The baseline characteristics did not differ significantly between the groups. |
| Bias due to deviations from intended interventions | Some concerns | Unclear if patients were blinded to group assignment. |
| Bias due to missing outcome data | Low risk | Outcome data were available for all participants. |
| Bias in measurement of the outcome | Low risk | Assessment could not have been influenced by knowledge of intervention since the describe endpoint was relatively objective. |
| Bias in selection of the reported result | Some concerns | No information. |

**Xiong et al. “Transcutaneous Electrical Acupoint Stimulation Combined with Dexamethasone and Tropisetron Prevents Postoperative Nausea and Vomiting in Female Patients Undergoing Laparoscopic Sleeve Gastrectomy: a Prospective, Randomized Controlled Trial”.**

**RoB 2 tool**

| Bias | Author’s judgement | Support for judgement |
| --- | --- | --- |
| Bias arising from the randomization process | Low risk | After enrolment into the study, the patients were randomly assigned to the TEAS or control groups using a computerized random number generator. Group assignment was exposed from a sealed envelope only by an acupuncturist. The baseline characteristics did not differ significantly between the groups. |
| Bias due to deviations from intended interventions | Low risk | The patients were blinded to the group assignment. The acupoint stimulation instrument was covered with an opaque box for adequate blinding. |
| Bias due to missing outcome data | Low risk | No missing outcome data. |
| Bias in measurement of the outcome | Low risk | An anesthetic resident who was not involved in the anesthesia routine and who was blinded to the group assignments performed the follow-up and data collection. |
| Bias in selection of the reported result | Low risk | The study is conducted in accordance with a pre-specified protocol (found on ClinicalTrials.gov). |

**Xu et al. “The Effects of P6 Electrical Acustimulation on Postoperative Nausea and Vomiting in Patients After Infratentorial Craniotomy”.**

**RoB 2 tool**

| Bias | Author’s judgement | Support for judgement |
| --- | --- | --- |
| Bias arising from the randomization process | Low risk | Using a computer-generated random number table and allocation concealment of sequentially numbered, opaque, sealed envelopes, eligible patients were randomized into groups. The baseline characteristics did not differ significantly between the groups. |
| Bias due to deviations from intended interventions | Low risk | The display screens of the units were concealed from view for patients and other investigators. The anesthesiologists attending to the patients were not aware if the stimulating unit was switched on or off. |
| Bias due to missing outcome data | Low risk | Data were complete. |
| Bias in measurement of the outcome | Low risk | Primary outcomes were opioid-related side effects, which could not be influenced by the assessor’s knowledge of intervention. |
| Bias in selection of the reported result | Some concerns | Not enough information to determine selective reporting bias. |

**Yang et al. “Dexamethasone alone vs in combination with transcutaneous electrical acupoint stimulation or tropisetron for prevention of postoperative nausea and vomiting in gynaecological patients undergoing laparoscopic surgery”.**

**RoB 2 tool**

| Bias | Author’s judgement | Support for judgement |
| --- | --- | --- |
| Bias arising from the randomization process | Some concerns | They were randomly allocated using a computer-generated table into three groups. The baseline characteristics did not differ significantly between the groups. But we could not know if the trial have adequately concealed allocation. |
| Bias due to deviations from intended interventions | Low risk | For adequate blinding, an opaque tape was applied to the patient’s wrist above the electrodes, and the patient’s arm was wrapped in a blanket. The patients, the anesthetists, and the nursing staff were unaware of the group assignments. |
| Bias due to missing outcome data | Low risk | Two patients did not have follow-up outcome data because of losing contact after discharge. But it has no effect on the results. |
| Bias in measurement of the outcome | Low risk | An anesthetic nurse who was unaware of the group assignments and trained for the study and blinded to the randomization did the follow-up and data collection. |
| Bias in selection of the reported result | Low risk | The study is conducted in accordance with a pre-specified protocol (found on ClinicalTrials.gov). |

**Yao et al. “Transcutaneous Electrical Acupoint Stimulation Improves the Postoperative Quality of Recovery and Analgesia after Gynecological Laparoscopic Surgery: A Randomized Controlled Trial.”**

**RoB 2 tool**

| Bias | Author’s judgement | Support for judgement |
| --- | --- | --- |
| Bias arising from the randomization process | Low risk | Patients were assigned to each group by a table of computer-generated random numbers. Group assignments were sealed in sequentially numbered opaque envelopes. The baseline characteristics did not differ significantly between the groups. |
| Bias due to deviations from intended interventions | Low risk | A prospective, randomized, double-blind, placebo-controlled study |
| Bias due to missing outcome data | Low risk | Outcome data were available for all participants. |
| Bias in measurement of the outcome | Low risk | Data collectors and the person who performed the final statistical analysis were blinding to group assignment. |
| Bias in selection of the reported result | Some concerns | No information. |

**Yeh et al. “Pain reduction of acupoint electrical stimulation for patients with spinal surgery: A placebo-controlled study”.**

**RoB 2 tool**

| Bias | Author’s judgement | Support for judgement |
| --- | --- | --- |
| Bias arising from the randomization process | High risk | The randomization was achieved through a computer-generated numbers and information on intervention allocation sequential without sealed envelopes. |
| Bias due to deviations from intended interventions | Low risk | Individual subjects and medical staff did not know in which group the subjects were. |
| Bias due to missing outcome data | Low risk | Outcome data were available for all participants. |
| Bias in measurement of the outcome | Some concerns | The possibility of the threat to internal validity due to a single-blind design may not be avoided. |
| Bias in selection of the reported result | Some concerns | No information. |

**Yeh et al. “Acupoint electrical stimulation reduces acute postoperative pain in surgical patients with patient-controlled analgesia: a randomized controlled study”.**

**RoB 2 tool**

| Bias | Author’s judgement | Support for judgement |
| --- | --- | --- |
| Bias arising from the randomization process | Some concerns | No specific randomization methods were mentioned. The baseline characteristics did not differ significantly between the groups. |
| Bias due to deviations from intended interventions | Low risk | Patients and their nurses and physicians were not informed of the group allocation. |
| Bias due to missing outcome data | Low risk | Outcome data were available for all participants. |
| Bias in measurement of the outcome | Some concerns | No information. |
| Bias in selection of the reported result | Some concerns | Insufficient information |

**Yeoh et al. “Effectiveness of P6 acupoint electrical stimulation in preventing postoperative nausea and vomiting following laparoscopic surgery”.**

**RoB 2 tool**

| Bias | Author’s judgement | Support for judgement |
| --- | --- | --- |
| Bias arising from the randomization process | Some concerns | The patients were randomly allocated to each group by using a random sequence of computer-generated numbers. Patient demographic characteristics and factors likely to influence PONV were not significantly different between the active and sham groups. But the allocation concealment was unclear. |
| Bias due to deviations from intended interventions | Low risk | It was inactivated by placing a silicone cover over the electrodes, which was invisible to both patients and investigators. |
| Bias due to missing outcome data | Low risk | Outcome data were available for all participants. |
| Bias in measurement of the outcome | Low risk | The investigators responsible for data collection were blinded to the treatments administered to the study patients. |
| Bias in selection of the reported result | Some concerns | No information. |

**Yin et al. “Effect of Transcutaneous Electrical Stimulation of Zusanli (ST 36) and Liangqiu (ST 34) combined with General Anesthesia on Pain and Gastrointestinal Symptoms in Patients Undergoing Gynecological Laparoscopic Operation”.**

**RoB 2 tool**

| Bias | Author’s judgement | Support for judgement |
| --- | --- | --- |
| Bias arising from the randomization process | Some concerns | Patients were assigned to either the TEAS group or the control group by the table of random numbers. The baseline characteristics did not differ significantly between the groups. The allocation concealment was unclear. |
| Bias due to deviations from intended interventions | Some concerns | Participants might be aware of assignment and could have disclosed information or had biased expectations from assignment |
| Bias due to missing outcome data | Low risk | Data were presented as complete. |
| Bias in measurement of the outcome | Some concerns | VAS was the subjective assessment of pain severity, and outcome measure was possibly to have been impacted by the knowledge of intervention received. |
| Bias in selection of the reported result | Some concerns | Unclear if authors selected outcome measures a priori. |

**Yu et al. “Observation on the analgesic effect of transcutaneous electrical acupoint stimulation for breast radical carcinoma operation”.**

**RoB 2 tool**

| Bias | Author’s judgement | Support for judgement |
| --- | --- | --- |
| Bias arising from the randomization process | Some concerns | Patients were assigned to either the TEAS group or the control group by a table of random numbers. The baseline characteristics did not differ significantly between the groups. But the study did not report details of allocation concealment. |
| Bias due to deviations from intended interventions | Some concerns | No information. |
| Bias due to missing outcome data | Low risk | Outcome data were available for all participants. |
| Bias in measurement of the outcome | Some concerns | No information. |
| Bias in selection of the reported result | Some concerns | Not enough information to determine selective reporting bias. |

**Yu et al. “The effect of TEAS on the quality of early recovery in patients undergoing gynecological laparoscopic surgery: a prospective, randomized, placebo-controlled trial”.**

**RoB 2 tool**

| Bias | Author’s judgement | Support for judgement |
| --- | --- | --- |
| Bias arising from the randomization process | Some concerns | Patients were assigned to either TEAS group or control group on the basis of random numbers generated by a computer. The baseline characteristics did not differ significantly between the groups. The study did not report details of allocation concealment |
| Bias due to deviations from intended interventions | Low risk | None of the anesthesiologists, surgeons, physicians in the post-anesthesia care unit (PACU), or participants were aware of the allocation. |
| Bias due to missing outcome data | Low risk | Outcome data were available for all participants. |
| Bias in measurement of the outcome | Low risk | Assessors were unaware of intervention received by study participants. |
| Bias in selection of the reported result | Low risk | The study was conducted in accordance with a pre-specified protocol (found on ClinicalTrials.gov). |

**Zárate et al. “The Use of Transcutaneous Acupoint Electrical Stimulation for Preventing Nausea and Vomiting After Laparoscopic Surgery”.**

**RoB 2 tool**

| Bias | Author’s judgement | Support for judgement |
| --- | --- | --- |
| Bias arising from the randomization process | Some concerns | Outpatients who had been fasted overnight were randomly assigned to one of three treatment groups with a computer-generated random number table. The three treatment groups were comparable demographically. But the allocation concealment was unclear. |
| Bias due to deviations from intended interventions | Low risk | This study was a multicenter, randomized, double-blinded, placebo- and sham-controlled study. The recovery room nursing staff were unaware of the treatment group to which the patient had been assigned. |
| Bias due to missing outcome data | Low risk | Outcome data were available for all participants. |
| Bias in measurement of the outcome | Some concerns | No information. |
| Bias in selection of the reported result | Some concerns | No information. |

**Zhan et al. “Addition of transcutaneous electric acupoint stimulation to transverse abdominis plane block for postoperative analgesia in abdominal surgery: a randomized controlled trial”.**

**RoB 2 tool**

| Bias | Author’s judgement | Support for judgement |
| --- | --- | --- |
| Bias arising from the randomization process | Low risk | Each enrolled patient was randomized according to a computer-generated random numbers table, and then an independent research assistant informed different treating anesthetists based on results. The baseline characteristics did not differ significantly between the groups. |
| Bias due to deviations from intended interventions | Low risk | This study was a single blind randomized control trail. The assessor, treating anesthetists and statistical analysis were blinded from group allocation. |
| Bias due to missing outcome data | Low risk | Outcome data were available for all participants. |
| Bias in measurement of the outcome | Some concerns | The assessor is not aware of the analgesic treatment the patient is receiving. |
| Bias in selection of the reported result | Low risk | The study was conducted in accordance with a pre-specified protocol. |

**Zhang et al. “Needleless Transcutaneous Electrical Acustimulation: A Pilot Study Evaluating Improvement in Post-Operative Recovery”.**

**RoB 2 tool**

| Bias | Author’s judgement | Support for judgement |
| --- | --- | --- |
| Bias arising from the randomization process | Some concerns | Patients were randomized into two groups: TEA and sham-TEA. The baseline characteristics did not differ significantly between the groups. This study did not report details of allocation concealment. |
| Bias due to deviations from intended interventions | Some concerns | No information. |
| Bias due to missing outcome data | Low risk | Data were complete. |
| Bias in measurement of the outcome | Low risk | It was recorded by a caring physician who did not know the study design. All blood assays were performed blindly by a professional company (Shanghai Dian Medical Testing Laboratory). |
| Bias in selection of the reported result | Some concerns | No information provided. |

**Zhang et al. “Needleless Transcutaneous Neuromodulation Accelerates Postoperative Recovery Mediated via Autonomic and Immuno-Cytokine Mechanisms in Patients with Cholecystolithiasis”.**

**RoB 2 tool**

| Bias | Author’s judgement | Support for judgement |
| --- | --- | --- |
| Bias arising from the randomization process | Some concerns | Randomization: 1) we generated 56 random numbers by excel; 2) each enrolled subject got one random number; 3) random numbers were sorted by order. The baseline characteristics did not differ significantly between the groups. This study lacked of details of allocation concealment. |
| Bias due to deviations from intended interventions | Low risk | The investigators and patients were blinded to the nature of the therapy. |
| Bias due to missing outcome data | Low risk | Data were reasonably complete. |
| Bias in measurement of the outcome | Low risk | The investigators and patients were blinded to the nature of the therapy. |
| Bias in selection of the reported result | Some concerns | No information provided. |

**Zhang et al. “Randomized controlled trial of TEAS with different acupoints combination on opioids consumption in patients undergoing off-pump coronary artery bypass grafting”.**

**RoB 2 tool**

| Bias | Author’s judgement | Support for judgement |
| --- | --- | --- |
| Bias arising from the randomization process | Some concerns | Although randomly grouped, it is not clear if hiding is assigned. The baseline characteristics did not differ significantly between the groups. |
| Bias due to deviations from intended interventions | Low risk | The patients, the staff involved in intraoperative care (anesthesia and the cardiac surgical team) and postoperative care (the CCU physicians), the investigators who obtained and documented data and performed follow-up assessment, and the clinical endpoint committee were unaware of the study group assignments. |
| Bias due to missing outcome data | Low risk | Data were reasonably complete. |
| Bias in measurement of the outcome | Low risk | The site investigators were unaware of the study group assignments until the data were unblinding in March 2015. |
| Bias in selection of the reported result | Low risk | The study was conducted in accordance with a pre-specified protocol (found on ClinicalTrials.gov). |

**Zhang et al. “The effect of pre-treatment with transcutaneous electrical acupoint stimulation on the quality of recovery after ambulatory breast surgery: a prospective, randomized controlled trial”.**

**RoB 2 tool**

| Bias | Author’s judgement | Support for judgement |
| --- | --- | --- |
| Bias arising from the randomization process | Some concerns | Patients were assigned to either the TEAS group or the sham group using a randomization sequence based on a table of randomly generated numbers. The baseline characteristics did not differ significantly between the groups. But there were no details of allocation concealment. |
| Bias due to deviations from intended interventions | Low risk | A controlled, double-blinded clinical trial. This study’s strengths include adequate blinding, standardized TEAS and anesthesia protocols |
| Bias due to missing outcome data | Low risk | Outcome data were available for all participants. |
| Bias in measurement of the outcome | Low risk | Both the randomization and allocation lists were concealed from the anesthetists who gave the general anesthetic to study patients, and from the persons who performed the final statistical analysis. |
| Bias in selection of the reported result | Low risk | The timepoint of VAS was changed, but deemed at low risk of selective reporting bias. |

**Zhao et al. “Effect of Transcutaneous Electrical Acupoint Stimulation on One-Lung Ventilation-Induced Lung Injury in Patients Undergoing Esophageal Cancer Operation.”**

**RoB 2 tool**

| Bias | Author’s judgement | Support for judgement |
| --- | --- | --- |
| Bias arising from the randomization process | Some concerns | No specific randomization methods were mentioned. The baseline characteristics did not differ significantly between the groups. This study did not report details of allocation concealment. |
| Bias due to deviations from intended interventions | Some concerns | All patients were told that TEAS was carried out throughout the surgery and were treated separately to prevent communication. But experimenters might know the experimental interventions. |
| Bias due to missing outcome data | Low risk | Outcome data were available for all eligible participants. |
| Bias in measurement of the outcome | Low risk | Assessment could not have been influenced by knowledge of intervention since the describe endpoints were relatively objective such as the serum level of IL-6 and TNF-α. Besides, the laboratory physicians were blinded to the work of research team. |
| Bias in selection of the reported result | Some concerns | No information. |

**Zhao et al. “Effect of transcutaneous electric acupoints stimulation on vascular endothelial function and inflammatory factors after percutaneous coronary intervention”.**

**RoB 2 tool**

| Bias | Author’s judgement | Support for judgement |
| --- | --- | --- |
| Bias arising from the randomization process | Some concerns | The patients were randomly assigned according to the table of random digits. The baseline characteristics did not differ significantly between the groups. But the allocation concealment was unclear. |
| Bias due to deviations from intended interventions | Some concerns | No information. |
| Bias due to missing outcome data | Low risk | Outcome data were available for all participants. |
| Bias in measurement of the outcome | Some concerns | No information. |
| Bias in selection of the reported result | Some concerns | Unclear if authors selected the reported outcome measures a priori. |

**Zhao et al. “Clinical observation on controlling antihypertension with the general anesthesia of TEAS and anesthetics in endoscopic endonasal surgery”.**

**RoB 2 tool**

| Bias | Author’s judgement | Support for judgement |
| --- | --- | --- |
| Bias arising from the randomization process | Some concerns | Sixty patients were randomly divided into a TEAS group and a sham-TEAS group by using a random number table. The baseline characteristics did not differ significantly between the groups. But the allocation concealment was unclear. |
| Bias due to deviations from intended interventions | Some concerns | No information. |
| Bias due to missing outcome data | Low risk | Outcome data were available for all participants. |
| Bias in measurement of the outcome | Some concerns | No information. |
| Bias in selection of the reported result | Some concerns | No information as to whether authors selected outcomes measure a priori. |

**Zhou et al. “Transcutaneous Electrical Acupoint Stimulation Accelerates the Recovery of Gastrointestinal Function after Cesarean Section: A Randomized Controlled Trial”.**

**RoB 2 tool**

| Bias | Author’s judgement | Support for judgement |
| --- | --- | --- |
| Bias arising from the randomization process | Low risk | The participants were randomly allocated to the study groups using a computerized random number generation program by PASS 11.0. Allocation concealment was enclosed in sealed, opaque, sequentially numbered envelopes which will be opened only upon arrival of the participant in the operation room.Randomization was implemented by the research designer, who did not participate in recruitment and data collection. The baseline characteristics did not differ significantly between the groups. |
| Bias due to deviations from intended interventions | Low risk | All subjects and research staff involved in recruitment, data collection, and statistical analysis were blinded to the randomization status throughout the study. Only the acupuncturist knew about the interventions the subjects received, but he was not involved in the research process, except for the implementation of TEAS. He was not involved in data collection and was asked to maintain the secrecy about the interventions he provided. |
| Bias due to missing outcome data | Low risk | Outcome data were available for all participants. |
| Bias in measurement of the outcome | Low risk | Statistical analysis were blinded to the randomization status throughout the study. |
| Bias in selection of the reported result | Low risk | The study was conducted in accordance with a pre-specified protocol (found on ClinicalTrials.gov). |

**Zhou et al. “Effects of Transcutaneous Electrical Acupoint Stimulation (TEAS) on Postoperative Recovery in Patients with Gastric Cancer: A Randomized Controlled Trial”.**

**RoB 2 tool**

| Bias | Author’s judgement | Support for judgement |
| --- | --- | --- |
| Bias arising from the randomization process | Some concerns | To determine the groups, randomization was performed with a web-based randomization system with the help of a computer by researchers. A block randomization list was obtained for 3 groups. The baseline characteristics did not differ significantly between the groups. However, this study did not report details of allocation concealment. |
| Bias due to deviations from intended interventions | High risk | This was an unblinded randomized controlled trial. |
| Bias due to missing outcome data | Low risk | Outcome data were available for all participants. |
| Bias in measurement of the outcome | High risk | This was an unblinded randomized controlled trial. |
| Bias in selection of the reported result | Low risk | The study was conducted in accordance with a pre-specified protocol (found on ClinicalTrials.gov). |

**
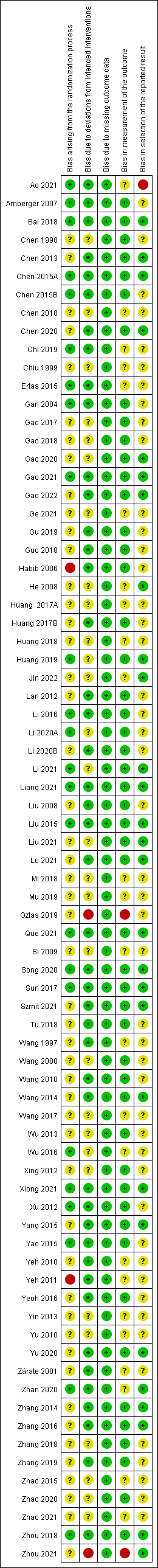
**

**eFigure 1:** Cross Tabulation Risk of Bias. Green circle= low bias risk, yellow circle= unclear bias risk, red circle= high bias risk.

**eTable 2: GRADE quality of evidence summary table.**

| **Primary Outcomes for Transcutaneous Electrical Acupoint Stimulation on Perioperative Analgesia** | | | | | |
| --- | --- | --- | --- | --- | --- |
| **Patient or population:** Patients Adults who were scheduled for elective surgery **Settings:** Randomized controlled trials (RCTs) **Intervention:** TEAS  **Comparison:** Sham-TEAS or Non-TEAS | | | | | |
| Outcomes | Illustrative comparative risks* (97.5%/99% CI) | | No of Participants (studies) | Quality of the evidence (GRADE) | Comments |
|  |  | Corresponding risk |  |  |  |
| **Cumulative intravenous morphine equivalent consumption within 24 hours after surgery (mg)**^†^ |  | The mean 24 hour in the intervention groups was **14.60 lower** (23.60 lower to 5.60 lower) | 455 (6 studies) | ⊕⊕○○ **low**^1,2^ | TEAS likely results in **a certain decrease** in the Cumulative intravenous morphine equivalent consumption within 24 hours after surgery (mg). |
| **Rest pain scores at 2 hours (cm)**^‡^ |  | The mean 2 hours in the intervention groups was **0.96 lower** (1.44 lower to 0.48 lower) | 401 (6 studies) | ⊕⊕○○ **low**^1,2^ | TEAS likely results in **a certain decrease** in the rest pain scores at 2 hours. |
| **Rest pain scores at 6 hours (cm)**^‡^ |  | The mean 6 hours in the intervention groups was **0.74 lower** (1.83 lower to 0.35 higher) | 619 (7 studies) | ⊕⊕○○ **low**^2,3^ | TEAS likely results in **little to no difference** in the rest pain scores at 6 hours. |
| **Rest pain scores at 12 hours (cm)**^‡^ |  | The mean 12 hours in the intervention groups was **1.02 lower** (1.87 lower to 0.17 lower) | 457 (6 studies) | ⊕⊕○○ **low**^1,2^ | TEAS likely results in **a certain decrease** in the rest pain scores at 12 hours. |
| **Rest pain scores at 24 hours (cm)**^‡^ |  | The mean 24 hours in the intervention groups was **0.79 lower** (1.25 lower to 0.32 lower) | 3743  (21 studies) | ⊕⊕○○ **low**^1,2^ | TEAS likely results in **a certain decrease** in the rest pain scores at 24 hours. |

| **Primary Outcomes for Transcutaneous Electrical Acupoint Stimulation on Perioperative Analgesia** | | | | | | |
| --- | --- | --- | --- | --- | --- | --- |
| **Patient or population:** Adults who were scheduled for elective surgery **Settings:** Randomized controlled trials (RCTs) **Intervention:** TEAS  **Comparison:** Sham-TEAS or Non-TEAS | | | | | | |
| **Secondary Outcomes** | **Illustrative comparative risks* (95%/99% CI)** | | **Relative effect (95%/99% CI)** | **No of Participants (studies)** | **Quality of the evidence (GRADE)** | **Comments** |
|  | **Assumed risk** | **Corresponding risk** |  |  |  |  |
|  | **Control** | **Secondary Outcomes** |  |  |  |  |
| **Cumulative intravenous morphine equivalent consumption (mg) within 48 hours after surgery (mg)** |  | The mean 48h in the intervention groups was **20.20 lower** (28.06 lower to 12.33 lower) |  | 400 (5 studies) | ⊕⊕⊕○ **moderate**^2^ | TEAS likely results in **a certain decrease** in Cumulative intravenous morphine equivalent consumption (mg) within 48 hours after surgery. |
| **Rest pain scores at 48 hours (cm)**^‡^ |  | The mean 48h in the intervention groups was **0.57 lower** (0.97 lower to 0.18 lower) |  | 925 (12 studies) | ⊕⊕○○ **low**^1,2^ | TEAS likely results in **a certain decrease** in the rest pain scores at 48 hours. |
| **Rest pain scores at 72 hours (cm)**^‡^ |  | The mean 72h in the intervention groups was **0.78 lower** (1.70 lower to 0.14 higher) |  | 442 (6 studies) | ⊕○○○ **very low** ^2,3,4^ | TEAS likely results in **little to no difference** in the rest pain scores at 72 hours. |
| **Rescue analgesia rate within 24 hours after surgery** | **41 per 595** | **70 per 396** | **RR 0.53**  (0.38 to 0.74) | 991 (6 studies) | ⊕⊕⊕⊕ **high** | TEAS likely results in **a certain decrease** in the rescue analgesia rate within 24 hours after surgery |
| **Rescue analgesia rate within 48 hours after surgery** | **36 per 351** | **35 per 170** | **RR 0.45**  (0.29 to 0.70) | 521 (3 studies) | ⊕⊕⊕⊕ **high** | TEAS likely results in **a certain decrease** in the rescue analgesia rate within 48 hours after surgery |
| **The intraoperative consumption of propofol (mg)** |  | The mean propofol (mg) in the intervention groups was **28.87 lower** (52.25 lower to 5.50 lower) |  | 4069 (15 studies) | ⊕⊕⊕○ **moderate**^2^ | TEAS likely results in **a certain decrease** in the intraoperative consumption of propofol. |
| **The intraoperative consumption of fentanyl (μg)** |  | The mean fentanyl (μg) in the intervention groups was **20.36 lower** (40.01 lower to 0.72 lower) |  | 1023 (11 studies) | ⊕⊕⊕○ **moderate**^2^ | TEAS likely results in **a certain decrease** in the intraoperative consumption of fentanyl. |
| **The intraoperative consumption of remifentanil (μg)** |  | The mean remifentanil (μg) in the intervention groups was **128.41lower** (183.28 lower to 73.55 lower) |  | 4085 (16 studies) | ⊕⊕○○ **low**^2,4^ | TEAS likely results in **a certain decrease** in the intraoperative consumption of remifentanil. |
| **The intraoperative consumption of sufentanil (μg)** |  | The mean sufentanil (μg) in the intervention groups was **0.03 higher** (1.01 lower to 1.07 higher) |  | 2823 (8 studies) | ⊕⊕○○ **low**^2,3^ | TEAS likely results in**a certain decrease** in the intraoperative consumption of sufentanil. |
| **TNF-α st 24 hours(pg/mL)**^‡^ |  | The mean 24h TNF-α in the intervention groups was **19.31 higher** (44.72 lower to 6.11 higher) |  | 382 (5 studies) | ⊕⊕○○ **low**^2,3^ | TEAS likely results in**a certain decrease** in 24 hours TNF-α |
| **IL-6** **at 24 hours (pg/mL)**^‡^ |  | The mean 24 IL-6 in the intervention groups was **12.05 lower** (15.86 lower to 8.23 lower) |  | 586 (8 studies) | ⊕⊕⊕○ **moderate**^2^ | TEAS likely results in **a certain decrease** in 24 hours IL-6 |
| **NE at 24 hours (pg/mL)**^‡^ |  | The mean 24h NE in the intervention groups was **68.37 lower** (200.49 lower to 63.74 higher) |  | 102 (2 studies) | ⊕○○○ **very low** ^2,3,4^ | TEAS likely results in **little to no difference** in 24 hours NE |
| **TNF-α at 48 hours (pg/mL)**^‡^ |  | The mean 48h TNF-α in the intervention groups was **15.91 lower** (27.89 lower to 3.93 lower) |  | 120 (2 studies) | ⊕⊕⊕○ **moderate**^2^ | TEAS likely results in **a certain decrease** in 48 hours TNF-α |
| **IL-6 at 48 hours (pg/mL)**^‡^ |  | The mean 48h IL-6 in the intervention groups was **19.48 lower** (45.34 lower to 6.38 lower) |  | 170 (3 studies) | ⊕⊕○○ **low**^2,3^ | TEAS likely results in **a certain decrease** in 48 hours IL-6 |
| **TNF-α at 72 hours (pg/mL)**^‡^ |  | The mean 72h TNF-α in the intervention groups was 0.35 **lower** (1.90 lower to 1.20 high) |  | 168 (2 studies) | ⊕⊕○○ **low**^2,3^ | TEAS likely results in **little to no difference** in 72 hours TNF-α |
| **IL-6 at 72 hours (pg/mL)**^‡^ |  | The mean 72h IL-6 in the intervention groups was **7.37 lower** (13.90 lower to 0.84 lower) |  | 838 (4 studies) | ⊕⊕⊕○ **moderate**^2^ | TEAS likely results in **la certain decrease** in 72 hours IL-6 |
| **QoR-40 at 24 hours** |  | The mean 24h QoR-40 in the intervention groups was **10.64** **higher** (6.14 higher to 15.14 higher) |  | 511 (7 studies) | ⊕⊕⊕○ **moderate**^2^ | TEAS likely results in **a certain [increase](javascript:;)** in 24 hours QoR-40 |
| **QoR-40 at 48 hours** |  | The mean 48h QoR-40 in the intervention groups was **1.91 higher** (0.97 higher to 2.84 higher) |  | 230 (3 studies) | ⊕⊕⊕⊕ **high** | TEAS likely results in **a certain increase** in 48 hours QoR-40 |
| **The time to first flatus (hours)** |  | The mean the time to first flatus in the intervention groups was **11.17 lower** (15.35 lower to 7.00 lower) |  | 2223 (16 studies) | ⊕⊕○○ **low**^1,2^ | TEAS likely results in **a certain decrease** in the time to first flatus |
| **The time to first defecation (hours)** |  | The mean the time to first defecation in the intervention groups was **15.88 lower** (21.15 lower to 10.62 lower) |  | 1974 (12 studies) | ⊕⊕○○ **low**^1,2^ | TEAS likely results in **a certain decrease** in the time to first defecation |
| **The time to first feeding (hours)** |  | The mean the time to first feed in the intervention groups was **10.64 lower** (18.01 lower to 3.27 lower) |  | 596 (9 studies) | ⊕⊕○○ **low**^2,4^ | TEAS likely results in **a certain decrease** in the time to first feeding |
| **The time to first ambulation (hours)** |  | The mean the time to first feed in the intervention groups was **11.29 lower** (27.86 lower to 5.28 high) |  | 662 (7 studies) | ⊕○○○ **very low**^2,3,4^ | TEAS likely results in **little to no difference** in the time to first ambulation |
| **The time to first bowel sound (hours)** |  | The mean the time to first blow sound in the intervention groups was **4.86 lower** (6.25 lower to 3.46 lower) |  | 895 (4 studies) | ⊕⊕⊕⊕ **high** | TEAS likely results in **a certain decrease** in the time to first bowel sound |
| **Length of hospital stay (days)** |  | The mean the time to first blow sound in the intervention groups was **0.98 lower** (1.37 lower to 0.59 lower) |  | 2151 (21 studies) | ⊕⊕○○ **low**^1,2^ | TEAS likely results in **a certain decrease** in the length of hospital stay |
| **PON in 24 hours after surgery** | **250 per 1005** | **403 per 874** | **RR 0.63**  (0.53 to 0.76) | 1879 (15 studies) | ⊕⊕○○ **low**^1,2^ | TEAS likely results in **a certain decrease** in PON within 24 hours after surgery |
| **PON in 48 hours after surgery** | **91 per 301** | **37 per 120** | **RR 0.71**  (0.21 to 2.40) | 421 (2 studies) | ⊕⊕○○ **low**^2,4^ | TEAS likely results in **a certain decrease** in PON within 48 hours after surgery |
| **POV in 24 hours after surgery** | **242 per 1768** | **369 per 1615** | **RR 0.63**  (0.54 to 0.73) | 3383 (14 studies) | ⊕⊕⊕○ **moderate**^1^ | TEAS likely results in **a certain decrease** in POV within 24 hours after surgery |
| **POV in 48 hours after surgery** | **87 per 332** | **51 per 151** | **RR 0.65**  (0.30 to 1.40) | 483 (3 studies) | ⊕⊕⊕○ **moderate**^2^ | TEAS likely results in **little to no difference** in POV within 48 hours after surgery |
| **PONV in 24 hours after surgery** | **523 per 2001** | **675 per 1766** | **RR 0.67** (0.61 to 0.73) | 3767 (19 studies) | ⊕⊕⊕○ **moderate**^2^ | TEAS likely results in **a certain decrease** within PONV in 24 hours after surgery |
| **PONV in 48 hours after surgery** | **34 per 153** | **58 per 114** | **RR 0.47**  (0.33 to 0.66) | 267 (4 studies) | ⊕⊕⊕⊕ **high** | TEAS likely results in **a certain decrease** in PONV within 48 hours after surgery |
| **Dizziness in 24 hours after surgery** | **382 per 1062** | **524 per 1116** | **RR 0.56**  (0.41 to 0.77) | 2178 (8 studies) | ⊕⊕○○ **low**^2,4^ | TEAS likely results in **a certain decrease** in dizziness within 24 hours after surgery |
| **Dizziness in 48 hours after surgery** | **19 per 92** | **30 per 93** | **RR 0.64**  (0.39 to 1.04) | 185 (3 studies) | ⊕⊕⊕○ **moderate**^4^ | TEAS likely results in **a certain decrease** in dizziness within 48 hours after surgery |
| **Pruritus in 24 hours after surgery** | **16 per 88** | **54 per 137** | **RR 0.49**  (0.21 to 1.16) | 225 (3 studies) | ⊕⊕○○ **low**^2,4^ | TEAS likely results in **a certain decrease** in pruritus within 24 hours after surgery |
| **Pruritus in 48 hours after surgery** | **10 per 92** | **15 per 93** | **RR 0.67** (0.32 to 1.41) | 185 (3 studies) | ⊕⊕⊕○ **moderate**^4^ | TEAS likely results in **little to no difference** in pruritus within 48 hours after surgery |
| **Antiemetic rate in 24 hours after surgery** | **84 per 371** | **152 per 417** | **RR 0.66** (0.53 to 0.82) | 788 (9 studies) | ⊕⊕⊕○ **moderate**^1^ | TEAS likely results in **a certain decrease** in antiemetic rate within 24 hours after surgery |
| **Antiemetic rate in 48 hours after surgery** | **82 per 302** | **39 per 121** | **RR 0.79**  (0.35 to 1.78) | 423 (2 studies) | ⊕⊕○○ **low**^2,4^ | TEAS likely results in **a certain decrease** in antiemetic rate within 24 hours after surgery |
| *The basis for the **assumed risk** (e.g. the median control group risk across studies) is provided in footnotes. The **corresponding risk** (and its 95% confidence interval) is based on the assumed risk in the comparison group and the **relative effect** of the intervention (and its 95% CI). **CI:** Confidence interval; **RR:** Risk ratio;  GRADE Working Group grades of evidence **High quality:** Further research is very unlikely to change our confidence in the estimate of effect.  **Moderate quality:** Further research is likely to have an important impact on our confidence in the estimate of effect and may change the estimate. **Low quality:** Further research is very likely to have an important impact on our confidence in the estimate of effect and is likely to change the estimate. **Very low quality:** We are very uncertain about the estimate.  ^1^ 'Some concerns' in Risk of Bias ^2^ High heterogeneity (I^2^ > 50%) which could not be resolved by sub-group analysis or meta-regression  ^3^ Some evidence of imprecision as CI cross invalid line  ^4^ Having evidence of publication bias  ^†^ 97.5% CI  ^‡^ 99% CI | | | | | | |

| \| **eTable 3: Predefined sensitivity analyses for primary outcomes** \| \| \| \| \| \| \| --- \| --- \| --- \| --- \| --- \| --- \| \| **Deleted Studies** \| **WMD (99% CI)** \| **Model** \| ***P*** \| ***I^2^* test (%)** \| **Number of studies** \| \| **The cumulative intravenous morphine equivalent consumption within 24 hours after surgery** \| \| \| \| \| \| \| Yeh 2011 \| -16.93 [-27.59, -6.27] \| Random \| 0.0004 \| 97.75 \| 5 \| \| **Rest pain scores at 2 hours after surgery** \| \| \| \| \| \| \| Oztas 2019 \| -0.87 [-1.34, -0.40] \| Random \| < 0.00001 \| 65 \| 5 \| \| **Rest pain scores at 12 hours after surgery** \| \| \| \| \| \| \| Ao 2021 \| -1.06 [-2.05, -0.07] \| Random \| 0.006 \| 98 \| 5 \| \| **Rest pain scores at 24 hours after surgery** \| \| \| \| \| \| \| Ao 2021 \| -0.80 [-1.29, -0.31] \| Random \| < 0.0001 \| 98 \| 20 \| \| **Abbreviation:** WMD: weighted mean difference; CI: confidence interval. \| \| \| \| \| \| |
| --- | --- | --- | --- | --- | --- | --- | --- | --- | --- | --- | --- | --- | --- | --- | --- | --- | --- | --- | --- | --- | --- | --- | --- | --- | --- | --- | --- | --- | --- | --- | --- | --- | --- | --- | --- | --- | --- | --- | --- | --- | --- | --- | --- | --- | --- | --- | --- | --- | --- | --- | --- | --- | --- | --- | --- | --- | --- | --- | --- | --- | --- | --- | --- | --- | --- | --- |

| **eTable 4: Subgroup analyses for primary outcomes** | | | | | | | |
| --- | --- | --- | --- | --- | --- | --- | --- |
| **Subgroup** | **Subgroup category** | **Included studies** | **N studies** | **WMD (97.5/99% CI)** | **Model** | ***P*** | ***I^2^* test (%)** |
| **The cumulative intravenous morphine equivalent consumption within 24 hours after surgery (97.5% CI)** | | | | | | | |
| The types of surgery | Open surgery | Chiu 1999, Lan2012,  Yeh 2010, Yeh 2011 | 4 | -11.65 [-21.11, -2.19] | Random | < 0.001 | 89 |
|  | Minimally invasive surgery | Chen 2020, Szmit 2021 | 2 | -17.98 [-40.77, 4.82] | Random | < 0.001 | 99 |
| The intervention of control group | Sham-TEAS | Chiu 1999, Yeh 2010, Yeh 2011 | 3 | -5.33 [-6.36, -4.30] | Random | 0.41 | 0 |
|  | Non-TEAS | Chen 2020, Lan2012, Szmit 2021, Yeh 2010, Yeh 2011 | 5 | -19.03 [-32.13, -5.93] | Random | < 0.001 | 96 |
| Trial registration in advance | Yes | Chen 2020, Szmit 2021 | 2 | -17.98 [-40.77, 4.82] | Random | < 0.001 | 99 |
|  | No | Chiu 1999, Lan2012,  Yeh 2010, Yeh 2011 | 4 | -11.65 [-21.11, -2.19] | Random | < 0.001 | 89 |
| Risk of bias | Low risk | Chen 2020, Lan2012, Szmit 2021 | 3 | -19.01 [-38.18, 0.17] | Random | < 0.001 | 98 |
|  | unclear/high risk | Chiu 1999, Yeh 2010, Yeh 2011 | 3 | -5.97 [-12.12, 0.18] | Random | 0.01 | 60 |
| **Rest pain scores at 2 hours after surgery (99% CI)** | | | | | | | |
| The types of surgery | Open surgery | Oztas 2019, Wu 2016, Zhan 2020 | 3 | -1.09 [-2.20, 0.02] | Random | 0.01 | 65 |
|  | Minimally invasive surgery | Yao 2015, Song 2020, Gao 2020 | 3 | -0.81 [-1.33, -0.30] | Random | < 0.001 | 49 |
| The intervention of control group | Sham-TEAS | Oztas 2019, Song 2020, Wu 2016 | 3 | -0.88 [-1.12, -0.63] | Random | < 0.001 | 0 |
|  | Non-TEAS | Gao 2020, Oztas 2019, Wu 2016, Yao 2015, Zhan 2020 | 5 | -1.08 [-1.90, -0.27] | Random | < 0.001 | 84 |
| Trial registration in advance | Yes | Gao 2020, Song 2020, Zhan 2020 | 3 | -0.62 [-1.29, 0.05] | Random | 0.02 | 47 |
|  | No | Oztas 2019, Wu 2016, Yao 2015 | 3 | -1.17 [-1.50, -0.83] | Random | < 0.001 | 12 |
| Risk of bias | Low risk | Yao 2015, Song 2020, Gao 2020, Zhan 2020 | 4 | -0.75 [-1.28, 0.21] | Random | < 0.001 | 48 |
|  | unclear/high risk | Oztas 2019, Wu 2016 | 2 | -1.36 [-2.24,-0.47] | Random | < 0.001 | 52 |
| **Rest pain scores at 6 hours after surgery (99% CI)** | | | | | | | |
| The types of surgery | Open surgery | Ge 2021, Wang 2017,  Wu 2016, Zhou 2018 | 4 | -0.91 [-1.77, -0.06] | Random | 0.006 | 94 |
|  | Minimally invasive surgery | Chen 2020, Gao 2020, Song 2020 | 3 | -0.95 [-2.01, 0.11] | Random | 0.29 | 99 |
| The intervention of control group | Sham-TEAS | Song 2020, Wu 2016, Zhou 2018, | 3 | -0.58 [-1.60, 0.44] | Random | 0.14 | 92 |
|  | Non-TEAS | Chen 2020, Wu 2016, Gao 2020, Ge 2021, Wang 2017, Zhou 2018 | 6 | -0.79 [-2.07, 0.50] | Random | 0.11 | 98 |
| Trial registration in advance | Yes | Chen 2020, Gao 2020,  Song 2020, Zhou 2018 | 4 | -0.73 [-2.56, 1.10] | Random | 0.31 | 99 |
|  | No | Ge 2021, Wang 2017, Wu 2016 | 3 | -0.76 [-1.89, 0.37] | Random | 0.08 | 95 |
| Risk of bias | Low risk | Chen 2020, Gao 2020,  Song 2020, Zhou 2018 | 4 | -0.73 [-2.56, 1.10] | Random | 0.31 | 99 |
|  | unclear/high risk | Ge 2021, Wang 2017, Wu 2016 | 3 | -0.76 [-1.89, 0.37] | Random | 0.08 | 95 |
| **Rest pain scores at 12 hours after surgery (99% CI)** | | | | | | | |
| The intervention of control group | Sham-TEAS | Chiu 1999, Wu 2016 | 2 | -1.68 [-1.80, -1.55] | Random | < 0.001 | 96 |
|  | Non-TEAS | Ao 2021, Ge 2021, Huang 2018, Wang 2017, Wu 2016 | 5 | -1.15 [-1.27, -1.03] | Random | < 0.001 | 97 |
| **Rest pain scores at 24 hours after surgery (99% CI)** | | | | | | | |
| The types of anaesthesia | General anesthesia | Ao 2021, Chen 2020, Gao 2022, Gu 2019, Jin 2022, Liu 2015, Lu 2021, Song 2020, Wang 2017, Wu 2016, Yao 2015, Yin 2013, Yu 2020, Zhang 2018 | 14 | -0.90 [-1.69, -0.11] | Random | 0.003 | 98 |
|  | Local anesthesia | Chiu 1999, Lan 2012, Li 2020B, Zhou 2018 | 4 | -0.63 [-1.27, 0.02] | Random | 0.01 | 96 |
|  | General anesthesia + Local anesthesia | Ge 2021, Huang 2018, Zhan 2020 | 3 | -0.46 [-1.25, 0.33] | Random | 0.13 | 78 |
| The types of surgery | Open surgery | Ao 2021, Chiu 19, 99, Ge 2021, Lan 2012, Li 2020B, Liu 2015, Lu 2021, Wang 2017, Wu 2016, Zhan 2020, Zhang 2018, Zhou 2018 | 12 | -0.69 [-1.09, -0.28] | Random | < 0.001 | 95 |
|  | Minimally invasive surgery | Chen 2020, Gao 2022, Gu 2019, Huang 2018, Jin 2022, Song 2020, Yao 2015, Yin 2013, Yu 2020 | 9 | -0.87 [-2.02, 0.29] | Random | 0.05 | 99 |
| The implementation of TEAS timing | Preoperative TEAS | Jin 2022, Lu 2021, Wang 2017,  Yao 2015, Yu 2020 | 5 | -0.70 [-1.74, 0.34] | Random | 0.08 | 94 |
|  | Postoperative TEAS | Chiu 1999, Ge 2021,  Li 2020B, Zhang 2018 | 4 | -0.64 [-1.30, 0.01] | Random | 0.01 | 97 |
|  | Preoperative+ intraoperative TEAS | Huang 2018, Liu 2015, Yin 2013 | 3 | -0.96 [-2.48, 0.55] | Random | 0.10 | 95 |
|  | Preoperative+ postoperative TEAS | Ao 2021, Lan 2012, Song 2020, Wu 2016, Zhan 2020, Zhou 2018 | 6 | -0.58 [-1.34, 0.19] | Random | 0.05 | 92 |
|  | Full perioperative TEAS | Chen 2020, Gao 2022, Gu 2019 | 3 | -1.33 [-3.67, 1.00] | Random | 0.14 | 100 |
| The intervention of control group | Sham-TEAS | Chiu 1999, Li 2020B, Song 2020, Wu 2016, Zhang 2018, Zhou 2018 | 6 | -0.64 [-1.19, -0.08] | Random | 0.003 | 96 |
|  | Non-TEAS | Ao 2021, Chen 2020, Gao 2022, Ge 2021, Gu 2019, Huang 2018, Jin 2022, Lan 2012, Liu 2015, Lu 2021, Wang 2017, Wu 2016, Yao 2015, Yin 2013, Yu 2020, Zhan 2020, Zhou 2018 | 17 | -0.84 [-1.53, -0.15] | Random | 0.002 | 98 |
| Trial registration in advance | Yes | Chen 2020, Gao 2022, Jin 2022, Li 2020B, Liu 2015, Lu 2021, Song 2020, Yu 2020, Zhan 2020, Zhou 2018 | 10 | -0.70 [-1.57, 0.17] | Random | 0.04 | 99 |
|  | No | Ao 2021, Chiu 1999, Ge 2021, Gu 2019, Huang 2018, Lan 2012, Wang 2017, Wu 2016, Yao 2015, Yin 2013, Zhang 2018 | 11 | -0.85 [-1.34, -0.37] | Random | < 0.001 | 94 |
| Risk of bias | Low risk | Chen 2020, Gao 2022, Gu 2019, Lan 2012, Li 2020B, Liu 2015, Lu 2021, Song 2020, Yao 2015, Yu 2020, Zhan 2020, Zhou 2018 | 12 | -0.75 [-1.47, -0.03] | Random | 0.007 | 99 |
|  | unclear/high risk | Ao 2021, Chen 2020, Gao 2022, Huang 2018, Jin 2022, Wang 2017, Wu 2016, Yin 2013, Zhang 2018 | 9 | -0.83 [-1.43, -0.24] | Random | < 0.001 | 95 |
| **Abbreviation:** N, number; WMD: weighted mean difference; CI: confidence interval; TEAS: Transcutaneous acupoint electrical stimulation | | | | | | | |

| **eTable 5: Meta-regression analysis for primary outcomes** | | | | |
| --- | --- | --- | --- | --- |
| **Subgroup** | **Number of studies** | **R^2 *^(%)** | **Wald Chi^2^** | ***p*** |
| **⦁ The cumulative intravenous morphine equivalent consumption within 24 hours after surgery** | | | | |
| The types of anaesthesia | N/A | | | |
| The types of surgery | 6 | 0 | 0.21 | 0.6437 |
| The implementation timing of TEAS | N/A | | | |
| The intervention of control group | 8 | 0 | 2.23 | 0.1351 |
| Trial registration in advance | 6 | 0 | 0.21 | 0.6437 |
| **⦁ Rest pain scores within 24 hours after surgery** | | | | |
| **2 hours** | | | | |
| The types of anaesthesia | N/A | | | |
| The types of surgery | 6 | 0.00 | 0.60 | 0.4376 |
| The implementation timing of TEAS | N/A | | | |
| The intervention of control group | 8 | 0.00 | 0.08 | 0.7829 |
| Trial registration in advance | 6 | 63.41 | 1.15 | 0.0416 |
| **6 hours** | | | | |
| The types of anaesthesia | N/A | | | |
| The types of surgery | 7 | 0.00 | 0.01 | 0.9220 |
| The implementation timing of TEAS | N/A | | | |
| The intervention of control group | 9 | 0.00 | 0.07 | 0.7876 |
| Trial registration in advance | 7 | 0.00 | 0.00 | 0.9725 |
| **12 hours** | | | | |
| The types of anaesthesia | N/A | | | |
| The types of surgery | 7 | 0.00 | 2.79 | 0.0946 |
| The implementation timing of TEAS | N/A | | | |
| The intervention of control group | N/A | | | |
| Trial registration in advance | N/A | | | |
| **24 hours** | | | | |
| The types of anaesthesia | 21 | 0.00 | 0.81 | 0.3669 |
| The types of surgery | 21 | 0.00 | 0.29 | 0.5884 |
| The implementation timing of TEAS | 21 | 0.00 | 0.47 | 0.4949 |
| The intervention of control group | 23 | 0.00 | 0.26 | 0.6081 |
| Trial registration in advance | 21 | 0.00 | 0.13 | 0.7146 |
| **^*^** An R^2^ value (coefficient of determination) was calculated to help quantify the extent of a covariate explained the variation in data. An R^2^=1 denoted that the covariate explained all the variability, while an R^2^=0 denoted that the covariate did not explain any of the variability. | | | | |
